# Rapid turnover of CTLA4 is associated with a complex architecture of reversible ubiquitylation

**DOI:** 10.1083/jcb.202312141

**Published:** 2024-10-15

**Authors:** Pei Yee Tey, Almut Dufner, Klaus-Peter Knobeloch, Jonathan N. Pruneda, Michael J. Clague, Sylvie Urbé

**Affiliations:** 1 Biochemistry, Cell and Systems Biology, Institute of Systems, Molecular and Integrative Biology, University of Liverpool, Liverpool, UK; 2 Institute of Neuropathology, Medical Faculty, University of Freiburg, Freiburg, Germany; 3 Signalling Research Centres BIOSS and CIBSS, University of Freiburg, Freiburg, Germany; 4 Department of Molecular Microbiology & Immunology, Oregon Health & Science University, Portland, USA

## Abstract

The immune checkpoint regulator CTLA4 is an unusually short-lived membrane protein. Here, we show that its lysosomal degradation is dependent on ubiquitylation at lysine residues 203 and 213. Inhibition of the v-ATPase partially restores CTLA4 levels following cycloheximide treatment, but also reveals a fraction that is secreted in exosomes. The endosomal deubiquitylase, USP8, interacts with CTLA4, and its loss enhances CTLA4 ubiquitylation in cancer cells, mouse CD4^+^ T cells, and cancer cell–derived exosomes. Depletion of the USP8 adapter protein, HD-PTP, but not ESCRT-0 recapitulates this cellular phenotype but shows distinct properties vis-à-vis exosome incorporation. Re-expression of wild-type USP8, but neither a catalytically inactive nor a localization-compromised ΔMIT domain mutant can rescue delayed degradation of CTLA4 or counteract its accumulation in clustered endosomes. UbiCRest analysis of CTLA4-associated ubiquitin chain linkages identifies a complex mixture of conventional Lys63- and more unusual Lys27- and Lys29-linked polyubiquitin chains that may underly the rapidity of protein turnover.

## Teaser

Tey et al. characterize the role of ubiquitin in the rapid turnover kinetics of the immune checkpoint protein CTLA4. A complex architecture of associated ubiquitin chains is revealed by a comprehensive ubiquitin chain restriction analysis in the absence of the deubiquitylase USP8.

## Introduction

The emergence of immunotherapy has dramatically changed the oncology landscape (Sanmamed and Chen, 2018). The cell surface receptor, CTLA4 (cytotoxic T-lymphocyte associated protein 4, CD152), provided the first such druggable immune checkpoint protein, offering positive outcomes in mouse models and clinical trials with CTLA4 blocking antibodies (Hodi et al., 2010; Kwon et al., 1997; Leach et al., 1996; Robert et al., 2011). Ipilimumab is a CTLA4 neutralizing antibody approved as a monotherapy for advanced melanoma, whereas a second CTLA4 antagonist, Tremelimumab, is approved for combination use with other checkpoint inhibitors in cases of liver cancer (de Castria et al., 2022; Hargadon et al., 2018; Korman et al., 2022). Whilst showing robust and durable immune protection in cancer patients, the clinical benefits of CTLA4 blockade are often overshadowed by severe immunotherapy-related adverse events (irAEs) that highly resemble autoimmune reactions (Martins et al., 2019; Postow et al., 2018). Despite the clinical prominence of CTLA4, critical aspects of its cell biology are underdeveloped. In distinction to other immune checkpoint molecules (e.g., PD-L1 and PD-1), it has one of the shortest known half-lives amongst transmembrane proteins (Li et al., 2021; Rusilowicz-Jones et al., 2022). A full understanding of its turnover may lead to novel therapeutic strategies.

CTLA4 is expressed constitutively in a subset of immunosuppressive regulatory T cells (T_reg_), while being induced in activated CD8^+^ and CD4^+^ T cells to limit and terminate T cell signaling (Linsley et al., 1992; Takahashi et al., 2000). Upon T cell activation, an intracellular pool of CTLA4 is mobilized to the cell surface where it displaces T cell co-receptor CD28 for interaction with their shared ligands on antigen-presenting cells (CD80 and CD86) (Linsley et al., 1996). T cell-associated CTLA4 has been shown to capture CD80 ligands from adjacent antigen-presenting cells in vivo for subsequent delivery to the lysosomes (Hou et al., 2015; Qureshi et al., 2011). It is now evident that CTLA4 expression is not exclusive to T cells; various tumors are reported to express CTLA4 and can also release CTLA4-containing exosomes (Contardi et al., 2005; Laurent et al., 2013; Mo et al., 2018; Paulsen et al., 2017; Theodoraki et al., 2019; Whiteside, 2013).

The dynamics of CTLA4 localization are strongly linked to physiological function, yet the molecular mechanisms governing its fate are incompletely understood. It is rapidly internalized from the cell surface in a clathrin- and dynamin-dependent manner to create a majority intracellular pool at steady-state (Follows et al., 2001; Qureshi et al., 2012). Furthermore, CTLA4 is turned over by lysosomal degradation, displaying a short half-life of ∼2 h in activated mouse transgenic T cells and ∼3 h in CTLA4-over-expressing CHO cells (Egen and Allison, 2002; Khailaie et al., 2018). In T cells, LPS Responsive Beige-Like Anchor Protein (LRBA) deflects CTLA4 from this degradative route toward recycling so that patients with LRBA deficiency suffer from early onset autoimmune disorders (Alroqi et al., 2018; Charbonnier et al., 2015; Lo et al., 2015). Otherwise, molecular details are fairly sparse. It is known to bind to the clathrin adaptors AP2 and AP1 for endocytosis and transport from the trans-Golgi network (TGN) to lysosomes, respectively (Leung et al., 1995; Qureshi et al., 2012; Schneider et al., 1999; Shiratori et al., 1997). Several Rab GTPases regulate CTLA4 sorting, with Rab5 and Rab7 mediating CTLA4 internalization and downstream degradation, Rab11 controlling CTLA4 recycling and Rab8 regulating CTLA4 transport to the cell surface (Banton et al., 2014; Janman et al., 2021).

Ubiquitylation frequently provides a critical sorting signal by which receptors interact with the endosomal sorting complexes required for transport (ESCRT) machinery and commit to lysosomal degradation (Clague and Urbé, 2017; Piper et al., 2014). The receptors are then incorporated into luminal vesicles of multivesicular bodies (MVBs) before fusion with lysosomes (Futter et al., 1996). Two endosomal deubiquitylating enzymes (DUBs), USP8 (UBPY), and AMSH (associated molecule with the SH3 domain of STAM; STAM binding protein, STAMBP), interact with the ESCRT machinery and can differentially influence the fate of endocytosed receptors (Clague et al., 2012; Clague and Urbé, 2006). It is known that CTLA4 can be ubiquitylated, but not whether this ubiquitylation influences endosomal sorting. Some circumstantial evidence supporting this hypothesis is provided by the induction of CTLA4 ubiquitylation upon engagement of its ligand, the transmembrane protein CD80, which is targeted for destruction by trans-endocytosis (Kennedy et al., 2022). Here, we first show that CTLA4 is endogenously expressed as a short-lived protein in selected cancer cell lines, where it is degraded in lysosomes. We demonstrate that direct ubiquitylation at two specific lysine residues controls CTLA4 lysosomal degradation. We uncover a critical role for USP8 and its adapter protein HD-PTP in regulating CTLA4 ubiquitylation status in multiple settings, whilst ubiquitin chain restriction (UbiCRest) analysis reveals a distinctive architecture of CTLA4-associated polyubiquitin chains (Hospenthal et al., 2015).

## Results

### CTLA4 ubiquitylation promotes its rapid constitutive degradation

**Figure S1.**
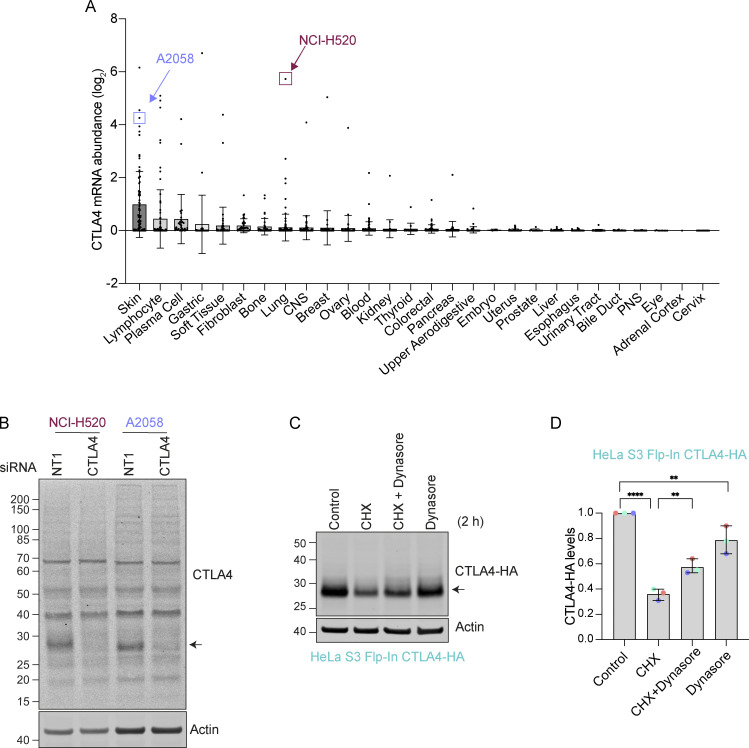
**CTLA4 expression in cancer cell lines. (A)** mRNA levels derived from the Cancer Cell Line Encyclopedia (CCLE) database (Barretina et al., 2012). Each dot represents an individual tumor cell line within the indicated categories. **(B)** Representative western blot of NCI-H520 and A2058 cells transfected with siRNA against non-targeting (NT1) or CTLA4 for 72 h prior to lysis. **(C)** Representative western blot of HeLa S3 Flp-In CTLA4-HA cells treated with Dynasore (80 µM) for 30 min in serum-free media before the addition of Cycloheximide (CHX, 100 µg/ml) for 2 h. **(D)** Quantification of CTLA4-HA levels for data represented in C. Individual data points from three independent, color-coded experiments are shown. Error bars indicate SD. One-way ANOVA and Dunnett’s multiple comparison test, **P < 0.01, ****P < 0.0001. Source data are available for this figure: SourceData FS1.

**Figure 1.**
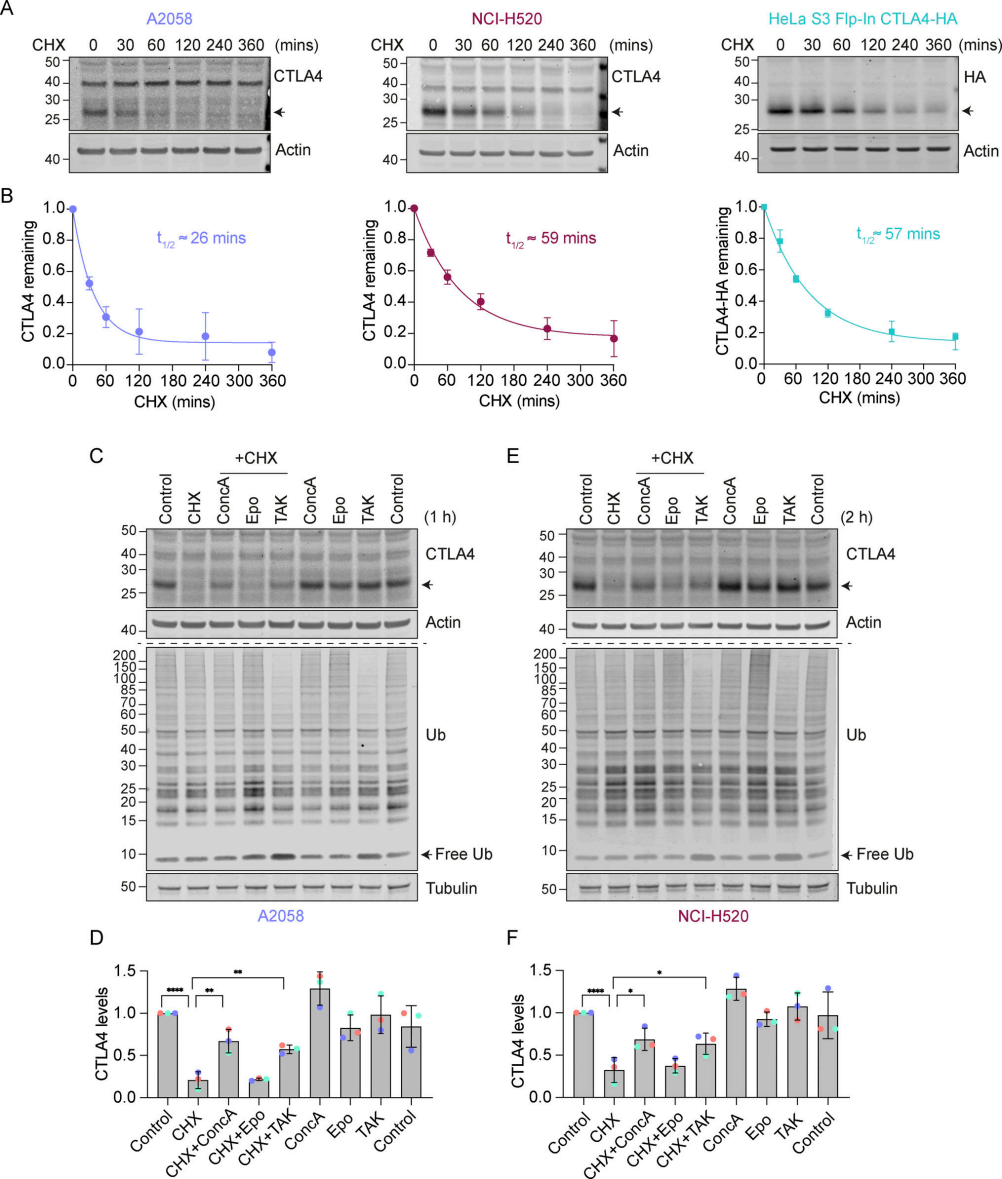
**Lysosomal degradation of CTLA4 requires a functional ubiquitin conjugation cascade. (A)** Representative western blots of A2058, NCI-H520, and HeLa S3 Flp-In CTLA4-HA cells treated with Cycloheximide (CHX, 100 µg/ml) for indicated times prior to lysis. **(B)** Quantification of data represented in A. The half-life of CTLA4 in different cell lines was estimated using an exponential decay model. Error bars indicate SD from three independent experiments. **(C and E)** Representative western blots of A2058 and NCI-H520 cells treated with Concanamycin (ConcA, 100 nM), Epoxomicin (Epo, 100 nM), or TAK-243 (TAK, 1 µM) for 15 min before the addition of CHX for 1 h (C, A2058) or 2 h (E, NCI-H520). **(D and F)** Quantification of CTLA4 levels after treatment with indicated inhibitors relative to control for data represented in C and E. Individual data points from three independent, color-coded experiments are shown. Error bars show SD. One-way ANOVA and Dunnett’s multiple comparisons test, *P < 0.05, **P < 0.01, ****P < 0.0001. Source data are available for this figure: SourceData F1.

Individual reports indicate that a variety of tumor cells express endogenous CTLA4 (Chen et al., 2017; Laurent et al., 2013; Mo et al., 2018; Paulsen et al., 2017). We consulted the cancer cell line encyclopedia (CCLE) database for cancer cell lines expressing CTLA4 and selected one melanoma (A2058) and one squamous lung carcinoma (NCI-H520) cell line for further investigation (Fig. S1 A) (Barretina et al., 2012). Western blot analysis of total cell lysates prepared from these cells treated with non-targeting control (NT1) or CTLA4-selective siRNA confirmed expression in both lines (Fig. S1 B). In parallel, we generated Flp-In HeLa cells that constitutively express an epitope-tagged form, CTLA4-HA. A cycloheximide time course established that CTLA4 has an extremely short half-life (<1 h) in all three cell lines, which makes it one of the most short-lived transmembrane proteins described in human cancer cells (Fig. 1, A and B) (Li et al., 2021; Rusilowicz-Jones et al., 2022).

We next set out to define the degradation route for CTLA4 in these cells using established inhibitors of the proteasome (Epoxomicin), lysosome (Concanamycin A; v-ATPase inhibitor), and ubiquitin conjugation machinery (TAK243; UBA1 inhibitor). Both Concanamycin A (ConcA) and TAK-243 treatment rescued endogenous CTLA4 protein from degradation under Cycloheximide chase conditions in melanoma and lung cancer cells, whereas the proteasome inhibitor (Epo) was without effect (Fig. 1, C–F). Probing the samples for ubiquitin revealed that proteasome and ubiquitin E1 inhibitors increase and decrease ubiquitin conjugates as expected. These data indicate that CTLA4 is constitutively targeted for lysosomal degradation in a ubiquitin-dependent fashion. This sorting pathway has been well-described for other transmembrane proteins, e.g., activated epidermal growth factor receptor (EGFR), and involves ubiquitin- and ESCRT machinery-dependent packaging of cargo into internal vesicles of MVBs (Henne et al., 2011; Hurley and Stenmark, 2011).

**Figure S2.**
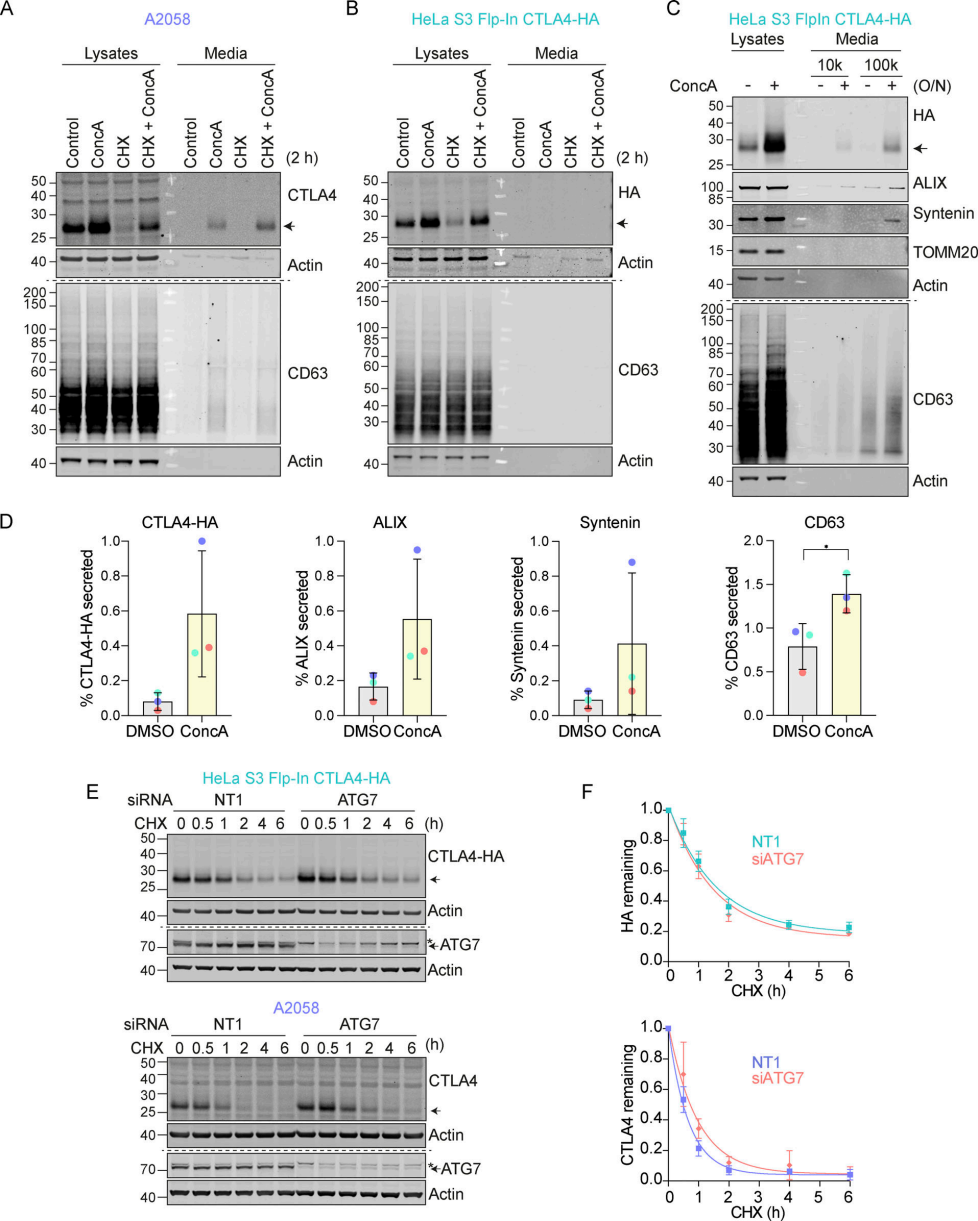
**CTLA4 is secreted via exosomes from A2058 and HeLa S3 Flp-In CTLA4-HA cells. (A and B)** Representative western blots of the lysates and cultured supernatants from mock- and Concanamycin A (ConcA)-treated A2058 and HeLa S3 Flp-In CTLA4-HA cells. Cells were lysed and media were subjected to trichloroacetic acid (TCA) precipitation. **(C)** Representative western blots of the lysates and cultured supernatants from mock- and ConcA-treated HeLa S3 Flp-In CTLA4-HA cells for indicated times. Cells were lysed and the media was collected by serial centrifugation to concentrate extracellular vesicles (100k pellet, exosome fraction). **(D)** Quantification of CTLA4, ALIX, Syntenin and CD63 secreted in the exosome fractions (100k) for data represented in C. Individual data points from three independent, color-coded experiments are shown. Error bars show SD. **(E)** Representative western blot of Cycloheximide (CHX; 100 µg/ml) chase experiment in HeLa S3 Flp-In CTLA4-HA and A2058 cells, following treatment with non-targeting (NT1) or ATG7 siRNA for 72 h. * indicates a non-specific band. **(F)** Quantification of blots represented in E. Error bars show SD from three independent experiments. Source data are available for this figure: SourceData FS2.

**Figure 2.**
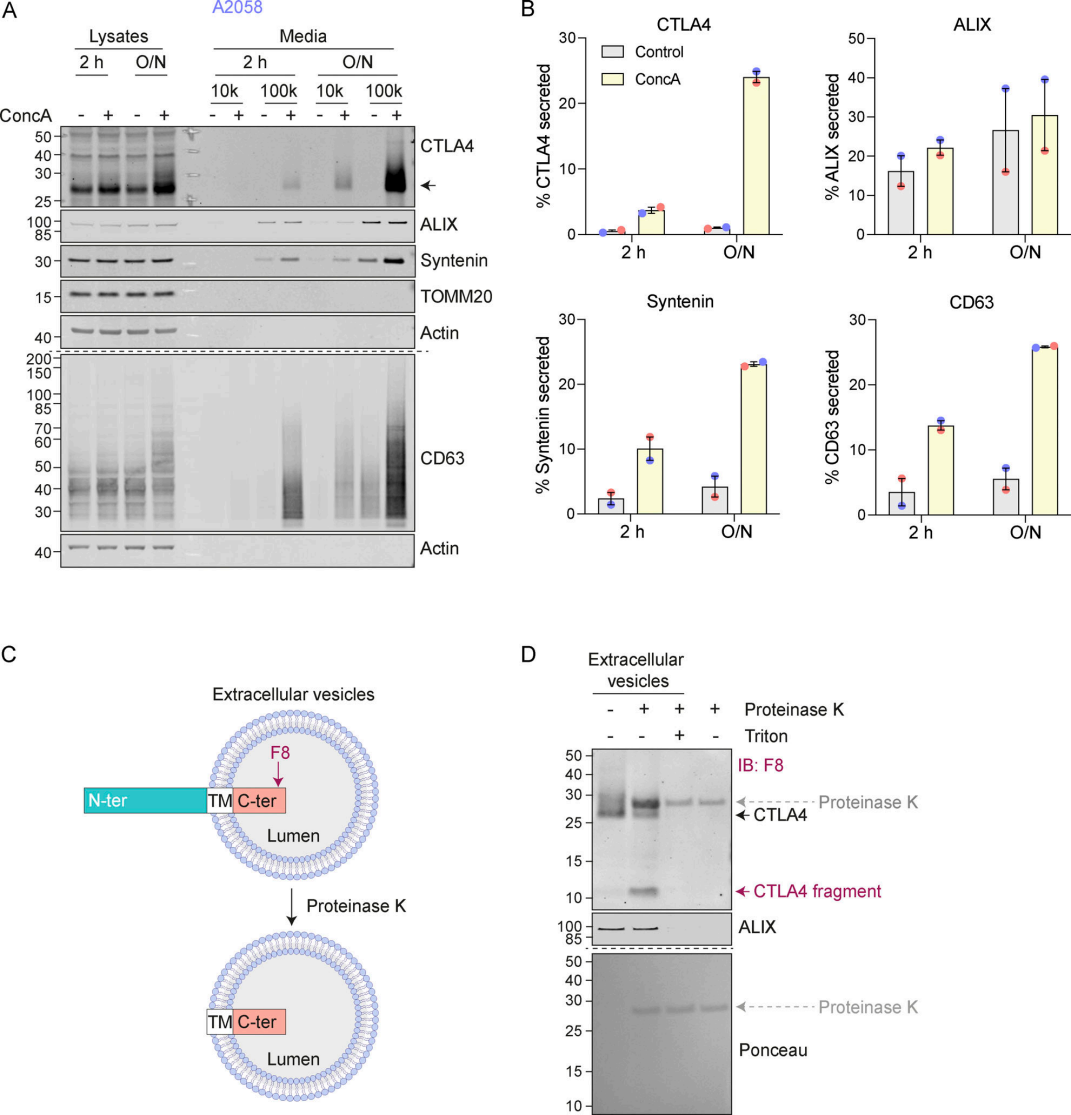
**CTLA4 is secreted via exosomes in response to v-ATPase inhibition. (A)** Representative western blots of lysates and culture supernatants from mock- and Concanamycin A-(ConcA, 100 nM) treated A2058 cells (O/N: overnight). Cells were lysed and the media collected by serial centrifugation to concentrate extracellular vesicles (100k pellet, exosome fraction). **(B)** Quantification of CTLA4, ALIX, Syntenin, and CD63 recovered in the exosome fractions (100k) for data represented in A. Individual data points from two independent, color-coded experiments are shown. Error bars indicate range. **(C)** Predicted topology of CTLA4 in exosomal membranes. F8: C-terminal (C-ter) cytoplasmic domain directed CTLA4 antibody; TM, transmembrane domain; N-ter, N-terminal domain. **(D)** Representative western blots and Ponceau staining of samples from a Proteinase K protection assay of exosome-associated CTLA4. IB: Immunoblot. Source data are available for this figure: SourceData F2.

We noticed that in melanoma and lung cancer cells, blocking the lysosomal degradation pathway with ConcA only partially rescued constitutive CTLA4 degradation (Fig. 1, C–F and Fig. S2 A). We wondered whether a fraction of CTLA4 might not be degraded but rather secreted via the release of exosomes (van Niel et al., 2018). These small extracellular vesicles are derived from the internal vesicles of specialized MVBs that can fuse with the plasma membrane, and their release has previously been shown to be dramatically enhanced by v-ATPase inhibitors (Edgar et al., 2016). Harvesting conditioned media from A2058 cells treated for either 2 h or overnight (O/N) with ConcA revealed secretion of established exosome markers CD63 and Syntenin (SDCBP) as well as CTLA4 (>20% of the total, after O/N ConcA treatment) into the medium (Fig. 2, A and B; and Fig. S2 A). In HeLa cells, this pathway is less prominent; ConcA fully rescued CTLA4 protein levels in lysates of CHX-treated cells, and an overnight treatment was necessary to visualize exosome and CTLA4-HA secretion (Fig. S2, B–D). Both autophagy and endocytic degradation pathways rely on lysosomal acidification. We have excluded a role for autophagy in CTLA4 degradation by efficiently depleting the core autophagy component ATG7 with no significant loss of degradation (Fig. S2, E and F). A protease protection assay confirmed the expected orientation of CTLA4 in the exosome fraction with an exposed amino terminus (Fig. 2, C and D).

### USP8 depletion enhances CTLA4 ubiquitylation but delays its degradation

**Figure 3.**
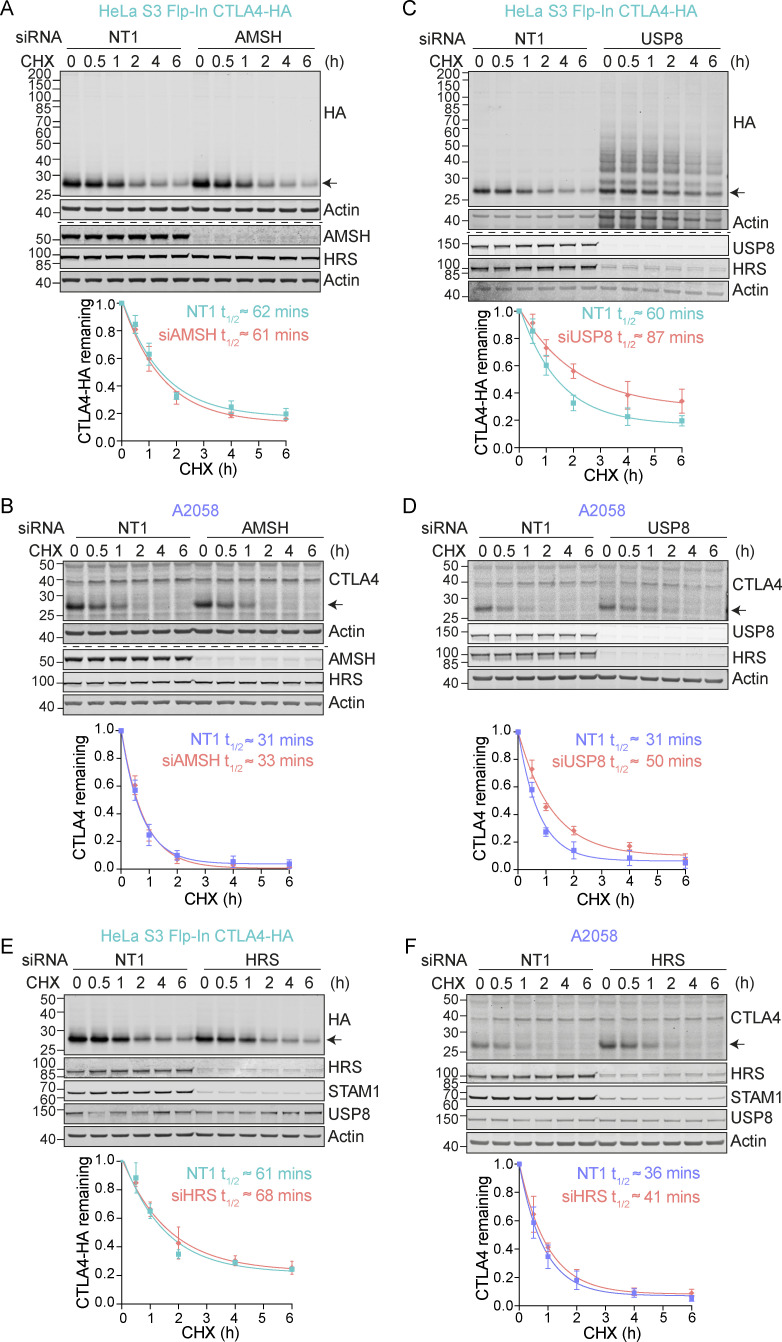
**CTLA4 degradation is delayed in the absence of USP8. (A–F)** Representative western blots and associated quantifications of Cycloheximide (CHX; 100 µg/ml) chase experiments in HeLa S3 Flp-In CTLA4-HA (A, C, and E) and A2058 (B, D, and F) cells following treatment with non-targeting (NT1), AMSH, USP8, or HRS siRNA for 72 h. The half-life of CTLA4 was estimated using an exponential decay model. Error bars show SD from three independent experiments. Source data are available for this figure: SourceData F3.

Having established that ubiquitylation is required for lysosomal targeting and degradation of CTLA4, we reasoned that its trafficking may be regulated by endosome-associated DUBs, AMSH, or USP8 (Clague and Urbé, 2006, 2017). AMSH is a highly selective enzyme specializing in removing Lys63-linked ubiquitin chains, whereas USP8 cleaves a wide range of ubiquitin chain types (McCullough et al., 2006; Ritorto et al., 2014; Row et al., 2006). We used RNAi to deplete each of these DUBs and monitored the turnover of CTLA4 using a cycloheximide chase. Both endogenous and heterologously expressed CTLA4(-HA) were insensitive to AMSH depletion (Fig. 3, A and B). In contrast, siRNA targeting of USP8 increased CTLA4 half-life in both cell lines (Fig. 3, C and D). We have previously shown that USP8 not only deubiquitylates endolysosomal cargo but also stabilizes the ESCRT-0 components HRS and STAM (Clague and Urbé, 2006; Row et al., 2006). Loss of HRS, upon USP8 depletion, is clearly apparent in both cell lines studied here (Fig. 3, C and D). However, the depletion of HRS itself had very little impact on CTLA4 turnover, thus establishing an ESCRT-0 independent role for USP8 in regulating CTLA4 degradation (Fig. 3, E and F).

**Figure 4.**
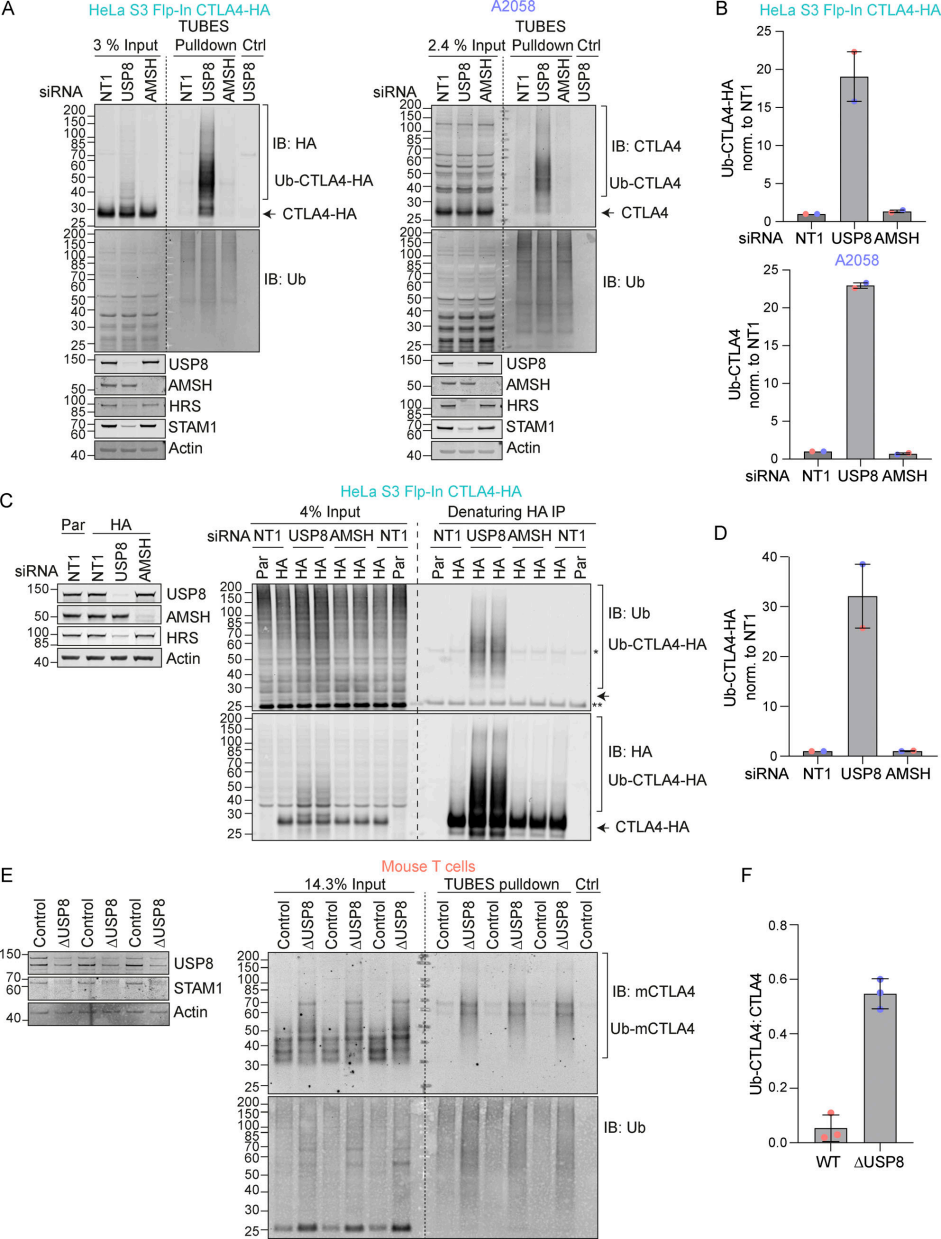
**USP8 depletion dramatically enhances CTLA4 ubiquitylation. (A)** Representative western blots of TUBES pulldown of ubiquitylated CTLA4. HeLa S3 Flp-In CTLA4-HA and A2058 cells were transfected for 72 h with non-targeting (NT1), USP8, and AMSH siRNA prior to lysis. Lysates were subjected to TUBES-pulldown (IB: Immunoblot). **(B)** Quantification of ubiquitylated CTLA4-HA isolated by TUBES pulldown for data represented in A. Ubiquitylated CTLA4-HA was normalized to total ubiquitin pulled down. Individual data points from two independent, color-coded experiments are shown. Error bars indicate the range. **(C)** Representative western blots of ubiquitylated CTLA4-HA immunoprecipitated under denaturing conditions. HeLa S3 Flp-In parental (Par) or CTLA4-HA (HA) cells were transfected for 72 h with non-targeting (NT1), USP8, and AMSH siRNA. Cells were lysed in denaturing SDS lysis buffer and lysates were subjected to immunoprecipitation (IP) with anti-HA coupled magnetic beads. *Antibody heavy chain; ** antibody light chain. **(D)** Quantification of CTLA4-HA ubiquitylation relative to NT1 for data represented in C. Ubiquitylated CTLA4-HA was normalized to total immunoprecipitated CTLA4-HA. Individual data points from two independent, color-coded experiments are shown. Error bars indicate the range. **(E)** Representative western blots of TUBES pulldown of ubiquitylated CTLA4. Lysates from USP8 fl/fl (Control, *n* = 3) and USP8 deleted (∆USP8, *n* = 3) activated T cells derived from individual mice were either analyzed directly by SDS-PAGE and western blot (left blot), or first subjected to a TUBES pulldown prior to analysis alongside input samples. **(F)** Quantification of ubiquitylated CTLA4 enriched by TUBES pulldown for data represented in E. Ubiquitylated CTLA4 was ratioed to total CTLA4 levels. Individual data points from six individual mice are shown. Error bars indicate SD. Source data are available for this figure: SourceData F4.

**Figure S3.**
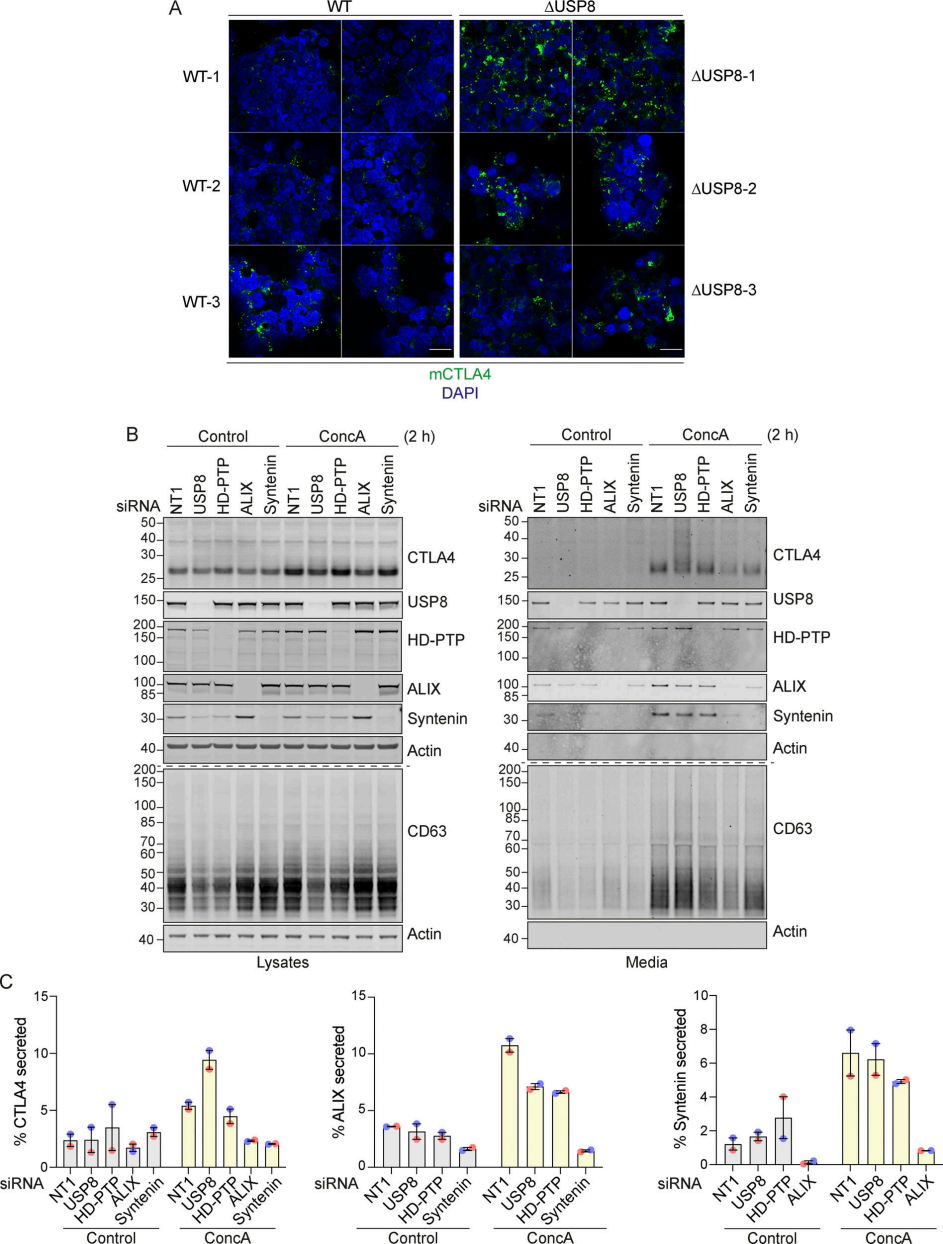
**CTLA4 accumulation in USP8-deleted mouse CD4 T cells and secretion via exosomes from USP8-depleted melanoma cells. (A)** Representative Airyscan images (two fields of view each) of CTLA4 co-stained with DAPI in USP8 fl/fl (WT, 1–3) and USP8 deleted (∆USP8, 1–3) activated T cells derived from individual mice. Scale bar = 15 µm. **(B)** A2058 cells were transfected for 72 h with non-targeting (NT1) or USP8, HD-PTP, ALIX, and Syntenin siRNAs. Lysates were collected and the cultured supernatant subjected to TCA precipitation before analysis by SDS-PAGE and western blot. **(C)** Quantification of CTLA4 secreted into the media for data represented in B. Individual data points from two independent, color-coded experiments are shown. Error bars indicate the range. Source data are available for this figure: SourceData FS3.

In the absence of USP8, we noticed a series of higher molecular weight bands, indicative of ubiquitylated species of CTLA4 that were most apparent for CTLA4-HA (Fig. 3 C). We next used two separate approaches to assess whether CTLA4 was ubiquitylated in these cells. First, we enriched ubiquitylated proteins using a Tandem Ubiquitin Binding Entities (TUBES) pulldown and probed for CTLA4 (Fig. 4, A and B) (Mattern et al., 2019). Secondly, we immunoprecipitated CTLA4-HA from denatured HeLa cell lysates and probed for ubiquitin (Fig. 4, C and D). Together, these experiments demonstrate that a fraction of CTLA4 is ubiquitylated at steady state, whilst depletion of USP8 but not AMSH dramatically increases this ubiquitylated pool. We also took advantage of a mouse model for conditional USP8 deletion (∆USP8). T cells derived from these mice can be cultured in vitro and treated with Tamoxifen to elicit USP8 deletion. ∆USP8 mice show higher levels of CTLA4 by both Western blotting and by immunofluorescence which additionally highlights the intracellular accumulation (Fig. 4 E and Fig. S3 A). It is also immediately apparent that a fraction of CTLA4 runs as a higher molecular weight species upon USP8 deletion in T cell populations isolated from three distinct sets of mice (Fig. 4 E). A TUBES pulldown confirmed that these species correspond to ubiquitylated CTLA4 and enabled quantitation relative to the total CTLA4 in the input samples (Fig. 4 F).

### Clathrin adaptor AP2-dependent endocytosis is required for CTLA4 ubiquitylation and degradation

**Figure 5.**
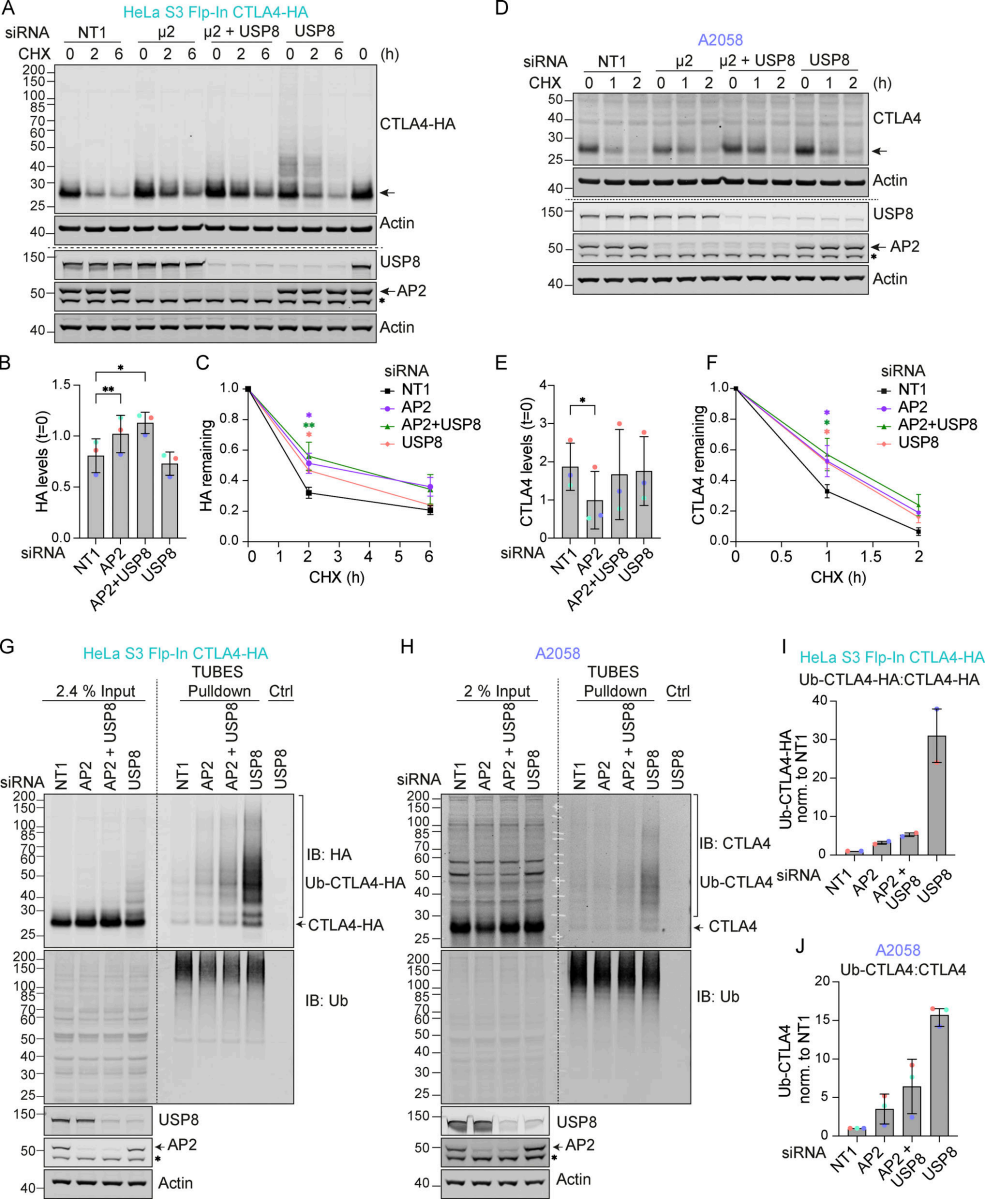
**AP2 depletion delays CTLA4 turnover and reduces CTLA4 ubiquitylation in the absence of USP8. (A and D)** Representative western blots following treatment with Cycloheximide (CHX; 100 µg/ml) for indicated times in HeLa S3 Flp-In CTLA4-HA (A) and A2058 (D) transfected with indicated siRNAs. Cells were transfected with non-targeting (NT1) or AP2 siRNA targeting the µ2 subunit and reseeded for a second round of transfection 24 h later with NT1, AP2, and/or USP8 siRNA. The cells were left for a further 72 h before lysis. The last lane represents untransfected control. *non-specific signal. **(B and E)** Quantification of basal CTLA4 levels normalized to untransfected control for data represented in A and D. Error bars show SD from three independent experiments. One-way ANOVA with Dunnett’s multiple comparisons test, *P < 0.05, **P < 0.01. **(C and F)** Quantification of CTLA4 levels following treatment with Cycloheximide for data represented in A and D. One-way ANOVA with Dunnett’s multiple comparisons test, *P < 0.05, **P < 0.01. **(G and H)** Representative western blots of TUBES pulldown of ubiquitylated CTLA4. HeLa S3 Flp-In CTLA4-HA and A2058 cells were transfected as in A and D. IB: immunoblot. *non-specific signal. **(I and J)** Quantification of ubiquitylated CTLA4 relative to total CTLA4 levels for data represented in G and H. Individual data points from two (I) or three (J) independent, color-coded experiments are shown. Error bars indicate range (*n* = 2) or SD (*n* = 3). Source data are available for this figure: SourceData F5.

Endocytic trafficking of CTLA4 is known to be clathrin- and dynamin-dependent, utilizing the AP2 adaptor protein for incorporation into clathrin-coated vesicles (CCVs) (Qureshi et al., 2012; Shiratori et al., 1997). In our HeLa cell model, treatment with Dynasore, a drug that inhibits dynamin-dependent receptor endocytosis, increases the stability of CTLA4 (Fig. S1, C and D) (Macia et al., 2006; Omerovic et al., 2012). We have more specifically depleted the µ2 subunit of AP2 using an oligonucleotide whose blocking of Transferrin Receptor endocytosis has been extensively characterized by the Robinson group (Motley et al., 2003). In the HeLa cell model, loss of AP2 leads to elevated steady-state levels of CTLA4 and slower turnover, similar to the loss of USP8 (Fig. 5, A–C), with no additive effect seen with the combined depletion. In the A2058 melanoma model, we see reduced steady-state levels upon AP2 depletion, but recapitulate the retardation of protein decay following cycloheximide treatment (Fig. 5, D–F). Most striking is the strong contingency of the ubiquitylation signal revealed by USP8 depletion, which is completely lost when accompanied by depletion of AP2 (Fig. 5, A and G–J). This finding is shared between cell models when processed through a TUBE pull-down step to manifest the signal. Thus, CTLA4 ubiquitylation occurs at the endosome and the USP8-sensitive component of degradation lies downstream of AP2-mediated endocytosis.

### HD-PTP and USP8 cooperate to govern CTLA4 ubiquitylation

**Figure 6.**
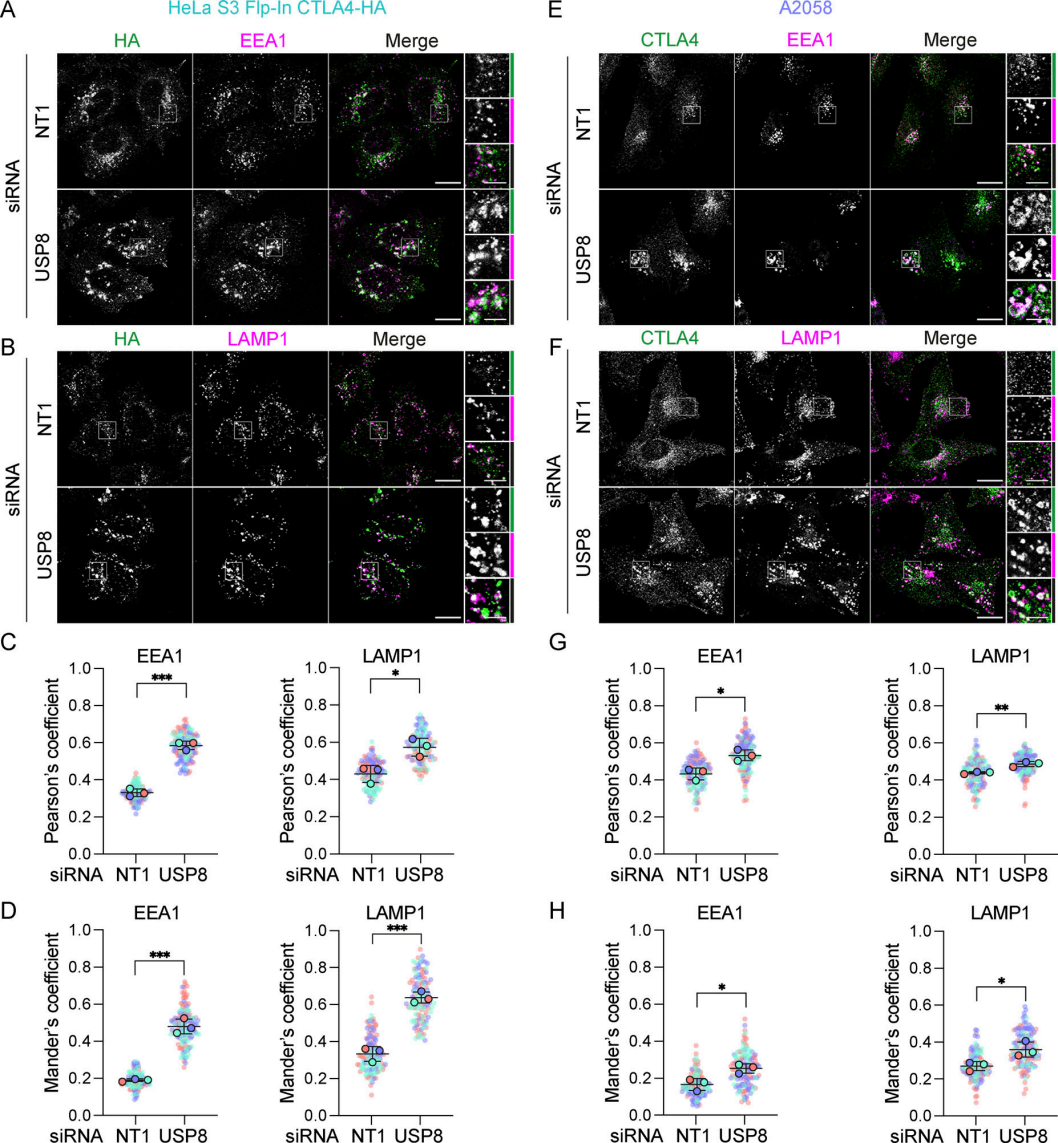
**CTLA4 accumulates in enlarged endosomes following USP8 depletion. (A and B)** Representative confocal images of CTLA4-HA co-stained for EEA1 (A) or LAMP1 (B) in HeLa S3 Flp-In CTLA4-HA cells transfected for 72 h with non-targeting (NT1) or USP8 siRNA. Scale bar = 15 µm (main figure) or 5 µm (inset). **(C and D)** Colocalization analysis of CTLA4-HA with EEA1 and LAMP1 in HeLa S3 Flp-In CTLA4-HA for data represented in A and B. Shown are the Pearson’s (C) or Mander’s coefficients (D). Error bars show SD from three independent, color-coded experiments. Opaque circles with black outlines correspond to the mean value from each experiment. Unpaired *t* test. *P < 0.05, ***P < 0.001. **(E and F)** Representative confocal images of CTLA4 co-stained for EEA1 (E) or LAMP1 (F) in A2058 cells transfected for 72 h with NT1 or USP8 siRNA. Scale bar = 15 µm (main figure) or 5 µm (inset). **(G and H)** Colocalization analysis of CTLA4 with EEA1 and LAMP1 in A2058 cells for data represented in E and F. Graphs show Pearson’s (G) or Mander’s coefficients (H) between CTLA4 and EEA1 or LAMP1. Error bars show SD from three independent, color-coded experiments. Opaque circles with black outlines correspond to the mean value from each experiment. Unpaired *t* test. *P < 0.05, **P < 0.01.

**Figure 7.**
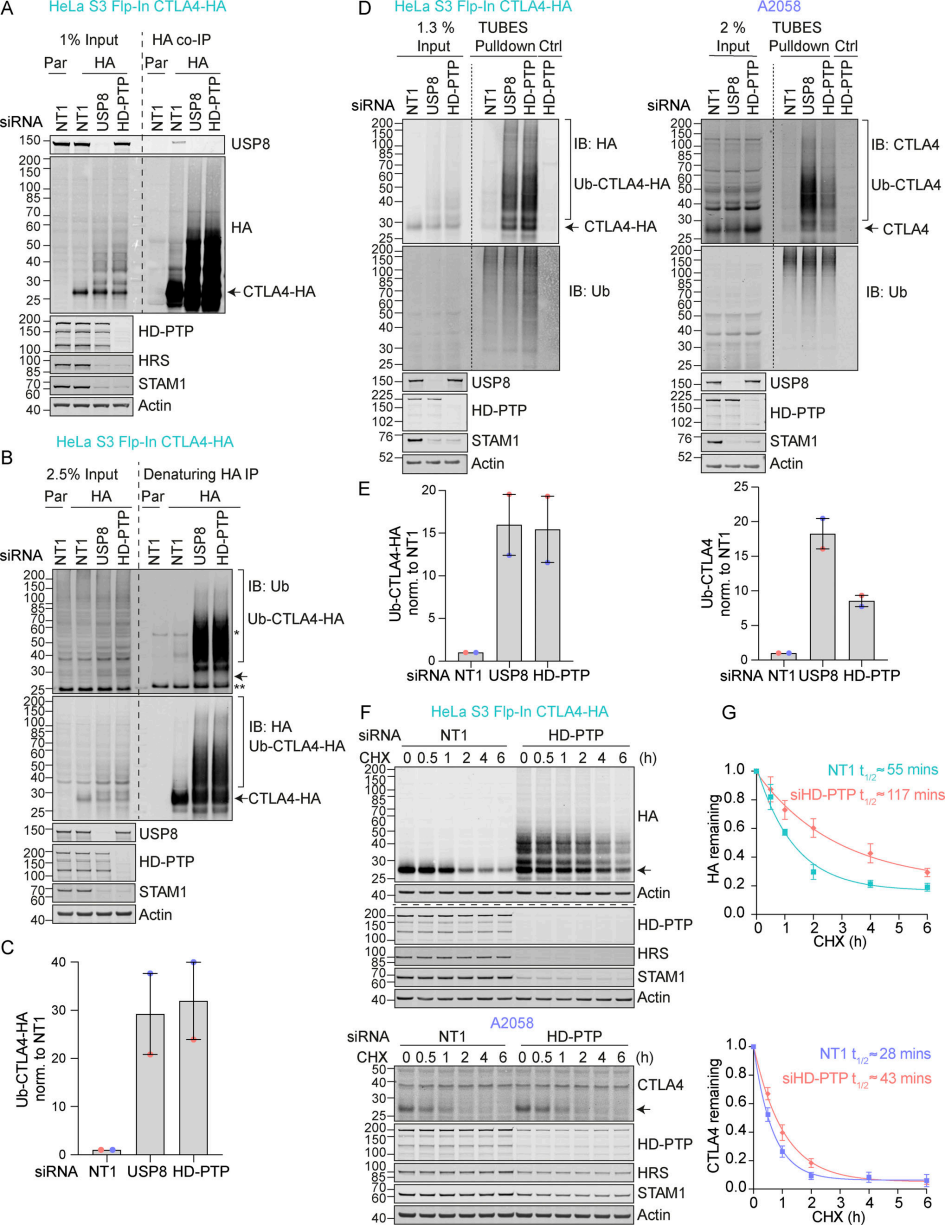
**Regulation of CTLA4 ubiquitylation by USP8 is dependent on HD-PTP. (A)** Representative western blots of USP8 co-immunoprecipitated with CTLA4-HA. HeLa S3 Flp-In parental (Par) or CTLA4-HA (HA) cells were transfected for 72 h with non-targeting (NT1), USP8 or HD-PTP siRNA. Cells were lysed and lysates were subjected to HA-immunoprecipitation (IP; IB: Immunoblot). **(B)** Representative western blots of ubiquitylated CTLA4-HA immunoprecipitated under denaturing conditions. HeLa S3 Flp-In parental (Par) or CTLA4-HA (HA) cells were transfected as in A. Cells were lysed in denaturing SDS lysis buffer and lysates were subjected to immunoprecipitation (IP) with anti-HA coupled magnetic beads. *Antibody heavy chain; ** antibody light chain. **(C)** Quantification of CTLA4-HA ubiquitylation relative to NT1 for data represented in B. Ubiquitylated CTLA4-HA was normalised to total CTLA4-HA pulled down. Individual data points from two independent, color-coded experiments are shown. Error bars indicate the range. **(D)** Representative western blots of TUBES pulldown of ubiquitylated CTLA4. HeLa S3 Flp-In CTLA4-HA and A2058 cells were transfected as in A and B and cell lysates subjected to TUBES pulldown. **(E)** Quantification of CTLA4-HA ubiquitylation relative to NT1 for data represented in D. Ubiquitylated CTLA4(-HA) was normalized to total ubiquitin pulled down. Individual data points from two independent, color-coded experiments are shown. Error bars indicate the range. **(F)** Representative western blots of CHX chase in HeLa S3 Flp-In CTLA4-HA and A2058 cells following transfection with non-targeting (NT1) or HD-PTP siRNA. Cells were treated with CHX for indicated times before lysis. **(G)** The half-life of CTLA4 was estimated using an exponential decay model. Error bars indicate SD from three independent experiments. Source data are available for this figure: SourceData F7.

Immunofluorescence microscopy of USP8-depleted HeLa and A2058 cells reveals the typical enlarged or clustered endosomal morphology as previously reported (Niendorf et al., 2007; Row et al., 2006). CTLA4 accumulates, on both EEA1 and LAMP1-positive structures marking early and late endosomes respectively (Fig. 6, A–H). USP8 associates with multiple interaction partners at endosomal membranes, including STAM1 and 2, several CHMPs, HD-PTP (PTPN23), and ubiquitylated cargo itself (Clague and Urbé, 2006). Of these, HD-PTP has previously been shown to be essential for USP8 recruitment to activated receptors at endosomes (Ali et al., 2013; Ma et al., 2015; Parkinson et al., 2021). We could detect a small fraction of endogenous USP8 associated with CTLA4-HA that was abolished by HD-PTP depletion (Fig. 7 A). Consequently, the loss of HD-PTP mirrors that of USP8 by promoting CTLA4-HA ubiquitylation and increasing its half-life (Fig. 7, A–G). This further corroborates the role of HD-PTP as an obligatory adapter for USP8 recruitment. In A2058 cells the same effects of HD-PTP depletion upon ubiquitylation and half-life of endogenous CTLA4 are evident, albeit less strong than with USP8 depletion (Fig. 7, D–G). As A2508 cells allow ready assessment of exosomal release, we tested the effects of USP8 and HD-PTP depletion on this process. The ConcA-induced release of CTLA4 is dependent on the exosomal sorting factors Syntenin and ALIX, whilst USP8 depletion enhances both the amount and ubiquitylation status of CTLA4 released into the media without altering the exosomal pool of Syntenin (Fig. S3, B and C). HD-PTP depletion is without effect on CTLA4-secretion, providing the first dissociation of USP8 and HD-PTP cellular phenotypes, perhaps reflecting another USP8 recruitment mode associated with this exosome-directed aspect of USP8 function.

**Figure 8.**
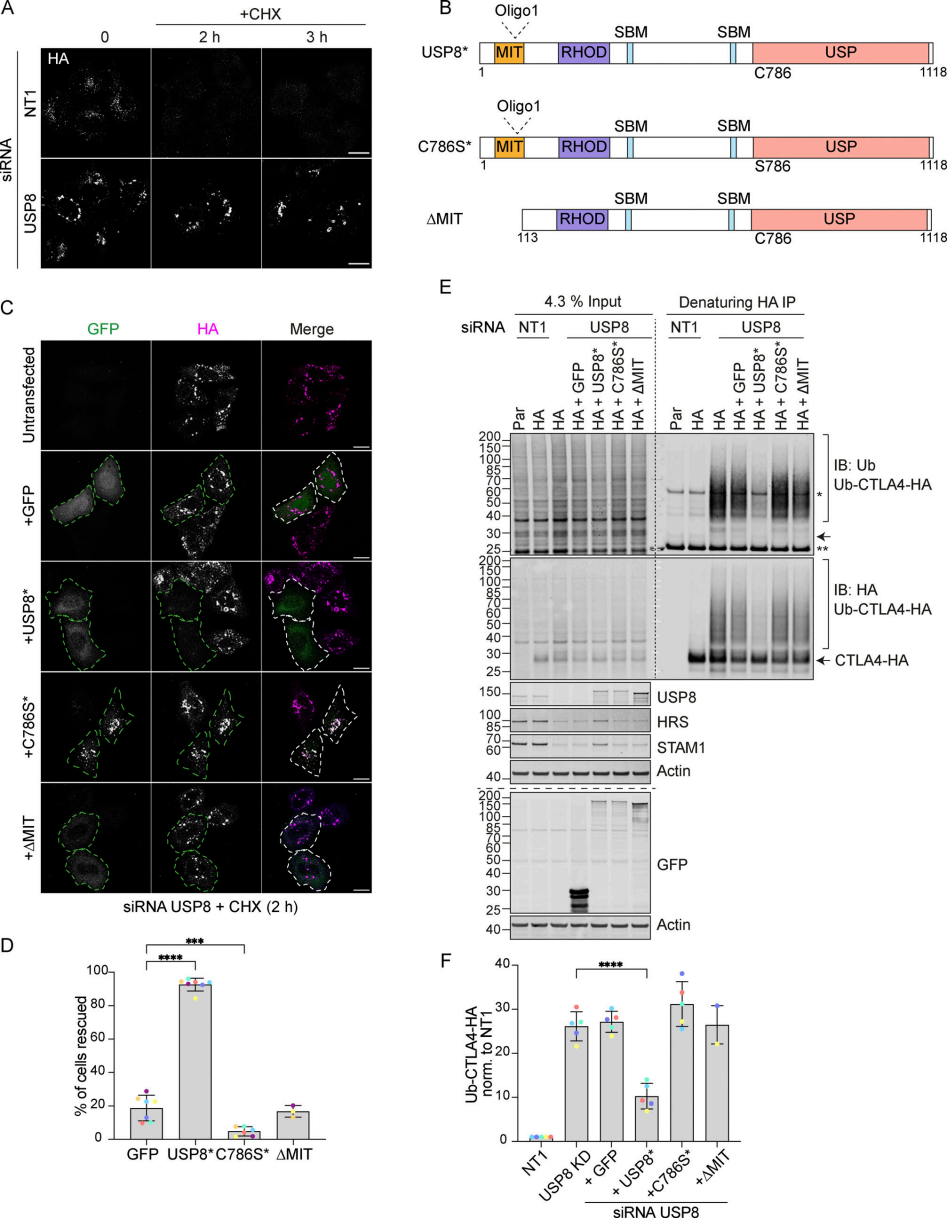
**Catalytic activity and endosomal localization of USP8 are essential for CTLA4 degradation and ubiquitylation. (A)** Representative confocal images of HeLa S3 Flp-In CTLA4-HA transfected for 72 h with NT1 or USP8 siRNAs before Cycloheximide (CHX) treatment for indicated times. Cells were fixed and stained for HA. Scale bar = 15 µm. **(B)** GFP-tagged USP8 siRNA-resistant (USP8*) constructs used in this study. MIT, Microtubule interacting; SBD: SH3 domain binding motif; RHOD, Rhodanese homology domain; USP, Ubiquitin specific protease - catalytic domain. **(C)** Representative confocal images of HeLa S3 Flp-In CTLA4-HA cells transfected with non-targeting (NT1) or USP8 siRNA and GFP, siRNA-resistant GFP-tagged USP8 (USP8*), catalytically inactive USP8 (C786S*) or ∆MIT-USP8. Cells were treated for 2 h with CHX prior to fixation and staining for HA. Scale bar = 15 µm. **(D)** Quantification of cells showing rescued phenotypes calculated for data represented in C. Individual data points from 3 (∆MIT), 6 (C786S), or 7 (GFP and USP8*) independent, color-coded experiments are shown. Error bars indicate the SD. Total number of cells analyzed: GFP (599); USP8* (666); C786S (486); ∆MIT (322). One-way ANOVA and Dunnett’s multiple comparison test, ***P < 0.001 and ****P < 0.0001. **(E)** Representative western blots of ubiquitylated CTLA4-HA immunoprecipitated under denaturing conditions. HeLa S3 Flp-In parental (Par) or CTLA4-HA (HA) cells were transfected with NT1 or USP8 siRNA and either GFP or GFP-tagged siRNA-resistant USP8 constructs as in C. Cells were lysed in denaturing SDS lysis buffer and lysates were subjected to immunoprecipitation (IP) with anti-HA coupled magnetic beads. *Antibody heavy chain; ** antibody light chain. IB: Immunoblot. (**F)** Quantification of CTLA4-HA ubiquitylation relative to NT1 for data represented in E. Ubiquitylated CTLA4-HA was normalized to total CTLA4-HA pulled down. Individual data points from two (∆MIT) or five independent, color-coded experiments are shown. Error bars indicate the SD (*n* = 5) or range (*n* = 2). One-way ANOVA and Dunnett’s multiple comparison test,****P < 0.0001. Source data are available for this figure: SourceData F8.

The multidomain structure of USP8 and intricate low-affinity interaction network ensures the assembly of the many ESCRT-machinery components through co-incidence detection (Row et al., 2007). It is conceivable that it is the loss of USP8 as a scaffold that is relevant for efficient CTLA4 sorting to the lysosome, as opposed to its catalytic DUB function. To formally establish whether the DUB activity of USP8 is essential for CTLA4 trafficking and degradation, we conducted a series of rescue experiments using siRNA-resistant GFP-tagged USP8 constructs. These were aimed at correcting CTLA4 turnover and endolysosomal accumulation, which can be visually assessed (Fig. 8, A and B). As well as a catalytically inactive mutant (USP8 C786S), we also included a mutant with a deletion of the MIT domain (ΔMIT), which we previously showed is required for CHMP interaction and recruitment to endosomes (Row et al., 2007). Our results show that only wild-type, catalytically active, and endosome-associated USP8 is able to restore CTLA4 downregulation (Fig. 8, C and D). In parallel, we monitored CTLA4 ubiquitylation status, which was likewise rescued by wild-type but not catalytically inactive or MIT-deleted mutants of USP8 (Fig. 8, E and F).

### Ubiquitylation at Lys 203 and 213 promotes CTLA4 degradation

**Figure 9.**
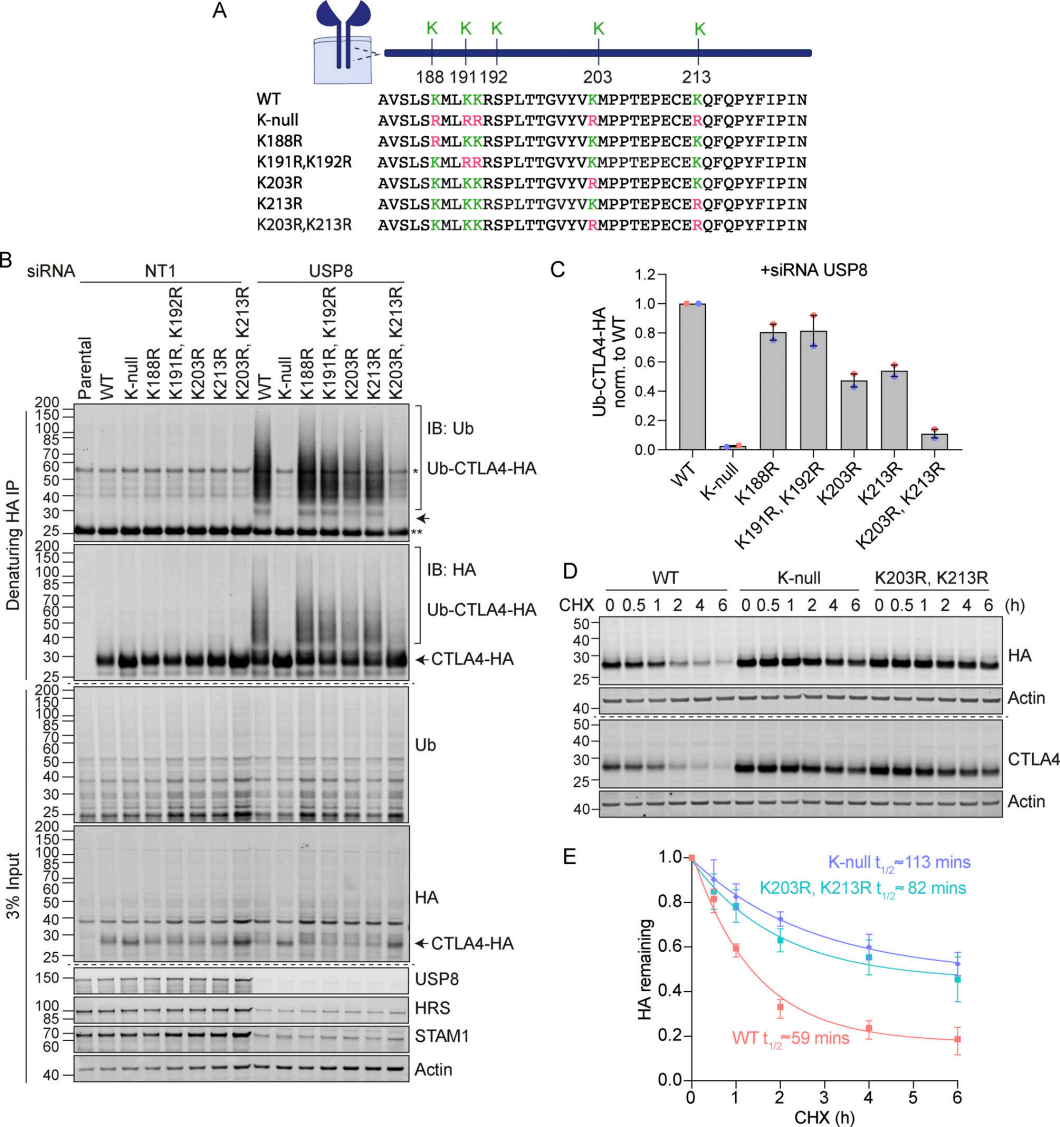
**Ubiquitylation of CTLA4 on Lys203 and Lys213 is responsible for its rapid turnover. (A)** Depiction of the cytoplasmic tail of CTLA4 and the lysine mutants analyzed in this study. **(B)** Representative western blots of ubiquitylated CTLA4-HA immunoprecipitated under denaturing conditions. HeLa S3 Flp-In parental (Par) or CTLA4-HA WT or lysine mutant cells were transfected for 72 h with non-targeting (NT1) or USP8 siRNA. Cells were lysed in denaturing SDS lysis buffer and lysates were subjected to immunoprecipitation (IP) with anti-HA coupled magnetic beads. *Antibody heavy chain; ** antibody light chain. IB: Immunoblot. **(C)** Quantification of ubiquitylated lysine mutant CTLA4-HA relative to WT for data represented in B. Ubiquitylated CTLA4-HA is shown normalized to immunoprecipitated CTLA4-HA. Individual data points from two independent, color-coded experiments are shown. Error bars indicate the range. **(D)** Representative western blots of HeLa S3 Flp-In CTLA4-HA WT, K-null, and K203R,K213R double lysine mutant cells treated with CHX for indicated times before lysis. **(E)** Quantification of CTLA4-HA turnover for data represented in D. The half-life of CTLA4-HA was estimated using an exponential decay model. Error bars indicate SD from three independent experiments. Source data are available for this figure: SourceData F9.

**Figure S4.**
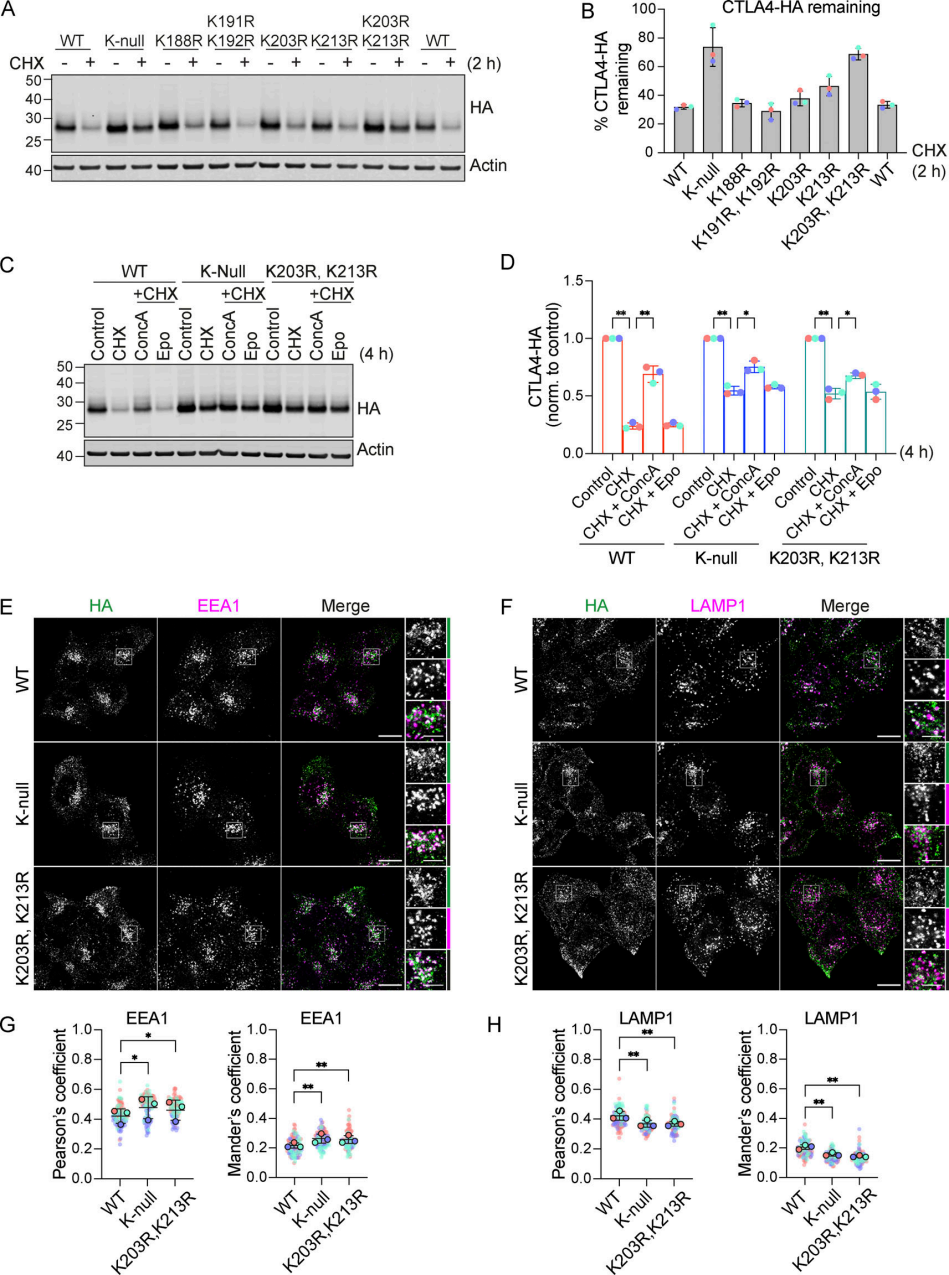
**Mutation of K203 and K213 delays CTLA4 turnover. (A)** Representative western blots showing increased stability of CTLA4-HA K-null and K203R, K213R mutants. HeLa S3 Flp-In CTLA4-HA WT and indicated lysine mutants were treated with Cycloheximide (CHX) for 2 h before lysis. **(B)** Quantification of CTLA4-HA remaining following CHX treatment. Individual data points from three independent, color-coded experiments are shown. Error bars show SD. **(C)** Representative western blots of HeLa S3 Flp-In CTLA4-HA WT, K-null and K203R, K213R double lysine mutants treated for 4 h with Cycloheximide (CHX) alone or together with Concanamycin A (ConcA) or Epoxomicin (Epo) prior to lysis. **(D)** Quantification of CTLA4-HA remaining normalised to control treated cells for data represented in C. Individual data points from three independent, color-coded experiments are shown. Error bars show SD. Two-way ANOVA multiple comparisons with uncorrected Fisher’s LSD, *P < 0.05, **P < 0.01. **(E and F)** Representative confocal images of CTLA4-HA WT, K-null, and K203R,K213R mutants co-stained with EEA1 (E) or LAMP1 (F). Scale bar = 15 µm (main figure) and 5 µm (inset). **(G and H)** Co-localization analysis of CTLA4-HA WT, K-null and K203R,K213R, and with EEA1 (G) and LAMP1 (H). Graphs show Pearson’s coefficients or Mander’s coefficients. Error bars indicate SD for three independent, color-coded experiments. Opaque circles with dark outlines correspond to the mean value from each experiment. One-way ANOVA with Dunnett’s multiple comparisons test, *P < 0.05, **P < 0.01. Source data are available for this figure: SourceData FS4.

CTLA4 harbors five lysines in its cytoplasmic tail (Fig. 9 A). We set out to identify which of these are critical for its ubiquitylation by generating a series of single and double point mutants as well as a “K-null” mutant in which all five lysines are converted to arginine. Replacing all lysines eliminated CTLA4 ubiquitylation, whilst mutating the three amino acids most proximal to the transmembrane domain had only negligible effects (Fig. 9, B and C). Mutation of the last two lysines in the C-terminal tail together (K203R, K213R), all but abolished ubiquitylation, whilst each individual mutation reduced the signal by half. Importantly, mutation of these same lysines dramatically increases the stability of CTLA4 to a similar degree as mutation of all lysines (K-null) or ConcA treatment (Fig. 9, D and E; and Fig. S4, A–D). In line with the slower turnover, the steady-state distribution of both K-null and K203R, K213R mutants is partially shifted from late (LAMP1-positive) to early (EEA1-positive) endosomal compartments (Fig. S4, E–H).

### UbiCRest analysis of CTLA4-associated polyubiquitin chains

**Figure 10.**
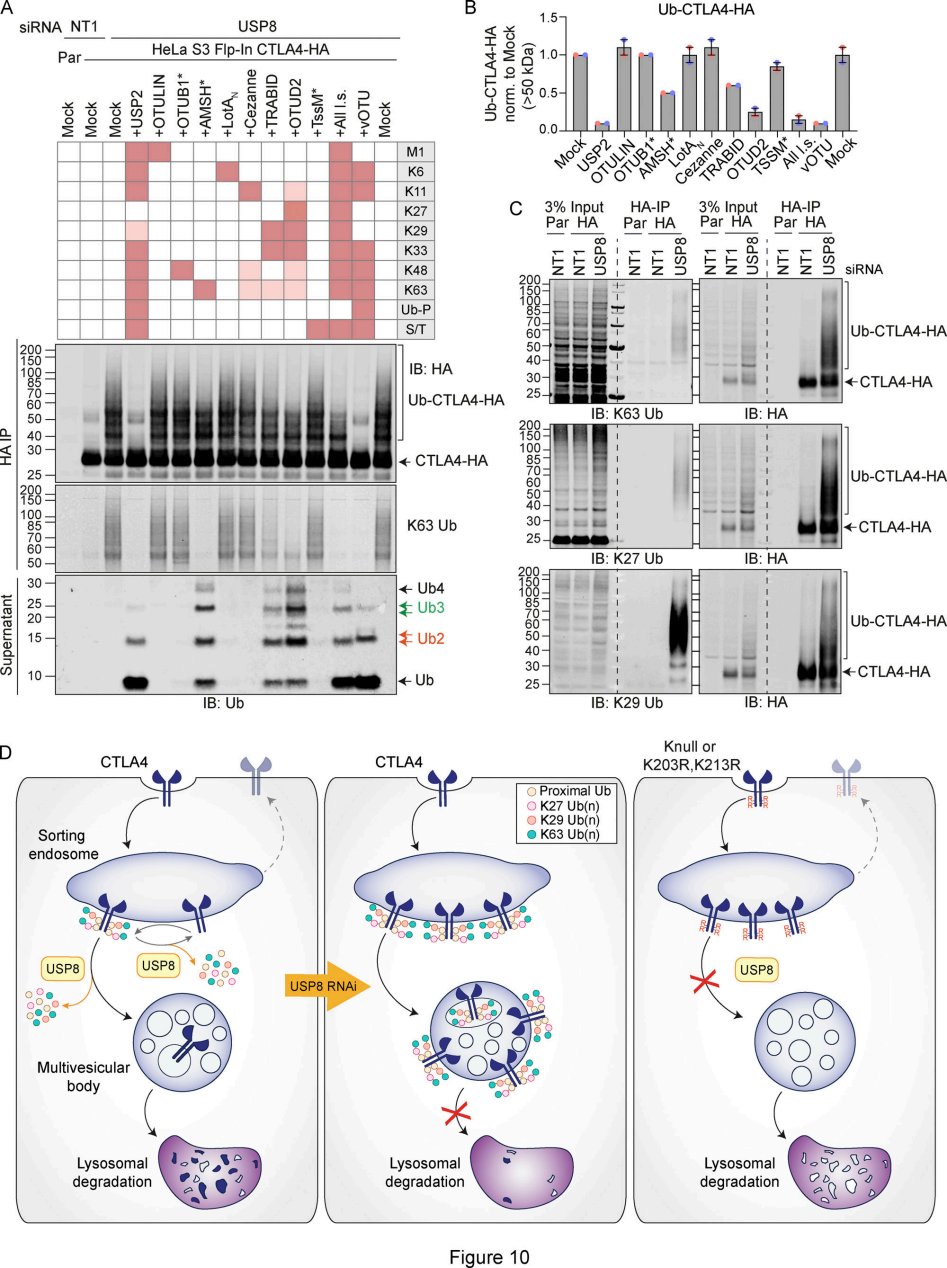
**Ubiquitin chain restriction (UbiCRest) analysis reveals K63, K27, and K29 ubiquitin chain association with CTLA4-HA. (A)** Representative UbiCRest analysis of western blots for CTLA4-HA. HeLa S3 Flp-In CTLA4-HA were transfected for 72 h with non-targeting (NT1) or USP8 siRNA prior to lysis and HA-immunoprecipitation (IP; IB: Immunoblot). CTLA4-HA-beads were treated with indicated DUBs or buffer only (Mock) for 1 h at 37°C, and analyzed alongside supernatants (released ubiquitin species). Specific activities of DUBs as reported in the literature are depicted in the key above the blot. Ub-P indicates the ability to cleave proximal ubiquitin; S/T denotes activity for serine or threonine linkages. All l.s.: All linkage-specific DUBs (OTULIN, OTUB1*, AMSH*, LotA_N_, Cezanne, TRABID, OTUD2, TssM*). **(B)** Quantification of Ub-CTLA4-HA signal remaining after DUB-treatment shown relative to Mock (“buffer only” control) for data represented in A. Individual data points from two independent, color-coded experiments are shown. Error bars indicate the range. **(C)** Representative western blots of CTLA4-HA immunoprecipitated (IP) under denaturing conditions were probed with K63 (top), K27 (middle), and K29 (bottom) ubiquitin chain linkage-specific antibodies. **(D)** Left Panel: A balance of E3 ligase and deubiquitylating activity governs receptor fate at the endosome. Endosomal USP8 is proposed to recycle ubiquitin from CTLA4 prior as well as after commitment to the lysosomal degradation pathway. Middle Panel: In the absence of USP8, a complex pattern of ubiquitylation accrues, composed of conventional Lys63 and unusual Lys27 and Lys29 ubiquitin linkages. This would normally lead to rapid degradation but is countermanded by downstream effects of USP8 loss that inhibit delivery to a degradative lysosome. That some ubiquitylated CTLA4 reaches the internal vesicles of the multivesicular body can be inferred from its presence in exosomes released upon v-ATPase inhibition. Right panel: Mutation of all lysines (K-null) or just the critical two lysines (K203 and K213) to arginines in the cytoplasmic tail of CTLA4 interferes with endolysosomal sorting and downregulation. These CTLA4 mutants accumulate in early (sorting) endosomes and fail to progress to later endolysosomal compartments. For reasons of clarity, mono-ubiquitylation, branching, and diversity of chain length are not depicted. Source data are available for this figure: SourceData F10.

**Figure S5.**
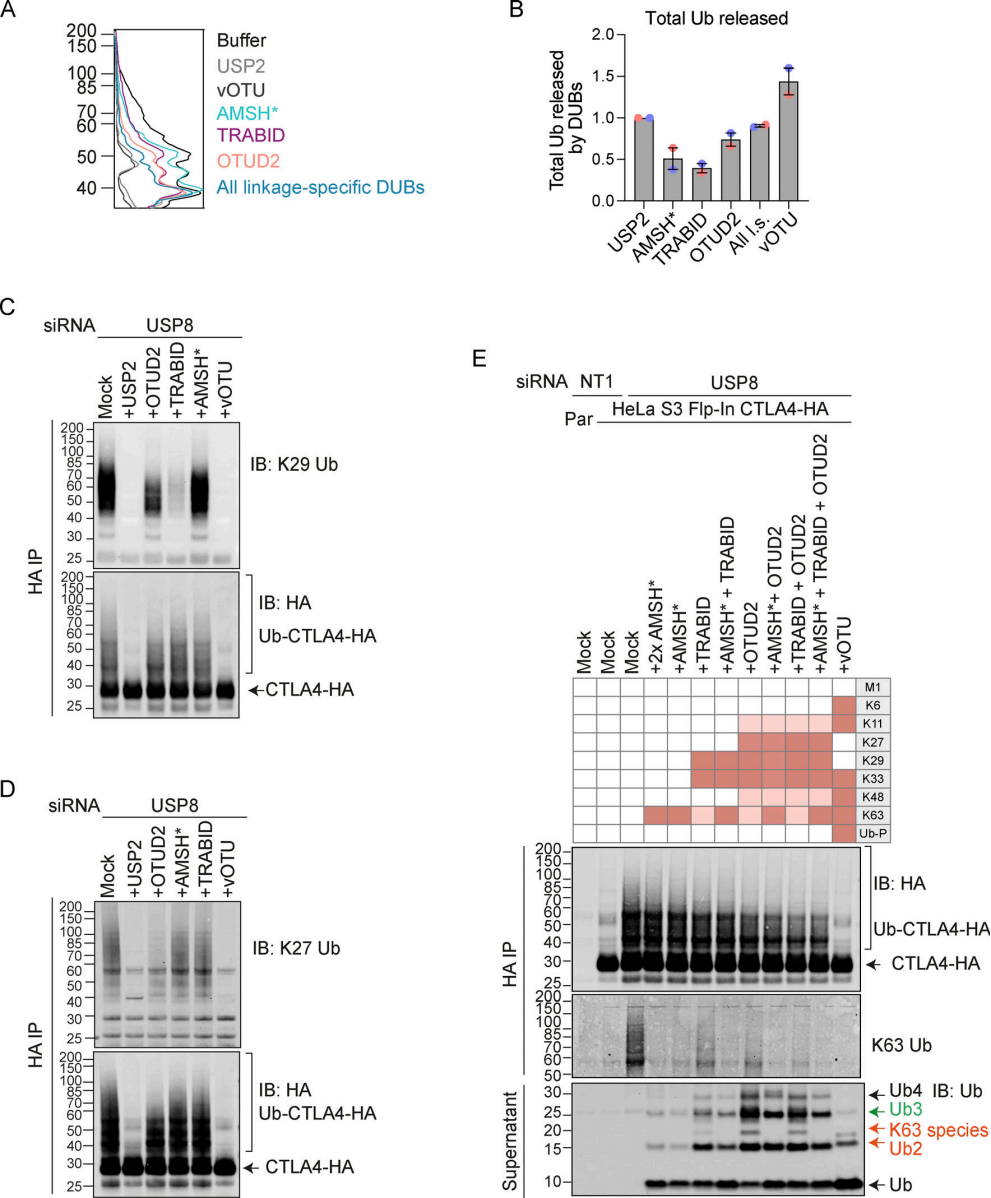
**Combinatorial UbiCRest analysis of CTLA4-HA. (A)** Line graphs corresponding to the Ub-CTLA4-HA signal shown in Fig. 10 A. **(B)** Quantification of total ubiquitin released into the supernatant by DUBs relative to USP2 for data represented in Fig. 10 A. Individual data points from two independent, color-coded experiments are shown. Error bars indicate the range. **(C and D)** Representative western blot showing K29- (C) and K27-linked (D) ubiquitin associated with immunoisolated CTLA4-HA after UbiCRest treatment with indicated DUBs. HeLa S3 Flp-In CTLA4-HA cells were transfected for 72 h with USP8 siRNA prior to lysis. CTLA4-HA was immunoprecipitated using anti-HA magnetic beads and incubated for 1 h at 37°C with the indicated DUBs. **(E)** Representative western blot of UbiCRest analysis of CTLA4 using a combination of linkage-specific DUBs. HeLa S3 Flp-In CTLA4-HA cells were transfected for 72 h with NT1 or USP8 siRNA prior to lysis. CTLA4-HA was immunoprecipitated using anti-HA magnetic beads and incubated for 1 h at 37°C with the indicated DUBs. Supernatants containing ubiquitin species released by DUBs were collected and analyzed in parallel with CTLA4-HA eluted from the beads. Specific activities of DUBs as reported in the literature are depicted in the key above the blot. Ub-P indicates ability to cleave proximal ubiquitin. Source data are available for this figure: SourceData FS5.

We next set out to identify the ubiquitin chain linkages associated with CTLA4-HA by performing a ubiquitin chain restriction (UbiCRest) analysis. This assay leverages the chain linkage specificity of various DUBs to infer the linkage composition of ubiquitylated species associated with a protein of interest (Hospenthal et al., 2015). USP2 and vOTU show high promiscuity toward ubiquitin chain linkages and will also remove any proximal ubiquitin moieties (Ub-P). In addition to a high molecular weight smear, a prominent discrete band at ∼40 kD, consistent with a dual mono-ubiquitylated form (Lys203, Lys213), was lost upon treatment with either enzyme (Fig. 10, A and B; and Fig. S5 A). Of all the linkage-specific DUBs tested, only AMSH*, TRABID, and OTUD2 removed ubiquitin chains from CTLA4-HA, which is best appreciated by focusing on the smear of protein above 60 kDa and the release of ubiquitin species into the supernatant (Fig. 10, A and B; and Fig. S5, A and B). The ubiquitylation signal was clearly reduced upon treatment with the stringent Lys63 chain-directed enzyme AMSH* (Komander et al., 2009; McCullough et al., 2006). Direct conjugation of Lys63-linked chains to CTLA4 was also confirmed following immunoprecipitation under denaturing conditions and blotting with a chain-specific antibody (Fig. 10 C). OTUD2 was the most effective DUB for CTLA4-HA deubiquitylation but in distinction to AMSH* only effected partial loss of Lys63-linked ubiquitylation (Fig. 10, A and B; and Fig. S5, A and B). Whilst Lys63 linkages have been prominently linked to endosomal trafficking, we reasoned that other linkage types must be present on CTLA4-HA (Clague et al., 2012). TRABID and OTUD2, which also reduced CTLA4 ubiquitylation, can both hydrolyze Lys29 and Lys33 ubiquitin chains, but only OTUD2 can remove Lys27 ubiquitin chains (Licchesi et al., 2011; Mevissen et al., 2013; Michel et al., 2015). More processing is evident when comparing OTUD2 with TRABID indicating the likely presence of Lys27 chains. By immunoprecipitating CTLA4-HA from denatured lysates, we could confirm both Lys27 and Lys29-ubiquitin chain linkages using a Lys27-chain specific antibody and a synthetic Lys29-ubiquitin antigen binding fragment respectively (Fig. 10 C). The Lys29 signal was removed very efficiently by TRABID but was only partially sensitive to OTUD2 and resistant to AMSH*, whereas the Lys27 signal was untouched by either AMSH* or TRABID but reduced significantly by OTUD2 (Fig. S5, C and D). We next analyzed the ubiquitin that is released in the UbiCRest assay into the supernatant (from CTLA4 and associated proteins that specifically co-immunoprecipitate). We could see that this is incompletely processed, leaving residual oligomers indicative of heterotypic and possibly branched chain ubiquitin species (Fig. 10 A and Fig. S5 E). For example, treatment with OTUD2 generates a higher molecular weight species, which is digested by co-incubation with AMSH* (Fig. S5 E, K63-species). The composite picture that emerges is a complex mixture of monoubiquitin and Lys63 alongside more unusual Lys27 and Lys29 chain types (Fig. 10 D).

## Discussion

There is an incongruity between the prominence of CTLA4 within the immuno-therapeutic space and our rudimentary understanding of its dynamics and trafficking itinerary. Pioneering work from the Sansom laboratory has shown that exogenous expression of CTLA4 in epithelial-derived cells can broadly capture its behavior in T cells (Janman et al., 2021; Qureshi et al., 2012; Rowshanravan et al., 2018). Here, we have adopted a similar approach of exogenous expression, whilst using a Flp-In cell line, to create isogenic cell panels that allow for highly accurate comparison between mutants. In parallel, we have also utilized melanoma and squamous lung carcinoma cell lines that naturally express CTLA4. In all cases, cycloheximide chase experiments confirm the short half-life previously reported, which is significantly <1 h in our hands (Qureshi et al., 2012). This is highly unusual, as only ∼5% of the proteome has a half-life of <8 h (Li et al., 2021; Rusilowicz-Jones et al., 2022).

The principal degradation pathways are normally discriminated by the application of v-ATPase inhibitors (for lysosomal degradation) or proteasome inhibitors (Rusilowicz-Jones et al., 2022). Here, we show that the major CTLA4 turnover pathway is lysosomal, with no evidence of a proteasomal contribution despite the rapid turnover. The USP8-sensitive component of this pathway is also sensitive to the upstream loss of the CCV adaptor protein AP2. Thus, CTLA4 must reach the plasma membrane before being rapidly internalized for degradation. Scope remains for minor alternate pathways, including secretion in the form of exosomes demonstrated herein. Although ubiquitylation of CTLA4 has been previously noted, its significance has not been directly tested (Kennedy et al., 2022). Here, using acute application of the ubiquitin E1 enzyme inhibitor TAK-243, we show that active ubiquitylation is absolutely required for CTLA4 degradation. Within the cytoplasmic tail of CTLA4, we have been able to show that direct ubiquitylation of just two out of the five candidate Lys residues governs stability. This opens up a new vista for identifying druggable targets within the ubiquitin system, which might regulate CTLA4 expression. For example, regulatory DUBs have been established as tractable targets for selective small molecule inhibition (Clancy et al., 2021; Harrigan et al., 2018; Turnbull et al., 2017).

At this point, we have taken a candidate-based approach, focusing on the two DUB enzymes that are most prominent at endosomes: the Lys63-chain specific metalloenzyme AMSH and the non-selective USP cysteine protease family member USP8 (Clague et al., 2019; Urbé et al., 2012). Lys63-linked ubiquitin chain modifications have been shown to be critical for efficient lysosome-directed sorting of multiple receptor types, although the effects of AMSH loss tend to be modest (Barriere et al., 2007; Clague et al., 2012). We find no effect of AMSH depletion on CTLA4 stability. In contrast, USP8 depletion enhances both CTLA4 half-life and ubiquitylation. If such ubiquitylation is the critical signal for lysosomal sorting, one might expect its accrual to lead to enhanced degradation. However, in this specific context, this positive signal can be overridden if downstream elements of the degradation pathway are also responsive to USP8 loss, as previously observed for MET, EGFR, and CXCR4 receptor degradation (Fig. 10 D) (Berlin et al., 2010; Bowers et al., 2006; Row et al., 2006; Savio et al., 2016). The effects of USP8 loss on endosomes are highly pleiotropic and include the clustering of endosomes as well as the loss of the ESCRT-0 components and ubiquitin-binding proteins, HRS and STAM (Clague and Urbé, 2017). The block to degradation of CTLA4 cannot be accounted for by loss of ESCRT-0 alone, as depletion of HRS has no effect. Two lines of argument show that USP8 must associate with endosomes to exert this effect. Depletion of a key adapter protein critical for USP8 localization, HD-PTP, phenocopies the block to CTLA4. Secondly, the deletion of the MIT domain from USP8, which is required for localization, renders it incapable of rescuing the depleted phenotype.

Although our focus has been on CTLA4 expressed in cancer cell lines, we have also shown that our findings with respect to USP8 and CTLA4 translate to T cells. CTLA4 is constitutively expressed by FoxP3^+^ regulatory T cells (T_Regs_) and upregulated upon activation in conventional T cells. Mice with a T cell-specific inactivation of USP8 (Usp8f/fCd4Cre) develop colitis, which is mediated by CD8^+^ γδT cells in concert with dysfunctional T_Regs_ (Dufner et al., 2015). Colitis is also among the most frequent and problematic immune-mediated adverse events that are associated with dual checkpoint inhibition and a combination of anti-CTLA4 and anti-PD-1 monoclonal antibodies has been shown to exacerbate dextran sulfate sodium-induced autoimmune colitis in mice (Perez-Ruiz et al., 2019; Postow et al., 2018). In this setting, it is difficult to tease out CTLA4-specific effects on T cell receptor signaling from other indirect effects. For example, USP8 is known to bind to the T cell receptor adaptor protein, Gads, and influence Foxo1 binding to chromatin (Dufner et al., 2015). Notwithstanding these caveats, we speculate that the disequilibrium imposed on CTLA4 following USP8 deletion may contribute to the colitis phenotype (Dufner et al., 2015).

The lack of impact of AMSH upon CTLA4 may reflect the involvement of chain types other than its exclusive substrate, Lys63-linked chains, that have been most prominently linked to endocytosis (Clague et al., 2012). We believe this is why USP8 but not AMSH regulates ESCRT-0 stability, despite their sharing of many interactions (Clague et al., 2012; Clague and Urbé, 2006). AMSH will not cleave a proximal ubiquitin and we here provide evidence for a significant population of dual mono-ubiquitylated CTLA4, which provides a relatively weak endosomal sorting signal for other receptor types (Haglund et al., 2003; Ma et al., 2012). Our UbiCRest chain analysis comes with the caveat that we are analyzing modifications that accrue in the absence of USP8, for otherwise the ubiquitylation is too labile for such analysis. As USP8 is a relatively promiscuous enzyme for different chain linkages, the most parsimonious interpretation is that the palette we reveal reflects that which may be occurring in unperturbed cells. If anything, a slight bias against Lys27 and Lys29 linkages is predicted because these are not particularly good USP8 substrates, at least in their diubiquitin form (Ritorto et al., 2014; van Tol et al., 2023). We provide evidence for a heterogeneous, partially branched, population that includes poorly characterized linkages not previously directly associated with receptor trafficking. However, two ESCRT machinery-linked proteins have been shown to have Lys27/29 (STAM) or Lys29/48 (VPS34) ubiquitin modifications (Chen et al., 2021; McElrath et al., 2023). The Parkinson’s disease-associated kinase LRRK2, which has a role in endolysosomal stress responses, can also be regulated by K27 and K29-linked ubiquitin chain modifications (Nucifora et al., 2016).

Heterotypic branched chains have been shown to enhance the rate of proteasomal degradation of cytosolic substrates (Meyer and Rape, 2014). We propose that a similar principle holds true for lysosomal sorting of short-lived membrane proteins such as CTLA4, which we show presents a complex branched ubiquitin chain profile composed of Lys63, Lys27, and Lys29 linkages. The present study opens up the ubiquitin system as a key player in CTLA4 biology that likely includes specific druggable regulators. So far biological agents such as antibodies have been used to effect changes in CTLA4 function for clinical purposes. We envision that future developments of small molecule regulators of CTLA4 stability may provide a complementary approach.

## Materials and methods

### Cell culture

A2058 cells were cultured in DMEM supplemented with GlutaMAX and 10% FBS; HeLa S3 Flp-In cells in DMEM supplemented with GlutaMAX, 10% FBS, and 1× non-essential amino acid (NEAA); NCI-H520 cells in RPMI supplemented with GlutaMAX and 10% FBS at 37°C and 5% CO_2_. Cells were routinely screened for mycoplasma infection.

### Generation of HeLa S3 Flp-In CTLA4-HA and CTLA4-HA lysine mutants

Human CTLA4-HA DNA flanked by BglII/EcoRI sites was synthesized and provided in a pTWIST-High-Kan vector by TWIST Bioscience, and subcloned into pcDNA3.1 and pEF5/FRT plasmids. For the generation of the CTLA4-HA lysine mutants (K-null; K188R; K191R/K192R; K203R; K213R; K203R/K213R), gene fragments flanked by BamHI/NotI sites, encoding the cytoplasmic tail of CTLA4 encompassing the relevant mutations (132–223) and a C-terminal HA tag, were synthesized by TWIST, subcloned into pEF5/FRT/CTLA4-HA and sequence-verified. To generate stable cell lines expressing wild-type and CTLA4-HA lysine mutants, HeLa S3 Flp-In host cells were co-transfected with pEF5/FRT/CTLA4-HA and pOG44 at a ratio of 1:9 using GeneJuice (70967; Merck Millipore) according to manufacturer’s instructions. Transfected cells were selected using Hygromycin B (10687010, 150 µg/ml; Invitrogen). For WT CTLA4, individual clones were amplified and the lysine mutant-expressing cells were maintained as pools.

### Mouse T cell isolation, expansion, and USP8 deletion

T cells were isolated from spleens of sex-matched 8–12-wk-old Rosa26-CreERT2 (Gt[ROSA]26Sortm2[cre/ERT2]Brn) USP8 fl/fl mice and USP8fl/fl (USP8^tm1floxedKPK^) litter mate controls (Hameyer et al., 2007; Niendorf et al., 2007) using BD IMag Biotinylated Mouse CD4 T lymphocyte enrichment Cocktail and BD IMAG Streptavidin Particles Plus according to manufacturer’s instructions. Ex vivo expansion of T cells was performed using anti-CD3 (clone 145-2C11, 1 µg/ml; eBioscience) and anti-CD28 antibodies (clone 37.51, 1 µg/ml; eBioscience) on TC plates precoated with anti-hamster antibody (G94-56; BD Pharmingen), and IL-2 (100 U/ml; Immunotools) for continued cell culture. 4-Hydroxytamoxifen (OHT, 1 µM; Sigma-Aldrich) was added 24 h post T cell isolation and stimulation. Cells were lysed 72 h post-OHT addition.

### Transfection and siRNA interference

Cells were treated with 40 nM non-targeting (NT1) or target-specific siRNA oligonucleotides (Dharmacon) using Lipofectamine RNAi-MAX (13778030; Invitrogen) according to manufacturer’s instructions. The medium was exchanged 24 h after transfection and cells were harvested 72 h posttransfection. To deplete AP2, a double-hit transfection protocol using Lipofectamine RNAiMAX (13778030; Invitrogen) was employed. Cells were treated with 40 nM AP2 siRNA and re-seeded 24 h posttransfection for a second round of transfection using 40 nM AP2 siRNA. The medium was exchanged 24 h after the second transfection and cells were harvested 72 h later. For plasmid transfection, GeneJuice (70967; Merck Millipore) was used according to the manufacturer’s instructions. For rescue experiments, endogenous USP8 was depleted by transfecting cells with USP8 oligo 1 (40 nM) using RNAiMAX for 72 h, followed by GFP or GFP-USP8 siRNA-resistant constructs using GeneJuice for the last 46 h prior to lysis or fixation.

### siRNA and plasmids

All siRNA oligonucleotides were obtained from Dharmacon, Horizon Discovery: CTLA4 (SMARTPool, L-016267-00-0005: 5′-GAA​GCC​CUC​UUA​CAA​CAG​G-3′, 5′-GAA​CCC​AGA​UUU​AUG​UAA​U-3′, 5′-GUA​UGC​AUC​UCC​AGG​CAA​A-3′, 5′-GGA​CUG​AGG​GCC​AUG​GAC​A-3′), USP8 oligo 1 (siGNEOME, D-005203-02: 5′-UGA​AAU​ACG​UGA​CUG​UUU​AUU-3′, [Row et al., 2006]), custom-made AMSH oligo 2 (CTM-931180: 5′-UUA​CAA​AUC​UGC​UGU​CAU​UUU-3′, [McCullough et al., 2004]), HD-PTP (SMARTPool, L-009417-00-0005: 5′-GUG​CAC​AGG​UGG​UAG​AUU​A-3′, 5′-GCA​AAC​AGC​GGA​UGA​GCA​A-3′, 5′-GCA​UGA​AGG​UCU​CCU​GUA​C-3′, 5′-GUA​GUG​UCC​UCC​GCA​AGU​A-3′), HRS (SMARTPool, L-016835-00-0005: 5′-GAG​GUA​AAC​GUC​CGU​AAC​A-3′; 5′-GCA​CGU​CUU​UCC​AGA​AUU​C-3′; 5′-AAA​GAA​CUG​UGG​CCA​GAC​A-3′; 5′-GAA​CCC​ACA​CGU​CGC​CUU​G-3′), Syntenin (SMARTPool, L-008270-00-0005: 5′-GGA​GAG​AAG​AUU​ACC​AUG​A-3′, 5′-GAC​CAA​GUA​CUU​CAG​AUC​A-3′, 5′-GGA​UGG​UCU​UAG​AAU​AUU​U-3′, 5′-GCA​UUU​GAC​UCU​UAA​GAU​U-3′), ALIX (SMARTPool, L-004233-00-0005: 5′-CAG​AUC​UGC​UUG​ACA​UUU​A-3′; 5′-UCG​AGA​CGC​UCC​UGA​GAU​A-3′; 5′-GCG​UAU​GGC​CAG​UAU​AAU​A-3′; 5′-GUA​CCU​CAG​UCU​AUA​UUG​A-3′), custom-made AP2 oligo (CTM-1021281: 5′-AAG​UGG​AUG​CCU​UUC​GGG​UCA-3′, [Motley et al., 2003]), ATG7 (SMARTPool, L-020112-00-0005: 5′-CCA​ACA​CAC​UCG​AGU​CUU​U-3′, 5′-GAU​CUA​AAU​CUC​AAA​CUG​A-3′, 5′-GCC​CAC​AGA​UGG​AGU​AGC​A-3′, 5′-GCC​AGA​GGA​UUC​AAC​AUG​A-3′ and non-targeting 1 (NT1) control (ON-TARGETplus, D-001810-01, 5′-UGG​UUU​ACA​UGU​CGA​CUA​A-3′).

GFP-USP8* and GFP-∆MIT-USP8 have been previously described (Row et al., 2006, 2007). GFP-USP8*-C786S was generated by performing QuickChange site-directed mutagenesis using GFP-USP8* as the template and the following primer pairs: 5′-ACT​TAG​GAA​ATA​CTA​GTT​ATA​TGA​ACT​CA-3′ and 3′-TGA​GTT​CAT​ATA​ACT​AGT​ATT​TCC​TAA​GT-5′. Transformants were screened by restriction digestion and validated by sequencing.

### Antibodies and reagents

Antibodies and other reagents used were as follows: anti-human CTLA4 E1V6T (1:500, 96399, WB; Cell Signalling Technology), anti-mouse CTLA4 (1:1,000, AF476, WB; R&D Systems), anti-human CTLA4 BNI3 (1:100, 555851, IF; BD Pharmingen), anti-CTLA4 F8 (1:500, sc-376016, WB; Santa Cruz Biotechnology), anti-HA (1:1,000, MMS-101P, WB; Covance), anti-USP8 (1:500, AF7735, WB; R&D Systems), anti-USP8 (1:2,000, A302-929A, WB; Bethyl), anti-mouse USP8 X39 (a gift from Enzo Martegani, WB 1:2,000, [Gnesutta et al., 2001]), anti-AMSH (In house, rabbit 850, WB 1:1,000, [McCullough et al., 2004]), anti-HRS (In house, rabbit 864/3, WB 1:1,000, [Sachse et al., 2002]), anti-HRS (1:2,000, EB07211, WB; Everest Biotech), anti-HRS (1:1,000, ab155539, WB; Abcam, IF 1:200), anti-STAM1 (1:1,000, ab155527, WB; Abcam), anti-ubiquitin VU1 (1:2,000, VU101, WB; LifeSensor; Fig. 1, C and E), anti-ubiquitin U5379 (1:1,000, U5379, WB; Sigma-Aldrich; Fig. 10 A and Fig. S5 E), anti-ubiquitin FK2 (Enzo, PW8810, WB 1:1,000, Fig. 4, A, C, and E; Fig. 5, G and H; Fig. 7, B and D; Fig. 8 E; and Fig. 9 B), anti-ubiquitin Lys63-specific clone Apu3 (1:1,000, 05-1308, WB; Millipore), anti-ubiquitin Lys27-specific (1:1,000, 181537, WB; Abcam), anti-ubiquitin Lys29-synthethic antigen fragment (sAB, In house, WB 2 µg/ml [Yu et al., 2021]), anti-HD-PTP (1:500, 10472-1-AP, WB; ProteinTech), anti-ALIX (1:1,000, sc-53540, WB; Santa Cruz Biotechnology), anti-Syntenin 1 (1:1,000, H00006386-B01P, WB; Novus Biologicals), anti-TOMM20 (1:1,000, 612278, WB; BD Transduction), anti-CD63 (1:500, NBP2-42225SS, WB; Novus Biologicals), anti-HA (1:10,000, NB600-362, WB; Novus Biologicals, IF 1:250), sheep anti-GFP (gift from Ian Prior, University of Liverpool, Liverpool, UK, WB 1:1,000), anti-Actin (1:10,000, 66009, WB; Proteintech), anti-Actin (1:1,000, A2066, WB; Sigma-Aldrich), anti-γ-Tubulin (1:1,000, ab11317, WB; Abcam), anti-LAMP1 D2D11 (1:400, 9091, IF; Cell Signalling Technology), anti-EEA1 (1:500, 610456, IF; BD Transduction Laboratories), anti-EEA1 (In house, rabbit 243/3, IF 1:1,000), anti-AP2 (1:500, 611350, WB; BD Transduction), anti-ATG7 (1:1,000, 2,631, WB; Cell Signalling Technology), anti-mouse CTLA4 E5S7E (1:200, 50490, IF; Cell Signalling Technology); Cycloheximide (C7698, 100 µg/ml; Sigma-Aldrich), Concanamycin A (C9705, 100 nM; Sigma-Aldrich), Folimycin A (344085, 100 nM; Sigma-Aldrich), Epoxomicin (324800, 100 nM; Millipore), TAK-243 (S8341, 1 µM; Selleckchem), and Dynasore (S8047, 80 µM; Selleckchem).

### Purification of K29 synthetic antibody fragment (sAB)

The K29 sAB expression plasmid was a kind gift from Dr. Minglei Zhao (#204735; Addgene, [Yu et al., 2021]). Protein was expressed in BL21 *E. coli* at 37°C for 4 h following induction with 1 mM IPTG. Cells were harvested in 25 mM Tris (pH 7.4), 200 mM NaCl (Buffer B), and incubated with lysozyme and protease inhibitor cocktail (Millipore, Sigma-Aldrich), and then lysed by sonication. Clarified lysate was incubated at 65°C in a water bath for 30 min, cooled on ice, and the precipitated proteins were cleared by centrifugation. The resulting supernatant was loaded onto a HiTrap MabSelect VL column (Cytiva). The protein was washed with 5 column volumes (CVs) of Buffer B and eluted over a 10 CV 0–100% gradient toward 50 mM sodium citrate (pH 2.5). Fractions were collected into tubes containing 50 μl of 1 M Tris (pH 8.0) to neutralize the pH and were analyzed by SDS-PAGE. sAB-containing fractions were pooled and dialyzed into 25 mM sodium phosphate (pH 7.4), 150 mM NaCl prior to quantification by absorbance at 280 nm, and flash freezing for storage at −80°C.

### Cell lysis and western blot analysis

Cultured cells were washed twice in ice-cold PBS and lysed in NP40 lysis buffer (0.5% NP40, 25 mM Tris pH 7.5, 100 mM NaCl, and 50 mM NaF) supplemented with mammalian protease inhibitor cocktail (P8340; Sigma-Aldrich) and PhosSTOP (49068450001; Roche) for 10 min on ice. Lysates were clarified by centrifugation and protein concentration was determined using the Pierce BCA protein assay according to the manufacturer’s instructions. Samples were diluted with 5 × SDS-sample buffer (15% wt/vol SDS, 312.5 mM Tris-HCl pH 6.8, 50% glycerol and 16% β-mercaptoethanol) and boiled at 95°C. For non-reducing blots (CD63), samples were prepared in 3× non-reducing sample buffer (187.5 mM Tris-HCl pH 6.8, 30% wt/vol glycerol, 9% wt/vol SDS and 1.25% wt/vol Bromophenol blue) and heated at 60°C for 5 min. Proteins were resolved using SDS–PAGE (Invitrogen NuPage gel 4–12%), transferred to nitrocellulose membrane (10600001 or 10600002; Amersham Protran 0.2 and 0.45 µm pore size), stained with Ponceau S staining solution (P7170; Sigma-Aldrich), blocked in 5% milk (Marvel), or 0.5% fish skin gelatin (G7765; Sigma-Aldrich) in TBST (TBS: 20 mM Tris–Cl, pH 7.6 and 150 mM NaCl, supplemented with Tween-20 (10485733; Thermo Fisher Scientific) before incubation with primary antibodies overnight. Visualization and quantification of western blots were performed using IRdye 800CW (anti-mouse 926-32212, anti-rabbit 926-32213, and anti-goat 926-32214) and IRdye 680LT (anti-mouse 926-68022, anti-rabbit 92668023, and anti-goat 926-68024) coupled secondary antibodies and an Odyssey infrared scanner (LI-COR Biosciences). For western blot quantification, raw signal values were obtained using ImageStudio Lite (Li-COR) following background subtraction, and the raw values of each condition were normalized to the average of the quantified raw values from each individual blot. For stripping and reprobing, membranes were incubated in 2% SDS, 62.5 mM Tris-HCl pH 6.7, and 100 mM β-mercaptoethanol for 30 min in a 50°C water bath, then washed three times in 0.2% TX100 in PBS, followed by incubation in blocking buffer. To visualize Lys29-linked ubiquitin chains using sAB, peroxidase-conjugated goat anti-human IgG, F(ab)2 fragment specific (1:10,000, 109-036-006; Jackson ImmunoResearch) was applied as the secondary antibody. Probed membranes were then incubated with Clarity Western ECL substrate (170-5060; Bio-Rad) and imaged using a ChemiDoc Touch Imaging System (Bio-Rad).

### Immunoprecipitation

For co-immunoprecipitation experiments, HeLa S3 Flp-In CTLA4-HA cells were lysed in NP40 lysis buffer as described above. Clarified lysates were incubated with 25 μl of anti-HA magnetic beads (88837; Thermo Fisher Scientific) overnight at 4°C. Beads were washed three times with 0.05% TBST and proteins were eluted in sample buffer. For denaturing immunoprecipitation of CTLA4-HA, cells were washed twice with prewarmed PBS and lysed in denaturing SDS lysis buffer (2% wt/vol SDS, 1 mM EDTA and 50 mM NaF) at 110°C, followed by boiling for 10 min with intermittent vortexing. Lysates were diluted with 4 vol of dilution buffer (2.5% Triton X-100, 12.5 mM Tris pH 7.5, and 187.5 mM NaCl) before incubation with 25 μl of anti-HA magnetic beads (88837; Thermo Fisher Scientific) at 4°C overnight. Beads were washed with TX100-SDS wash buffer (2% Triton X-100, 0.4% SDS, 10 mM Tris pH 7.5, 150 mM NaCl) and proteins were eluted in 50 mM NaOH. 1M Tris pH 8.5 was added to the eluted samples to neutralize the pH and samples were prepared in 5 × “hot lysis” sample buffer (7% [wt/vol] SDS, 312.5 mM Tris-HCl pH 6.8, 50% [wt/vol] glycerol and 16% β-mercaptoethanol).

### Tandem ubiquitin binding entities (TUBES) pulldown

Cells were washed twice with ice-cold PBS and lysed in TUBES lysis buffer (50 mM Tris-HCl pH 7.5, 150 mM NaCl, 1 mM EDTA, 1% [wt/vol] NP40, 10% [wt/vol] glycerol) supplemented with mammalian protease inhibitor cocktail (P8340; Sigma-Aldrich), PhosSTOP (4906845001; Roche) and 10 mM NEM (E3876-5G; Sigma-Aldrich). Lysates were incubated with 20 μl (50% slurry) TUBES (UM402; Life sensors) or control agarose resin (UM400; Life sensors) overnight at 4°C. Beads were washed with 0.1% TBST and proteins were eluted in sample buffer at 95°C.

### TCA precipitation of proteins from media

Media were collected from 6-well plates and centrifuged at 3,000 *g* for 15 min to remove cell debris. SDS was added to the media at a final concentration of 0.02%, followed by incubation on ice for 30 min. Trichloroacetic acid (TCA, T0699; Sigma-Aldrich) was added to a final concentration of 10% for a further 1 h incubation on ice, followed by centrifugation at 16,200 *g* for 30 min at 4°C. The supernatant was removed and pellets were washed twice in ice-cold acetone. Pellets were air-dried and resuspended in SDS sample buffer before analysis by SDS-PAGE and western blot.

### Exosome enrichment by serial centrifugation

All centrifugation steps were carried out at 4°C. Media were collected from 6-well plates and centrifuged sequentially at 300 *g* for 20 min × 2 to remove cells, at 2,000 *g* for 20 min to remove large cell debris, at 10,000 *g* for 20 min to remove smaller cell debris (using a Himac Ultracentrifuge and a S55A2 rotor). The supernatants were collected and centrifuged at 100,000 *g* for 70 min using the same rotor to collect the exosomal fractions. The resulting pellets were washed in ice-cold PBS and re-pelleted again at 100,000 *g* for another 70 min using the same rotor. The exosome-enriched pellets were resuspended alongside the 10,000 *g* pellets in SDS-sample buffer and analyzed by SDS-PAGE and western blot.

### Proteinase K protection assay

To determine the topology of exosomal CTLA4, exosome-enriched pellets were resuspended in PBS containing 2.5 mM CaCl_2_ and 1 mM MgCl_2_ (PBS^++^). The samples were treated with proteinase K (P2308, 100 µg/ml; Merck) in 50 mM Tris-HCl pH 7.4 supplemented with 10 mM CaCl_2_ for 1 h at 37°C in the absence or presence of 1% (wt/vol) Triton X-100 in PBS^++^. To terminate the proteinase activity, phenylmethylsulfonyl fluoride (PMSF, 78830; Sigma-Aldrich, 2 mM final concentration) was added to the samples and incubated on ice for 5 min. The samples were prepared in sample buffer as described above.

### Protein expression and purification

*H. sapiens* OTUD2 (aa 1–348; pOPIN-K), *H. sapiens* OTULIN (aa 1–352; pOPIN-B), *H. sapiens* Cezanne (aa 129–438; pOPIN-E), *D. melanogaster* TRABID (aa 318–778; pOPIN-S), Crimean Congo Hemorrhagic Fever Virus vOTU (aa 1–183; pOPIN-K), and *L. pneumophila* LotA_N_ (aa 1–300; pOPIN-B) were prepared as described previously (Akutsu et al., 2011; Keusekotten et al., 2013; Mevissen et al., 2013; Warren et al., 2023; Xia et al., 2022, *Preprint*). OTUB1* and AMSH* are constitutively active engineered fusion proteins of *H. sapiens* UBE2D2(C85S)-OTUB1 and STAM-AMSH, expressed from the pOPIN-B vector as described previously (Michel et al., 2015). *B. pseudomallei* TssM* encodes the bacterial USP-type DUB engineered with a V466R mutation that enhances selectivity for ester- over isopeptide-linked ubiquitin, expressed from the pET-M30 vector as described (Szczesna et al., 2024). Briefly, all DUBs were expressed in *E. coli* Rosetta cells at 18°C for 16 h following induction with 0.2–0.5 mM IPTG. Cells were harvested in 25 mM Tris (pH 7.4), 200 mM NaCl, 2 mM β-mercaptoethanol (Buffer A), incubated with lysozyme and protease inhibitor cocktail (Millipore, Sigma-Aldrich), and lysed by sonication. Clarified lysate was applied to cobalt affinity resin (Thermo Fisher Scientific), washed with additional Buffer A, and eluted with Buffer A containing 250 mM imidazole. Eluates were dialyzed back into Buffer A overnight at 4°C prior to concentration with 10 kDa MWCO Amicon centrifugal filters (Millipore, Sigma-Aldrich), quantification by absorbance at 280 nm, and flash freezing for storage at −80°C. With the exception of OTUB1*, all His-tagged constructs expressed from pOPIN-B and pOPIN-E were left intact, while the His-GST and His-SUMO tags encoded by the pOPIN-K, pET-M30, and pOPIN-S constructs were cleaved by 3C, TEV, or SUMO proteases, respectively.

### Ubiquitin chain restriction (UbiCRest) assay

Cells were lysed in NP40 lysis buffer (0.5% NP40, 25 mM Tris pH 7.5, 100 mM NaCl and 50 mM NaF) supplemented with mammalian protease inhibitor cocktail (P8340; Sigma-Aldrich), PhosSTOP (49068450001; Roche), and 20 mM NEM (E3876-5G; Sigma-Aldrich). The lysates were incubated with anti-HA magnetic beads (88837; Thermo Fisher Scientific) overnight at 4°C to immunoprecipitate CTLA4-HA. The UbiCRest assay was performed in a 25 μl reaction volume at 37°C for 1 h in a thermoshaker at 900 rpm as previously described (Hospenthal et al., 2015). The DUBs used in this study are USP2 (BML-UW9850-0100, 1 µM; Enzo and E-504-0650, 1 µM; Boston Biochem), vOTU (1 µM), OTULIN (1 µM), OTUB1* (1 µM), AMSH* (1 µM), LotA_N_ (1 µM), Cezanne (0.2 µM), TRABID (0.2 µM), OTUD2 (1 µM), and TssM* (1 µM). The supernatants were collected after the incubation and prepared in a sample buffer to analyze ubiquitin released during the UbiCRest assay. Proteins were eluted from the anti-HA magnetic beads at 60°C in sample buffer.

### Immunofluorescence

HeLa CTLA4-HA and A2058 cells seeded on coverslips were either fixed with 4% paraformaldehyde (PFA, AGR1026; Agar Scientific) in PBS or ice-cold methanol. Excess PFA was quenched with 50 mM NH_4_Cl/PBS and cells were permeabilized with 0.2% Triton X-100 in PBS. To prepare T cells for immunofluorescence staining, cells were expanded and stimulated as described above with an additional phorbol myristate acetate (PMA, P1585, 50 ng/ml; Sigma-Aldrich) and Ionomycin (I0634, 1 µg/ml; Sigma-Aldrich) stimulation for 4 h before Cytospin deposition onto adhesive microscopic slides (T7613; Trajan) followed by 15 min fixation and permeabilization as described above. All fixed cells were incubated for 30 min in blocking solution (3% BSA in PBS or 10% goat serum in PBS), then stained with primary antibodies (1 h), followed by AlexaFluor-488- or AlexaFluor-594-coupled secondary antibodies (30 min) in blocking buffer. Coverslips were mounted onto glass slides using Mowiol containing DAPI. Cells were imaged using an LSM900 Airyscan confocal microscope (63× oil objective, acquisition software Zen Blue). All images were acquired sequentially and processed using Fiji (version 2.1.0) and Adobe Photoshop (version 24.5.0) software. Pearson’s and Mander’s coefficients (M2; the fraction of CTLA4(-HA) colocalizing with EEA1 or LAMP1 respectively) were measured using the JACoP plugin in Fiji (Bolte and Cordelières, 2006).

### Statistical analysis

Graphs were plotted using GraphPad Prism10. Statistical significance was determined using one-way ANOVA (Fig. 1, D and F; Fig. 5, B, C, E, and F; Fig. 8, D and F; Fig. S1 D; and Fig. S4, G and H), unpaired *t* test (Fig. 6, C, D, G, and H; and Fig. S2 D), or two-way ANOVA with uncorrected Fisher’s LSD (Fig. S4 D). P-values are represented as *P < 0.05, **P < 0.01, ***P < 0.001 and ****P < 0.0001.

### Online supplemental material

Fig. S1 shows the expression of CTLA4 in cancer cell lines and its sensitivity to Dynasore in a HeLa cell model. Fig. S2 shows CTLA4 secretion from Melanoma and HeLa cell lines via exosomes and its stability is not affected by depletion of ATG7. Fig. S3 shows the accumulation of CTLA4 in USP8-deleted mouse CD4 T cells and the impact of USP8 and HD-PTP depletion on exosomal CTLA4 secretion from melanoma cells. Fig. S4 shows that mutation of K203 and K213, or of all lysines delays CTLA4 turnover and promotes its accumulation in EEA1-positive early endosomes alongside de-enrichment in LAMP1-positive late endosomes. Fig. S5 shows a combinatorial UbiCRest analysis of the ubiquitin chain linkages associated with CTLA4-HA as well as a line graph and quantitation of western blot data shown in Fig. 10 A.

## Supplementary Material

SourceData F1 is the source file for Fig. 1.

SourceData F2 is the source file for Fig. 2.

SourceData F3 is the source file for Fig. 3.

SourceData F4 is the source file for Fig. 4.

SourceData F5 is the source file for Fig. 5.

SourceData F7 is the source file for Fig. 7.

SourceData F8 is the source file for Fig. 8.

SourceData F9 is the source file for Fig. 9.

SourceData F10 is the source file for Fig. 10.

SourceData FS1 is the source file for Fig. S1.

SourceData FS2 is the source file for Fig. S2.

SourceData FS3 is the source file for Fig. S3.

SourceData FS4 is the source file for Fig. S4.

SourceData FS5 is the source file for Fig. S5.

## Data Availability

Data are available in the article itself and its supplementary materials.
