# Supplementary figures and images for: Rapid turnover of CTLA4 is associated with a complex architecture of reversible ubiquitylation
Source: J Cell Biol. 2024 Oct 15;224(1):e202312141. doi: 10.1083/jcb.202312141 (PMC11486831; doi:10.1083/jcb.202312141)

Figure 1A

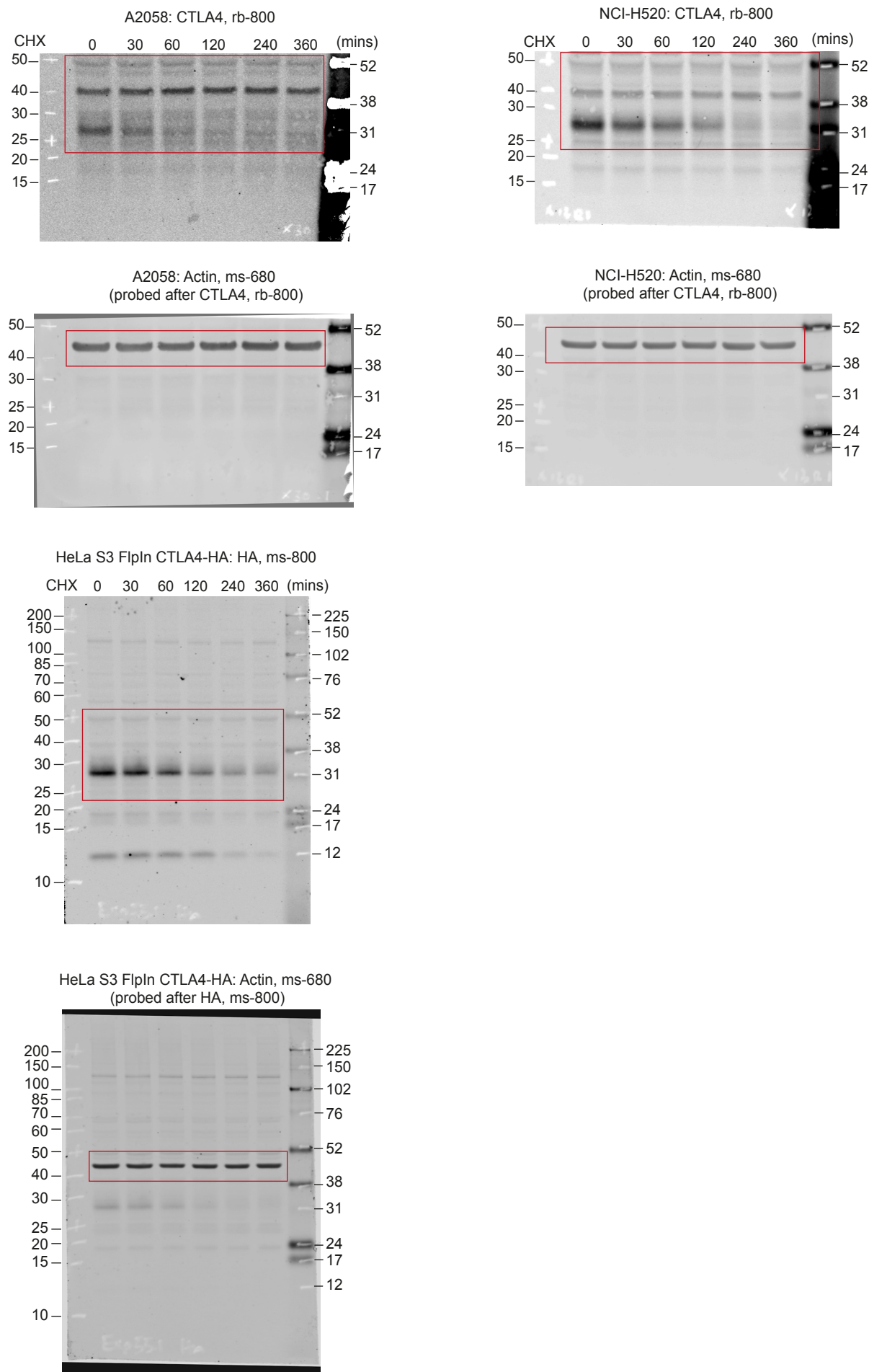

Figure 1C

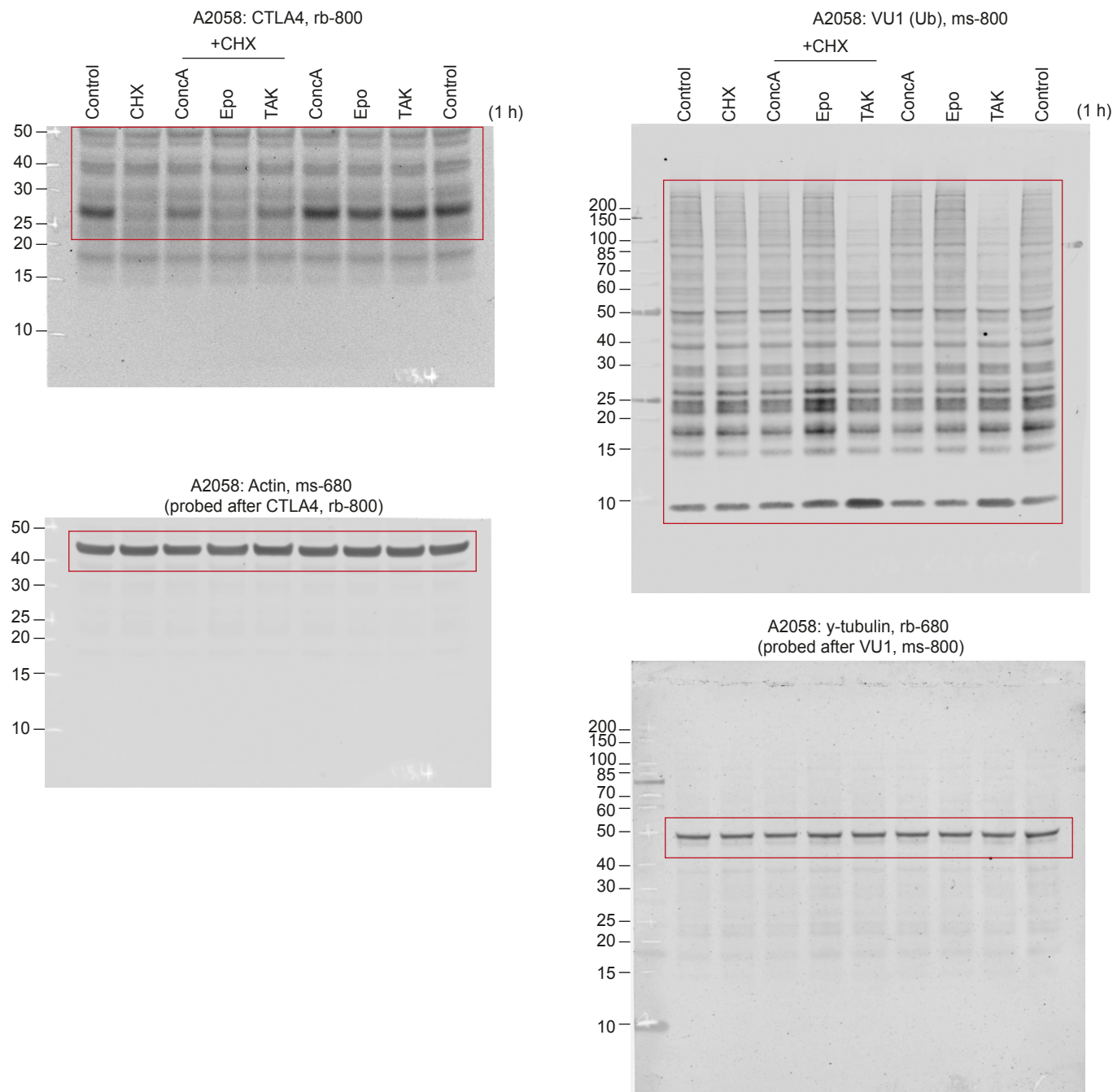

Figure 1E

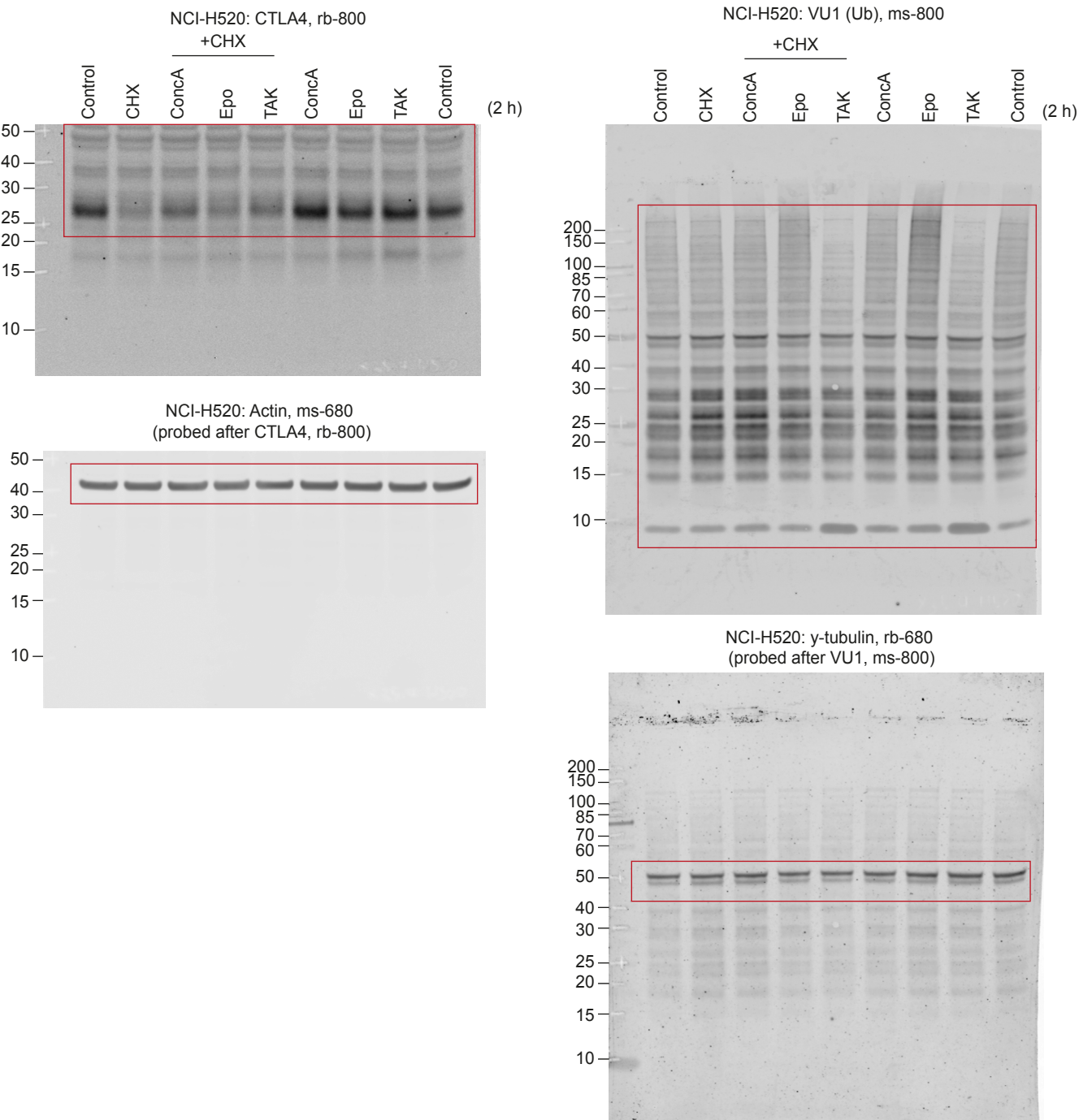

Supplement: SourceData F1 — is the source file for Fig. 1. [file JCB_202312141_SourceDataF1.pdf]

Figure 2A

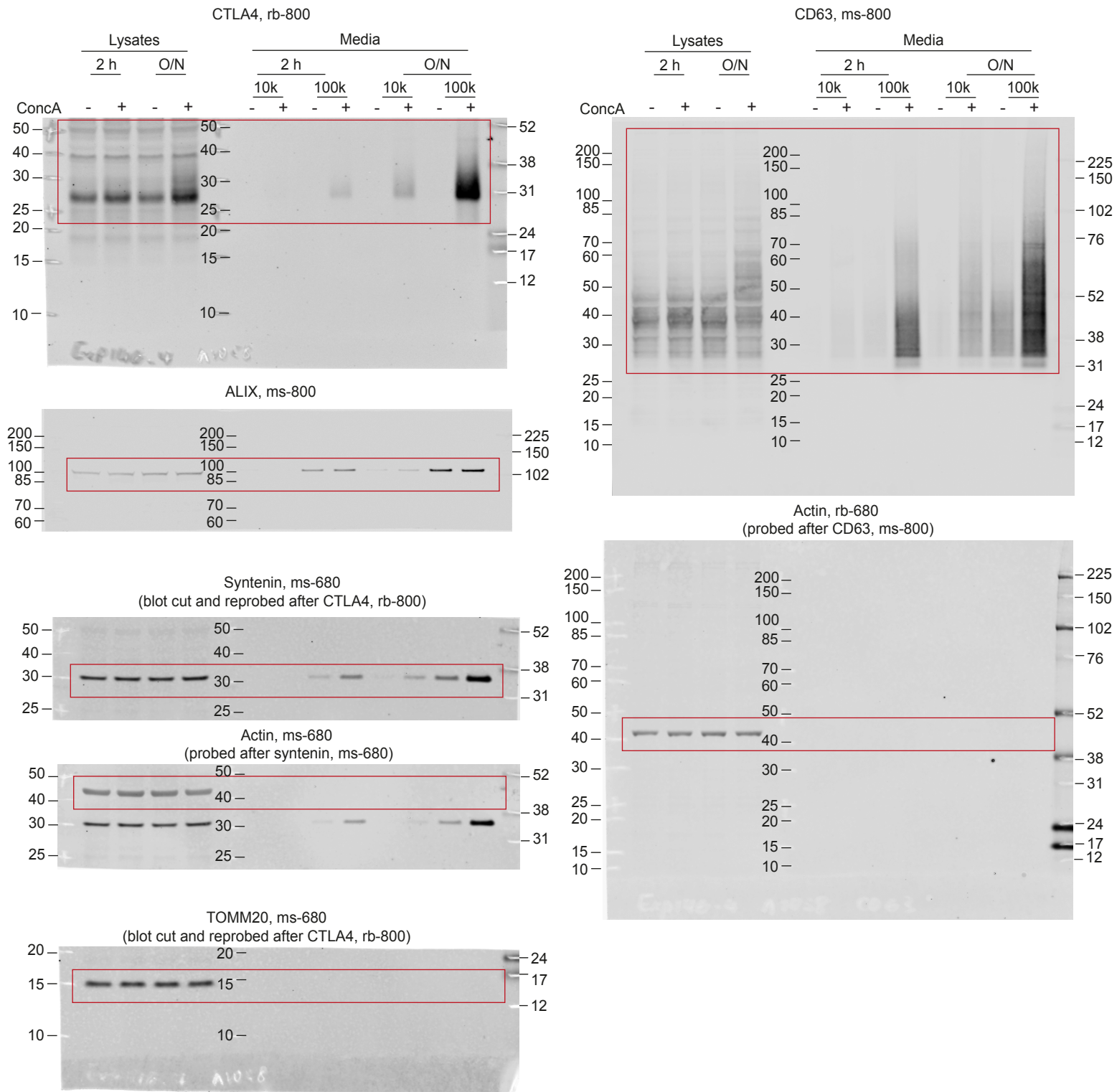

Figure 2D

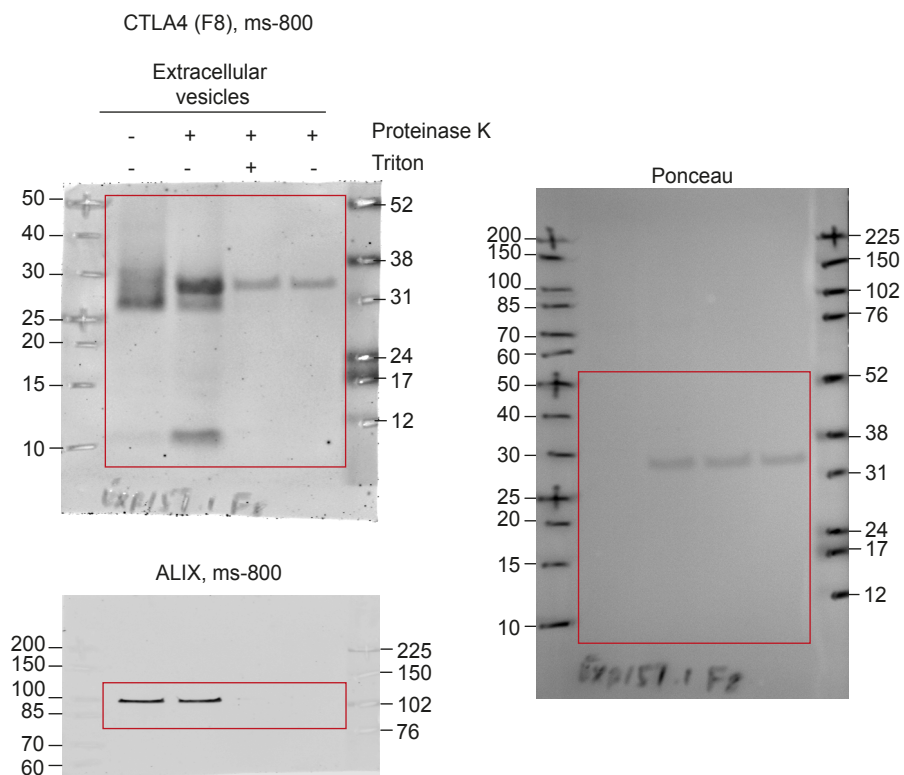

Supplement: SourceData F2 — is the source file for Fig. 2. [file JCB_202312141_SourceDataF2.pdf]

Fig3A

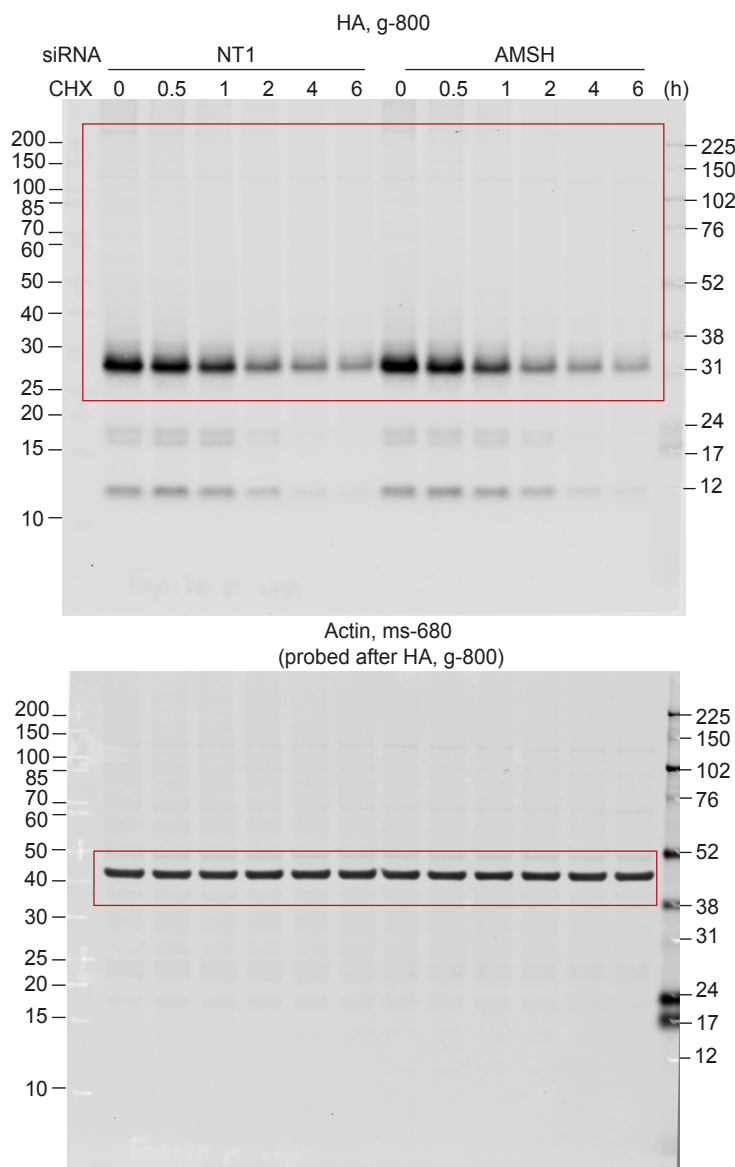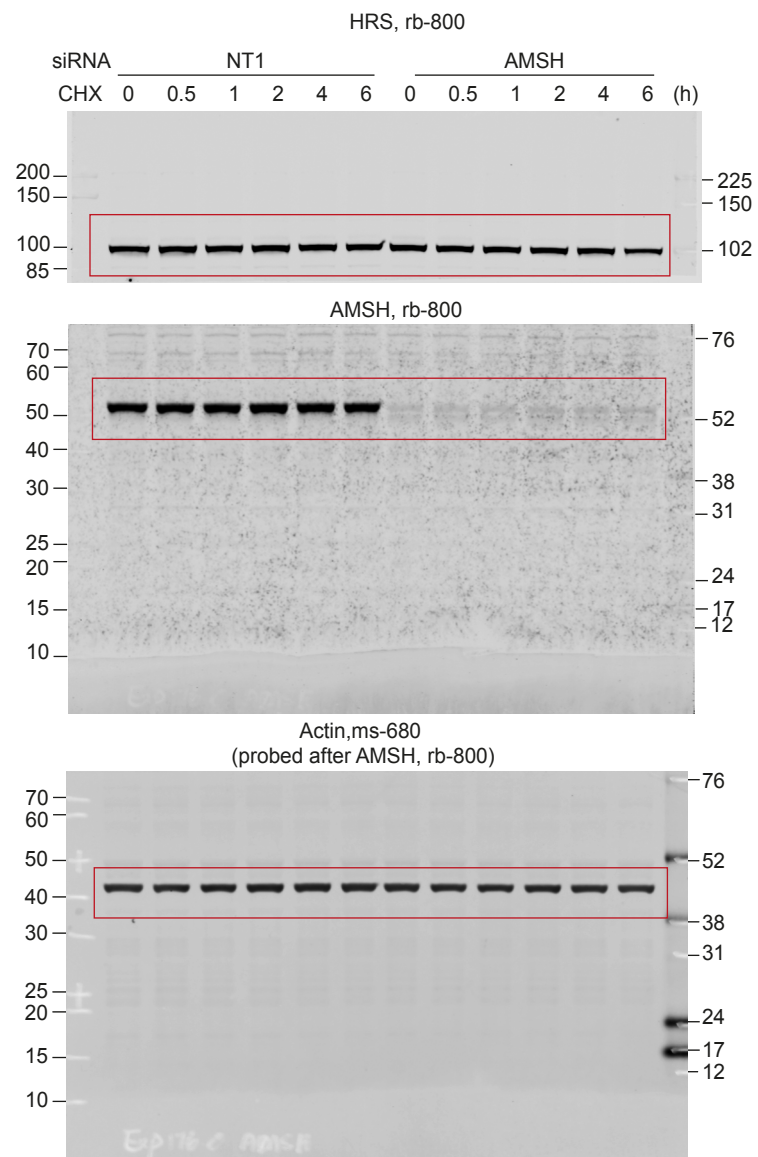

Fig3B

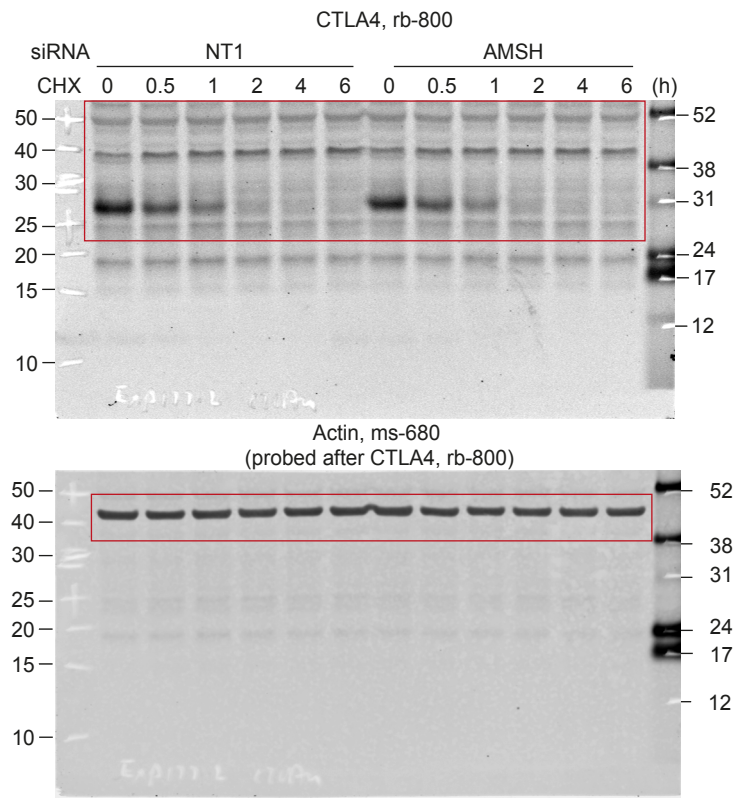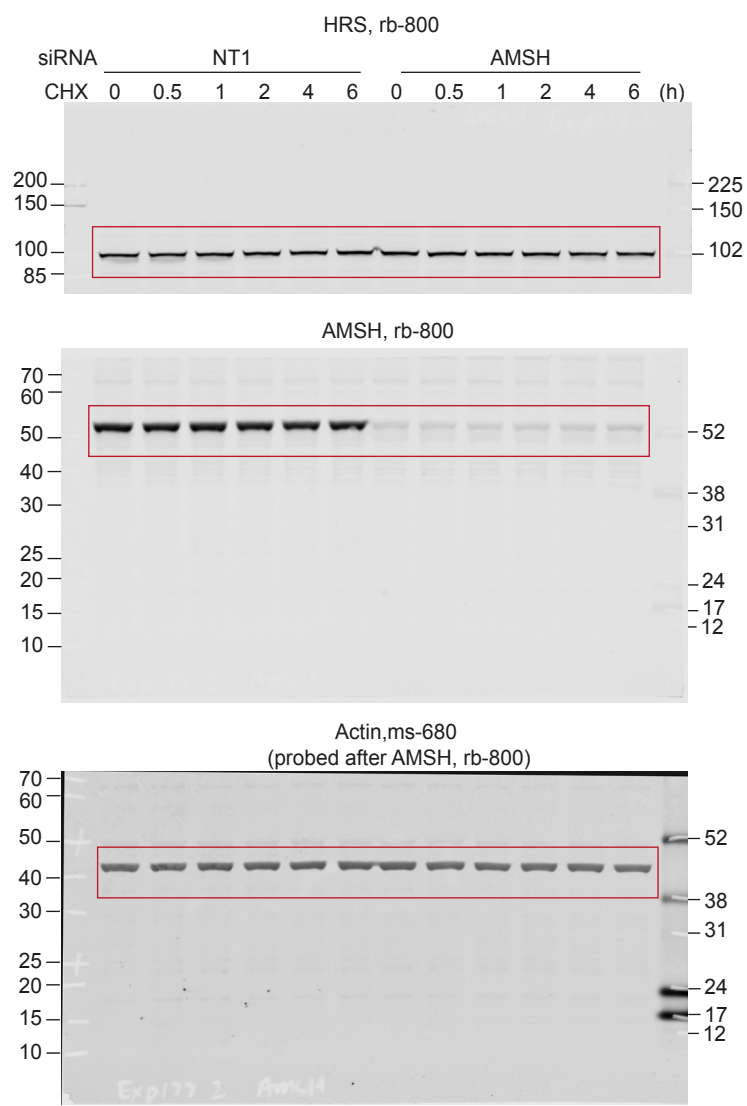

Fig3C

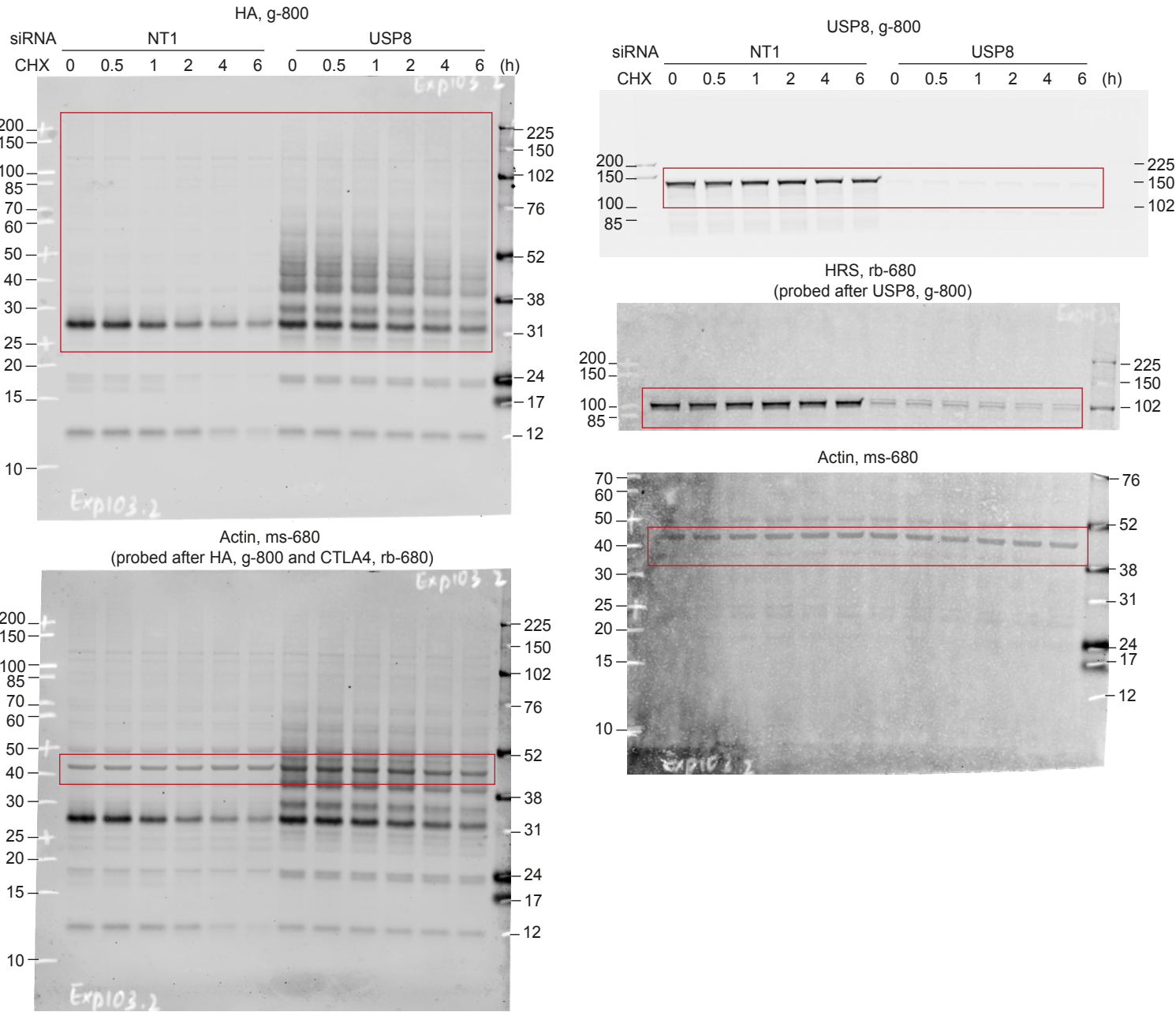

Fig3D

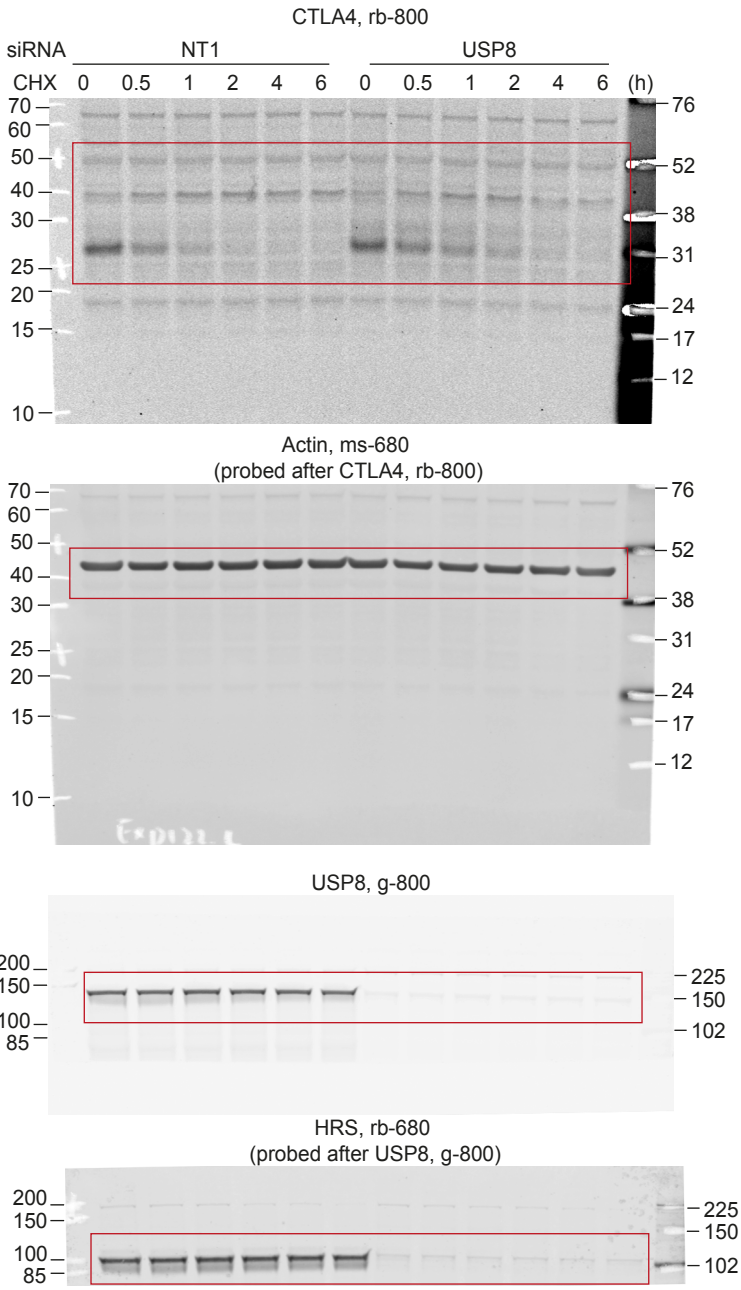

Fig3E

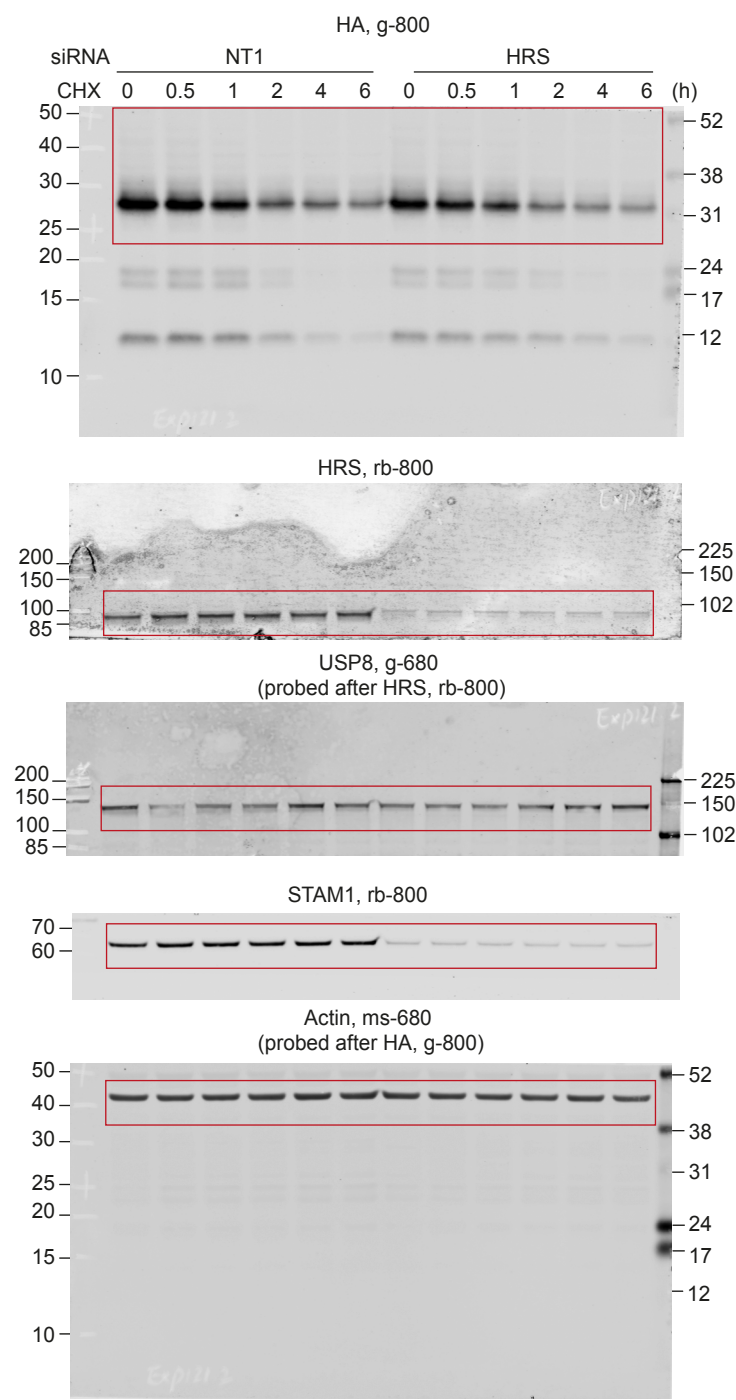

Fig3F

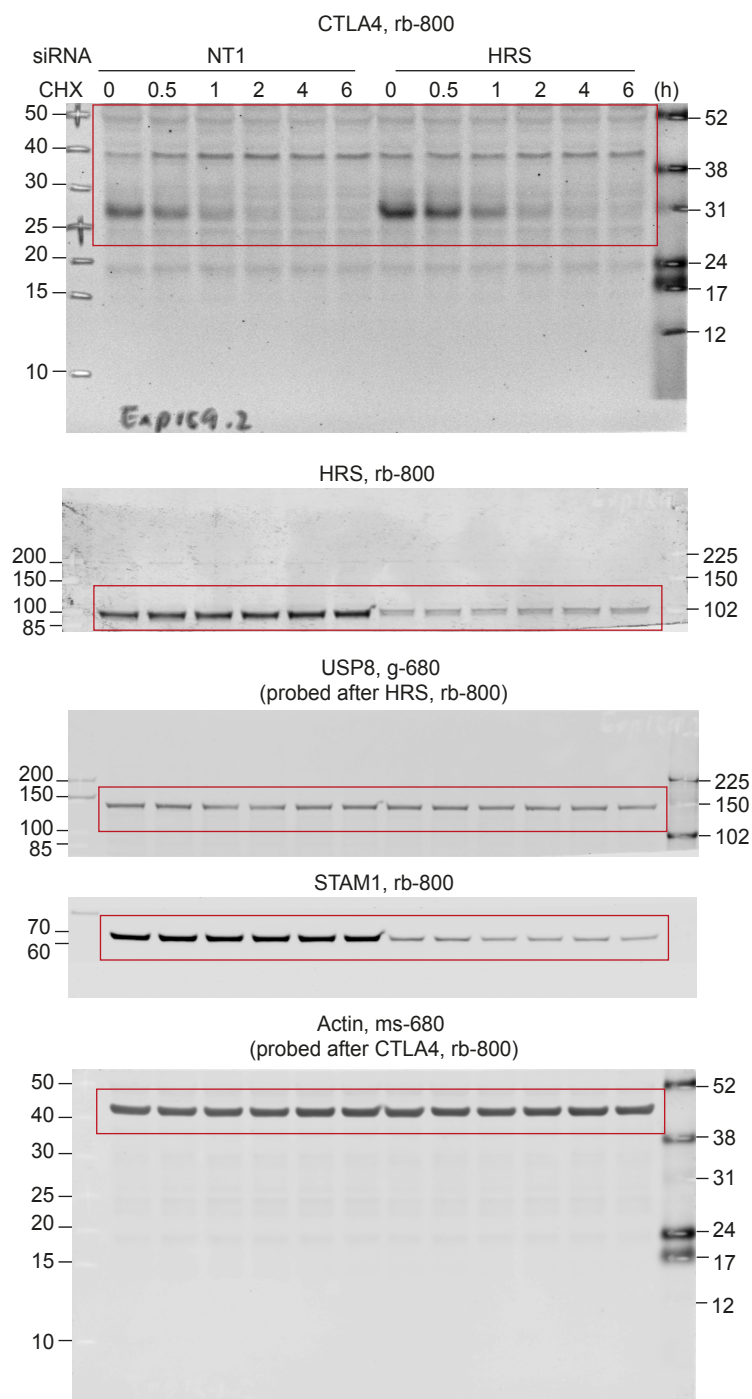

Supplement: SourceData F3 — is the source file for Fig. 3. [file JCB_202312141_SourceDataF3.pdf]

Fig4A: HeLa S3 FlpIn CTLA4-HA

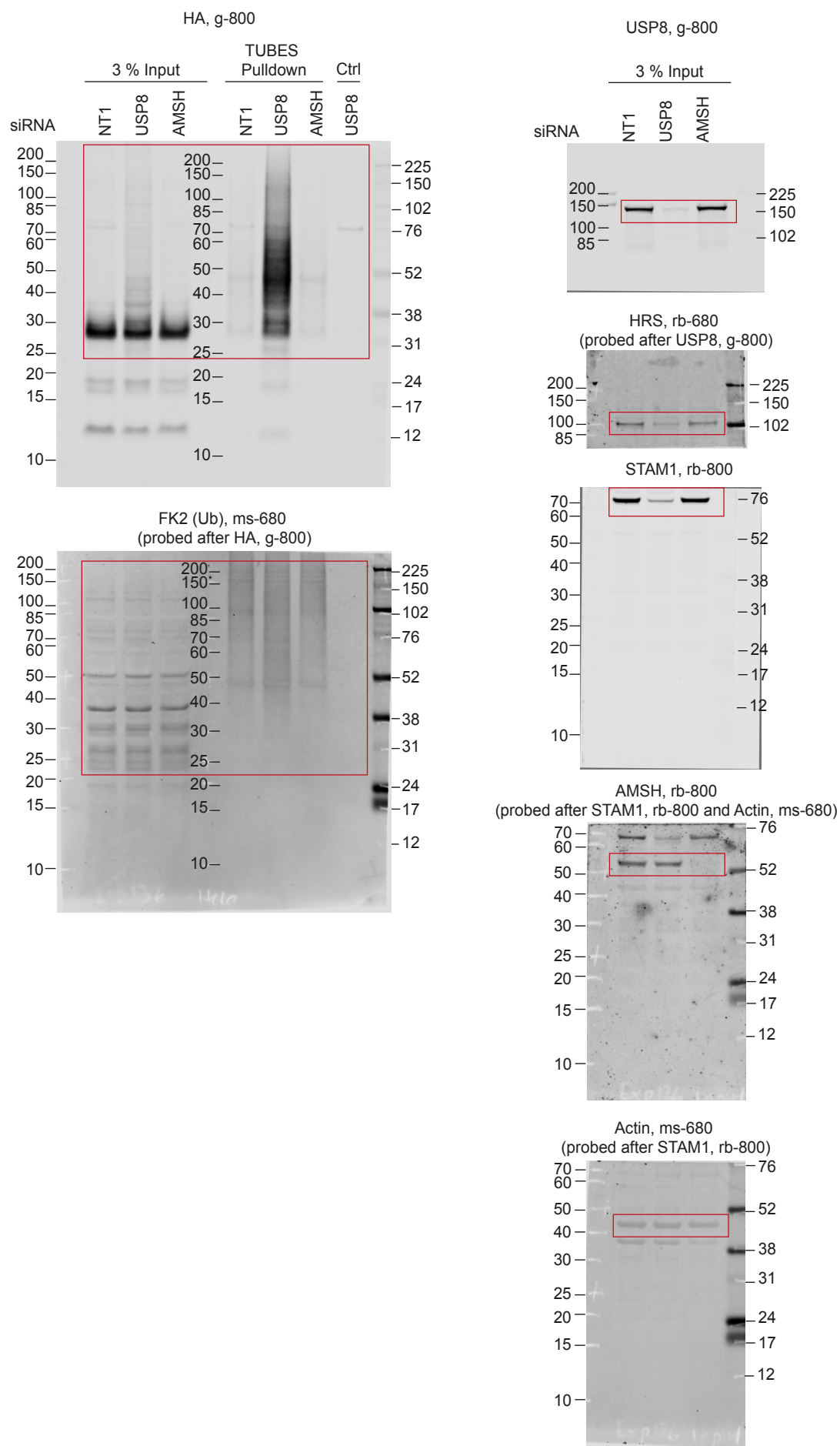

Fig4A: A2058

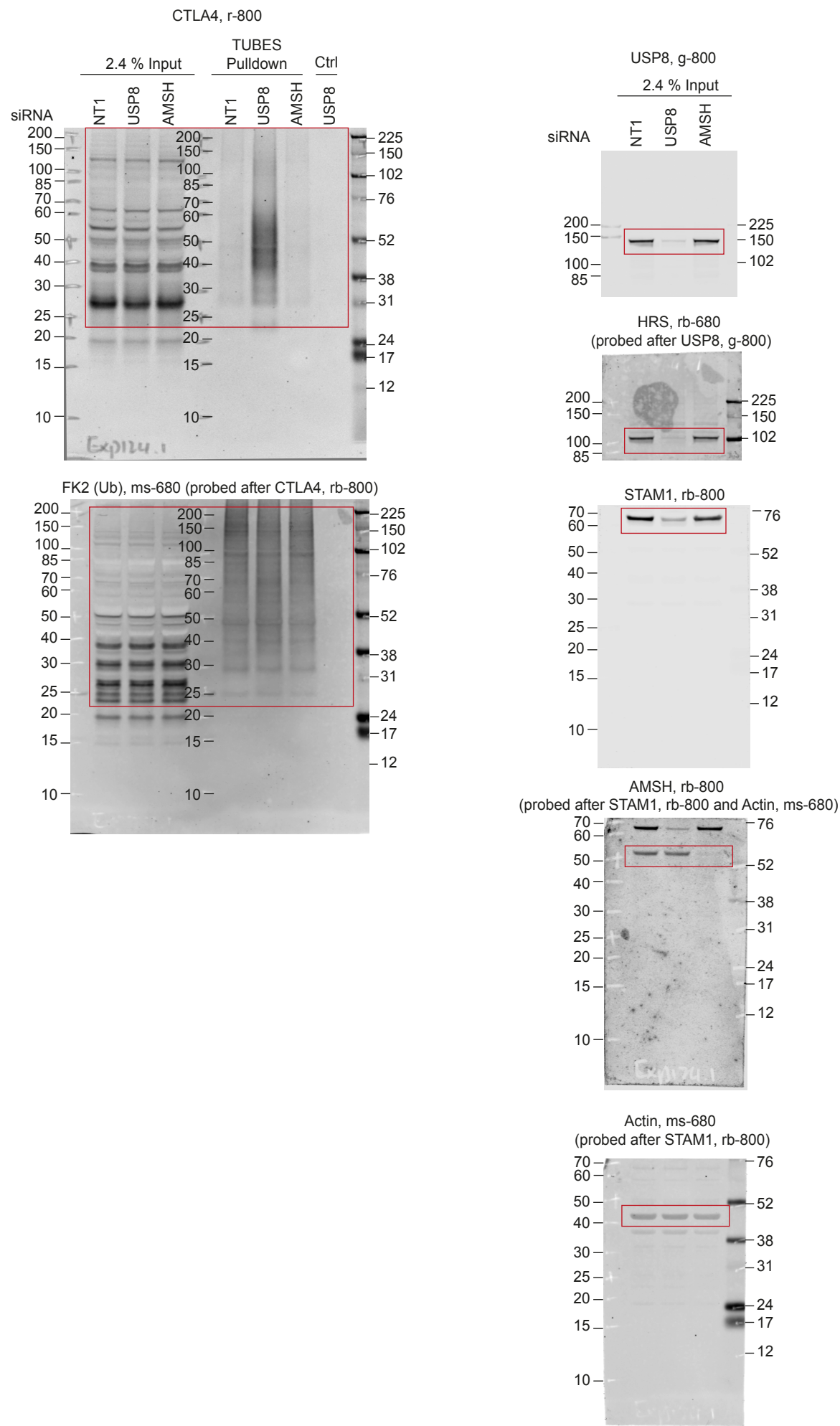

Fig4C

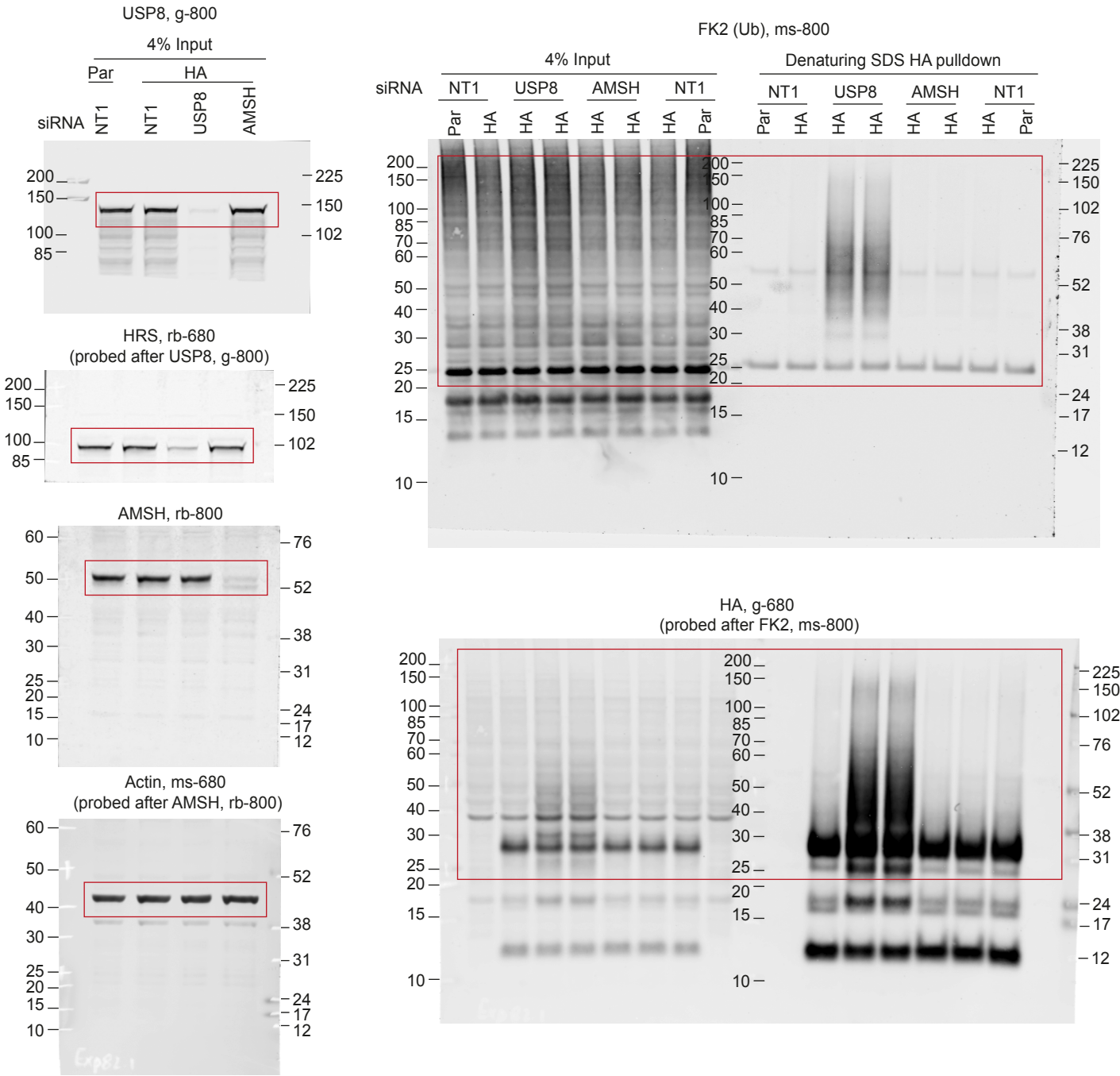

Fig4E

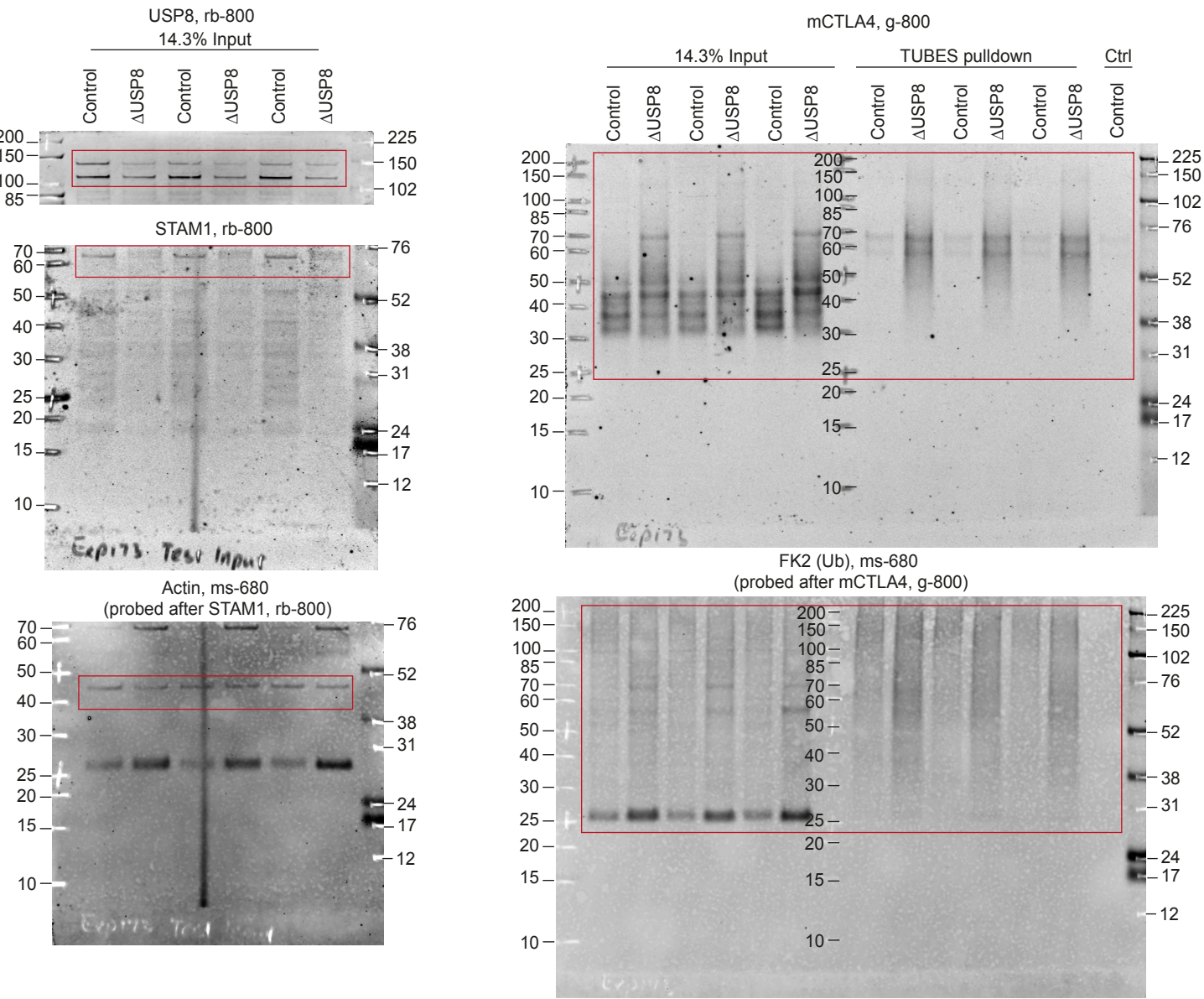

Supplement: SourceData F4 — is the source file for Fig. 4. [file JCB_202312141_SourceDataF4.pdf]

Fig5A

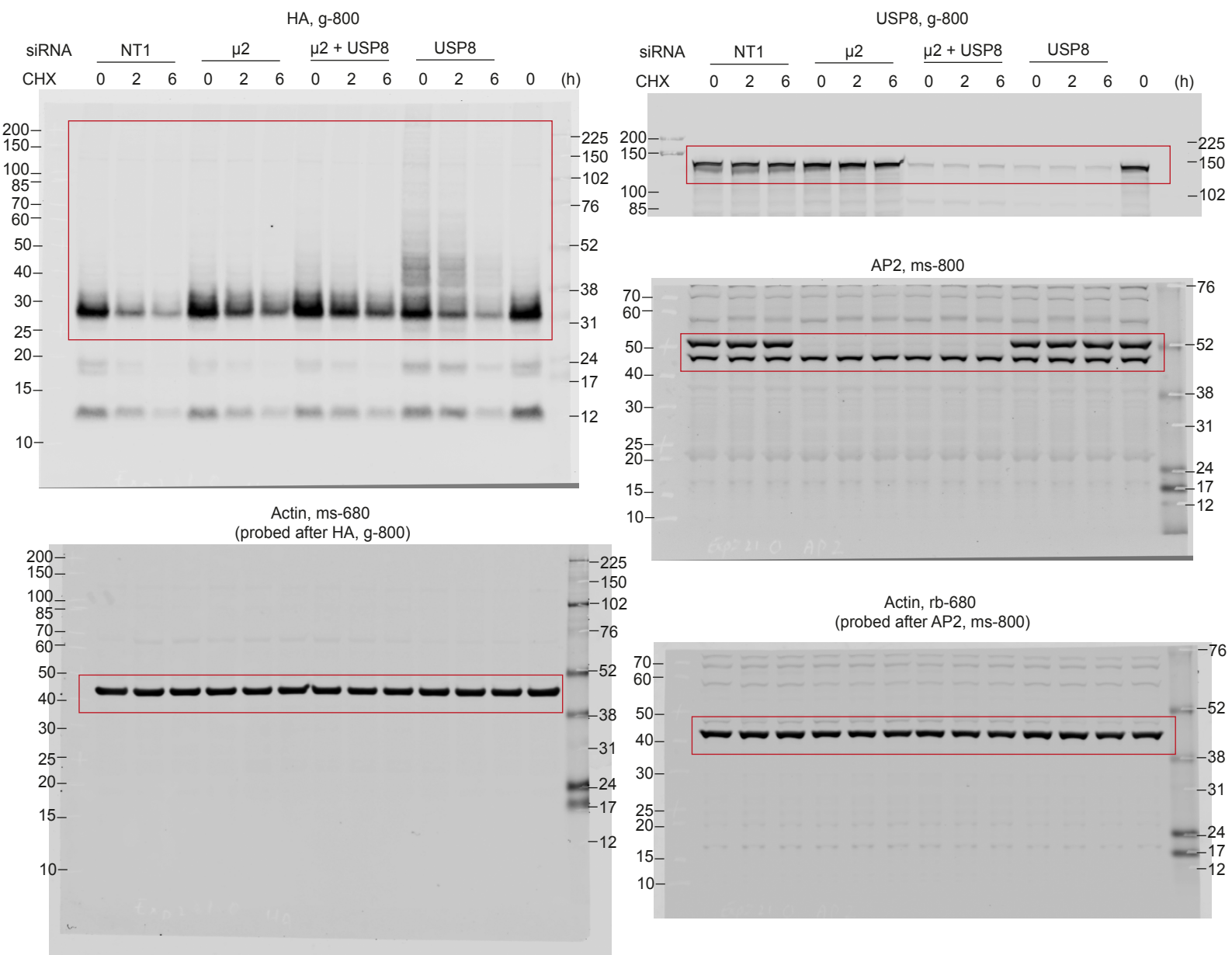

Fig5D

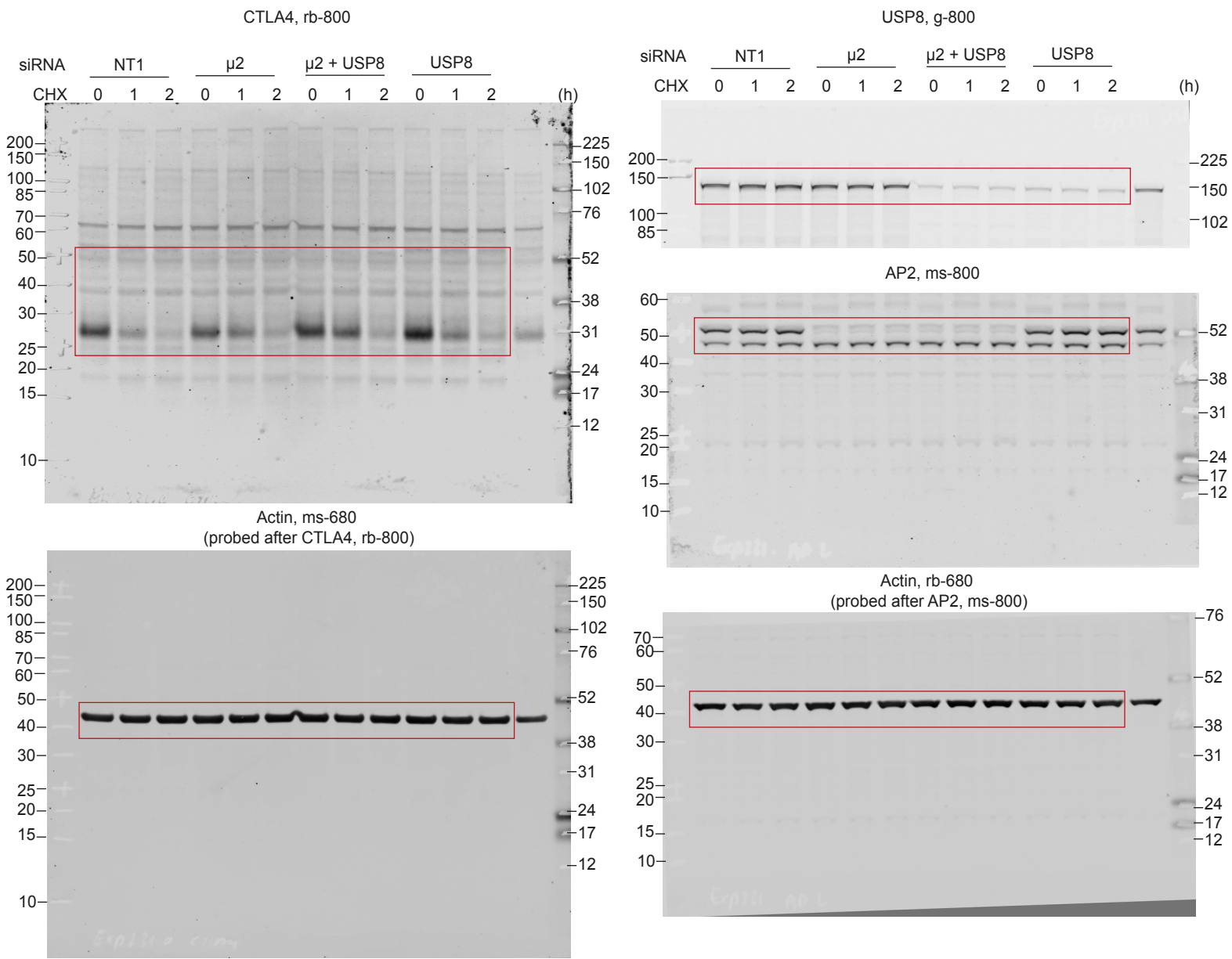

Fig5G

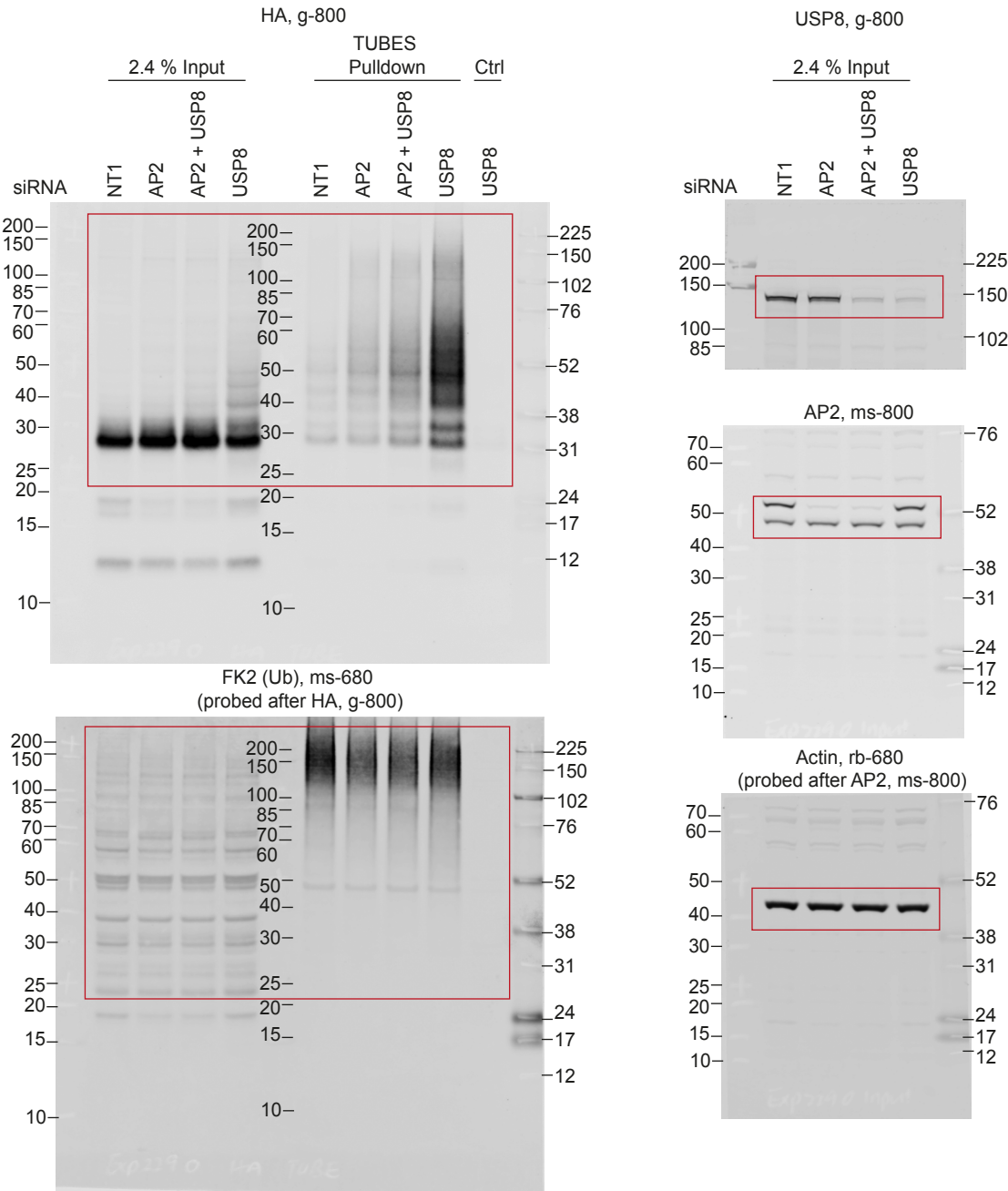

Fig5H

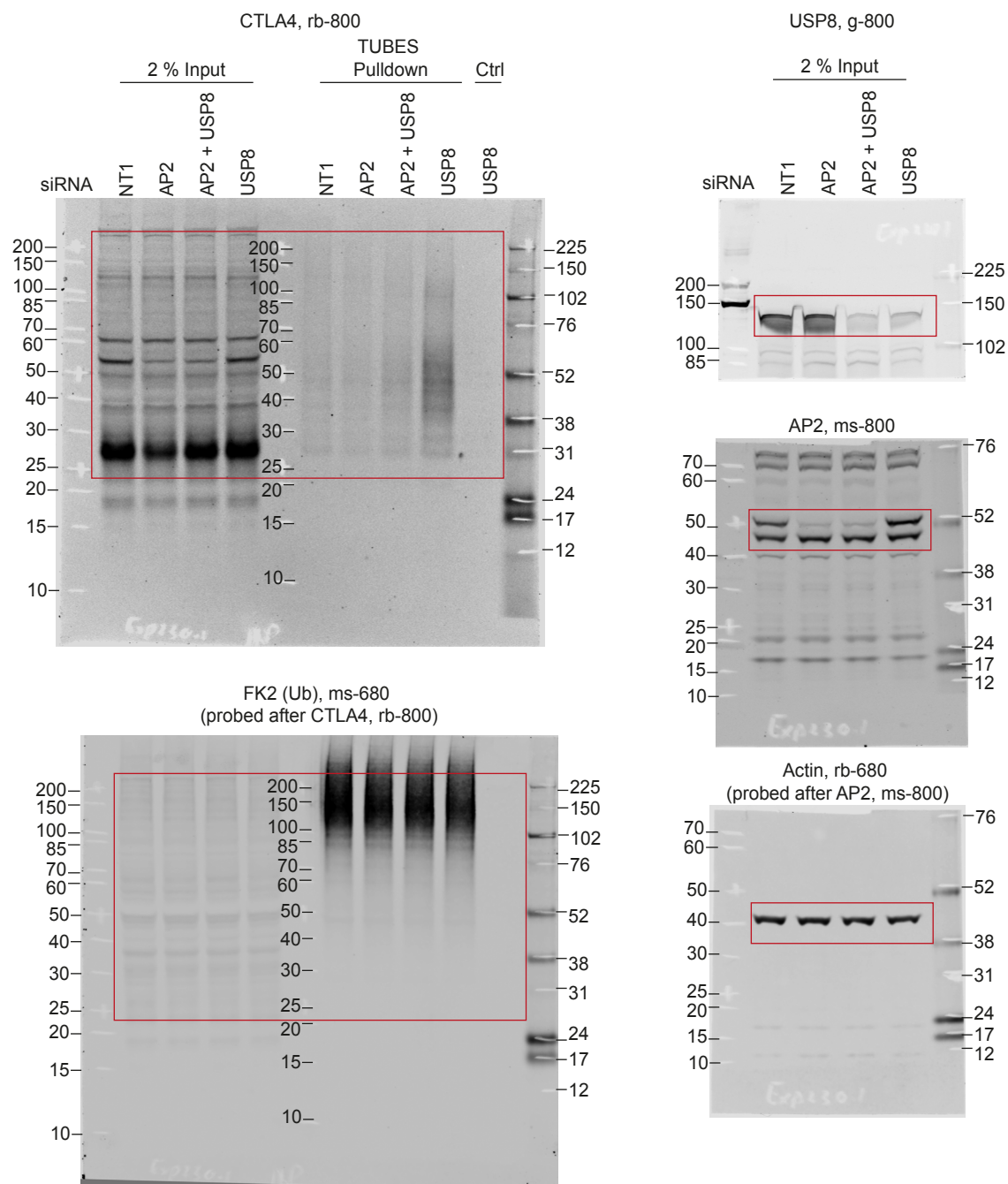

Supplement: SourceData F5 — is the source file for Fig. 5. [file JCB_202312141_SourceDataF5.pdf]

Fig7A

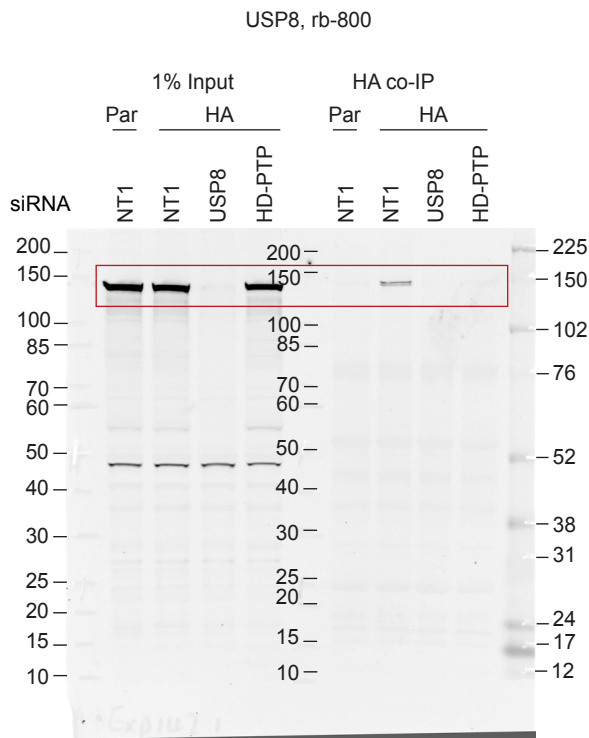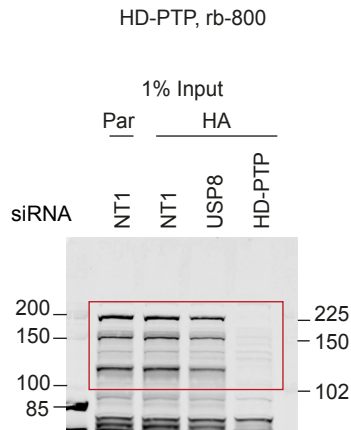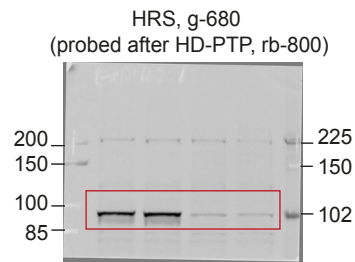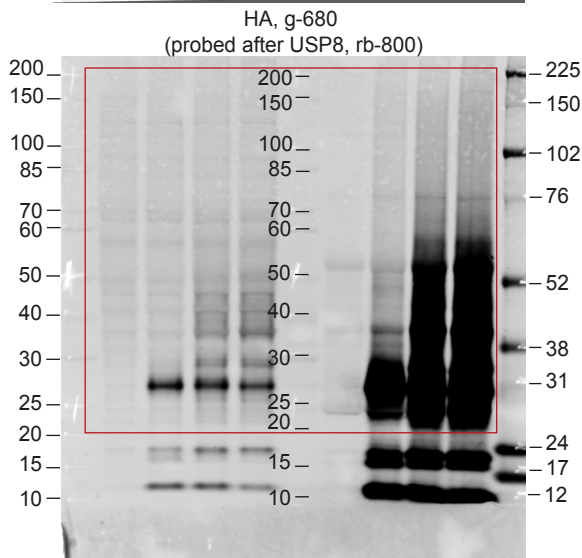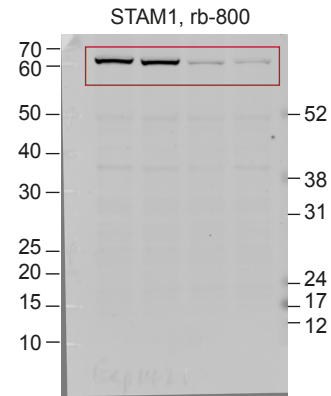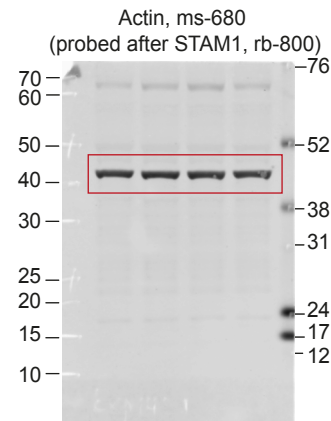

Fig7B

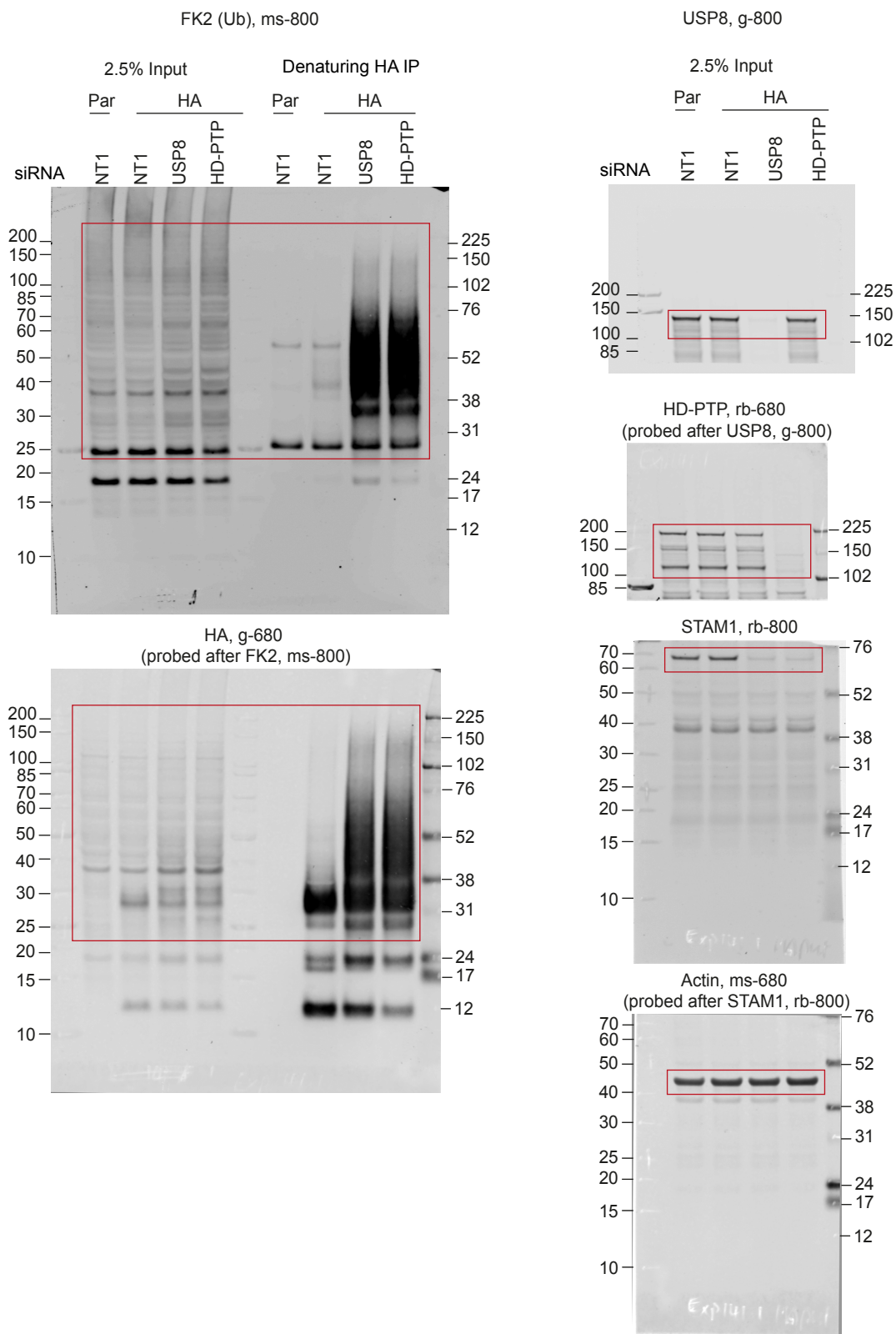

Fig7D: HeLa S3 Flp-In CTLA4-HA

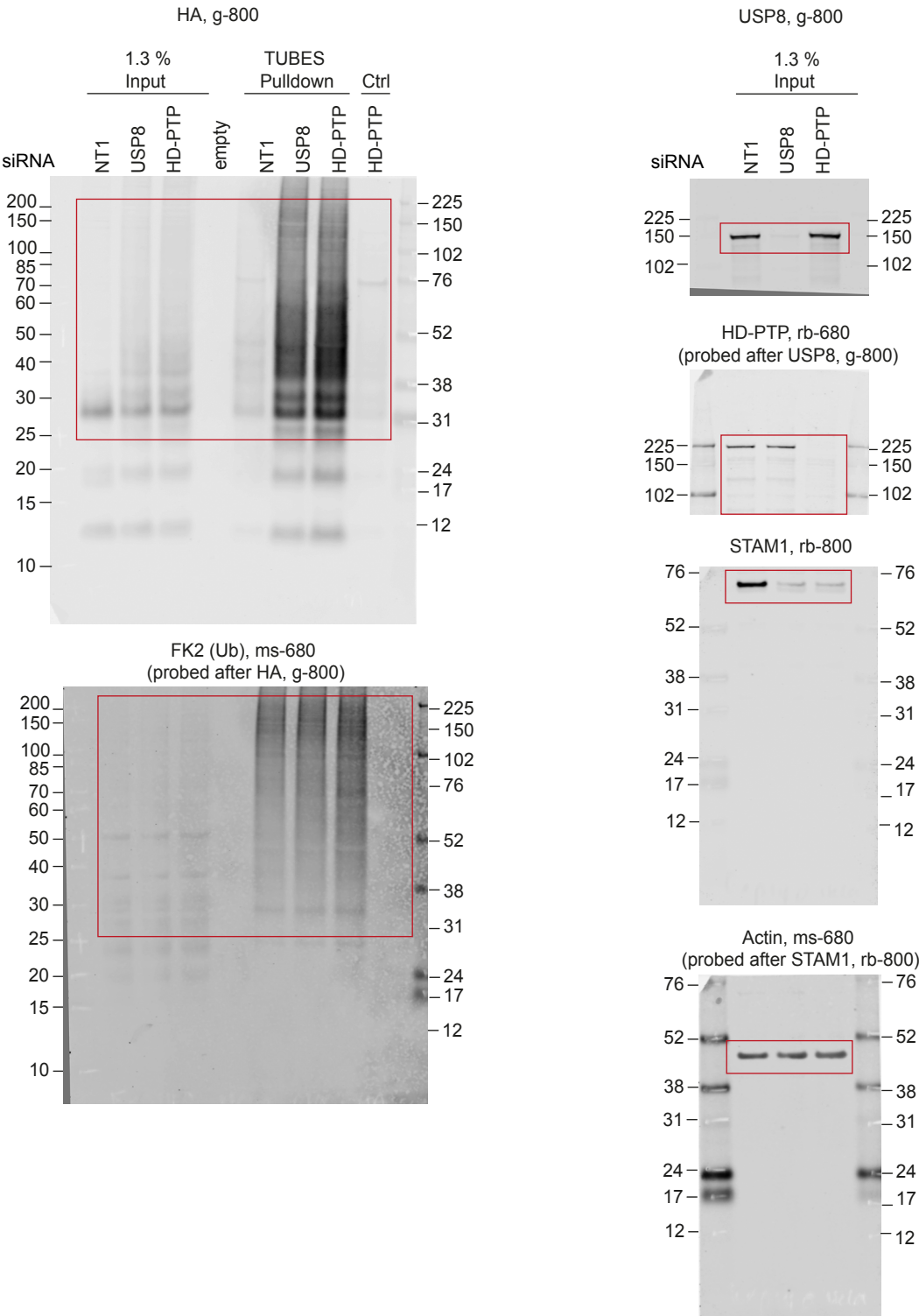

Fig7D: A2058

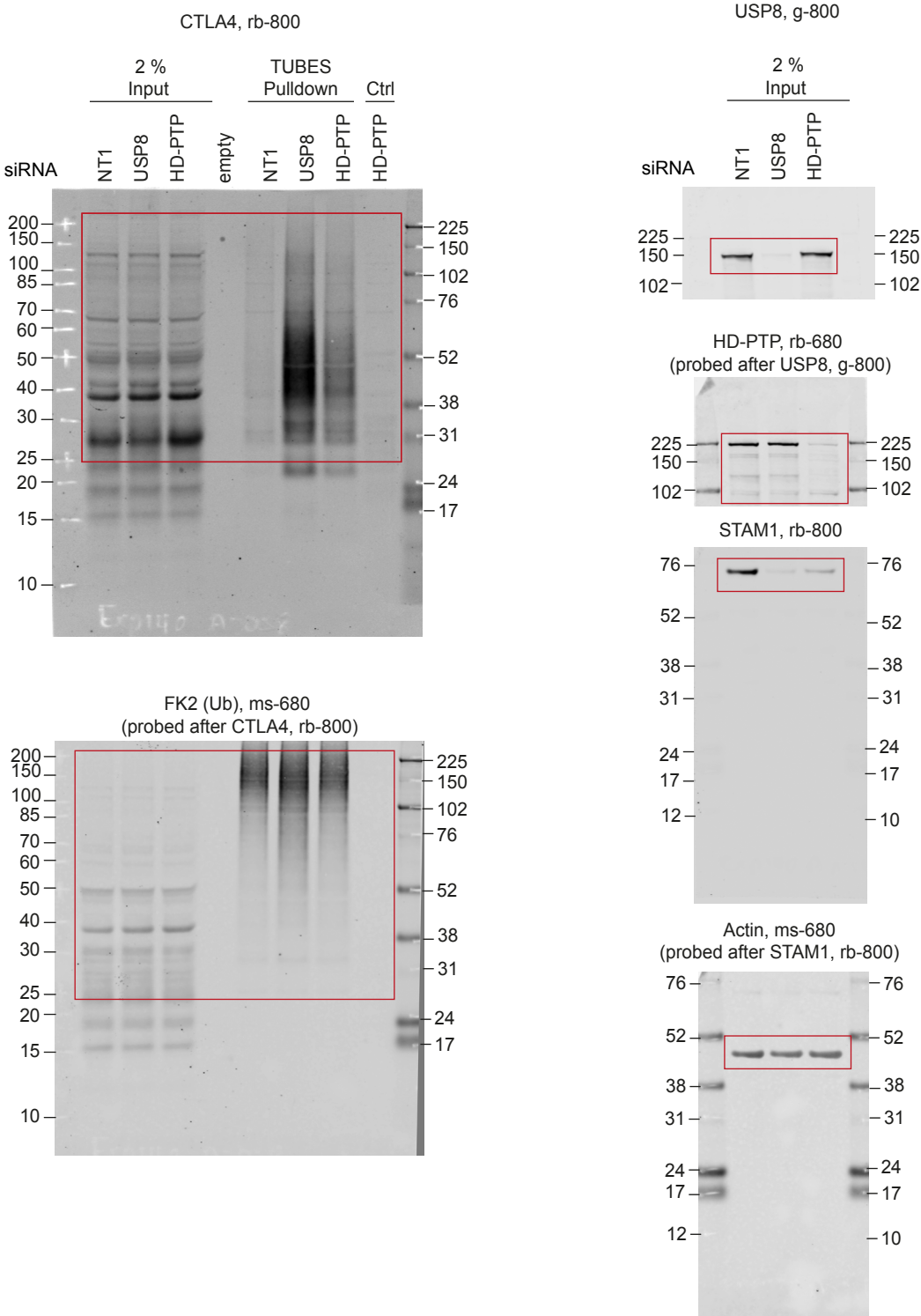

Fig7F: HeLa S3 Flp-In CTLA4-HA

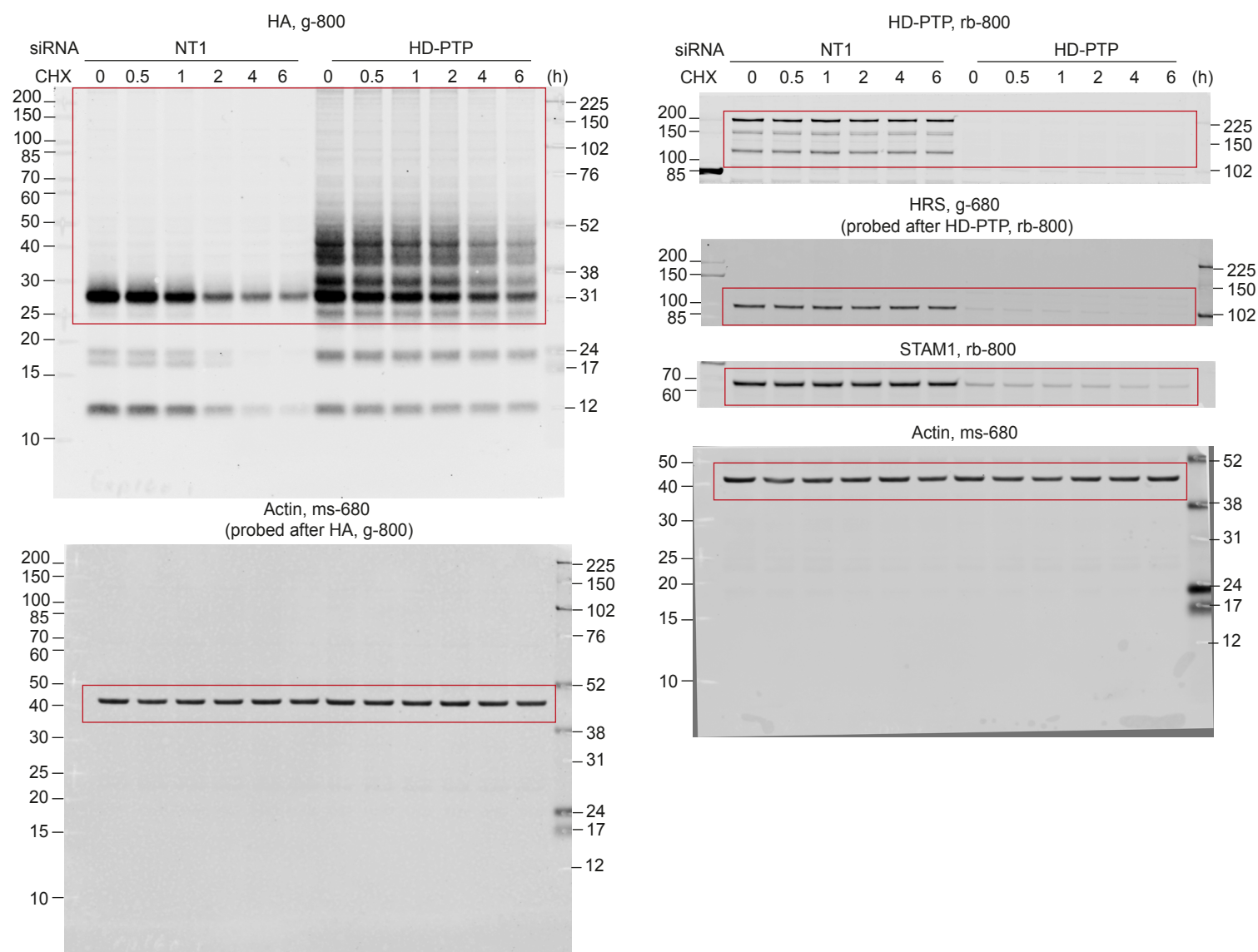

Fig7F: A2058

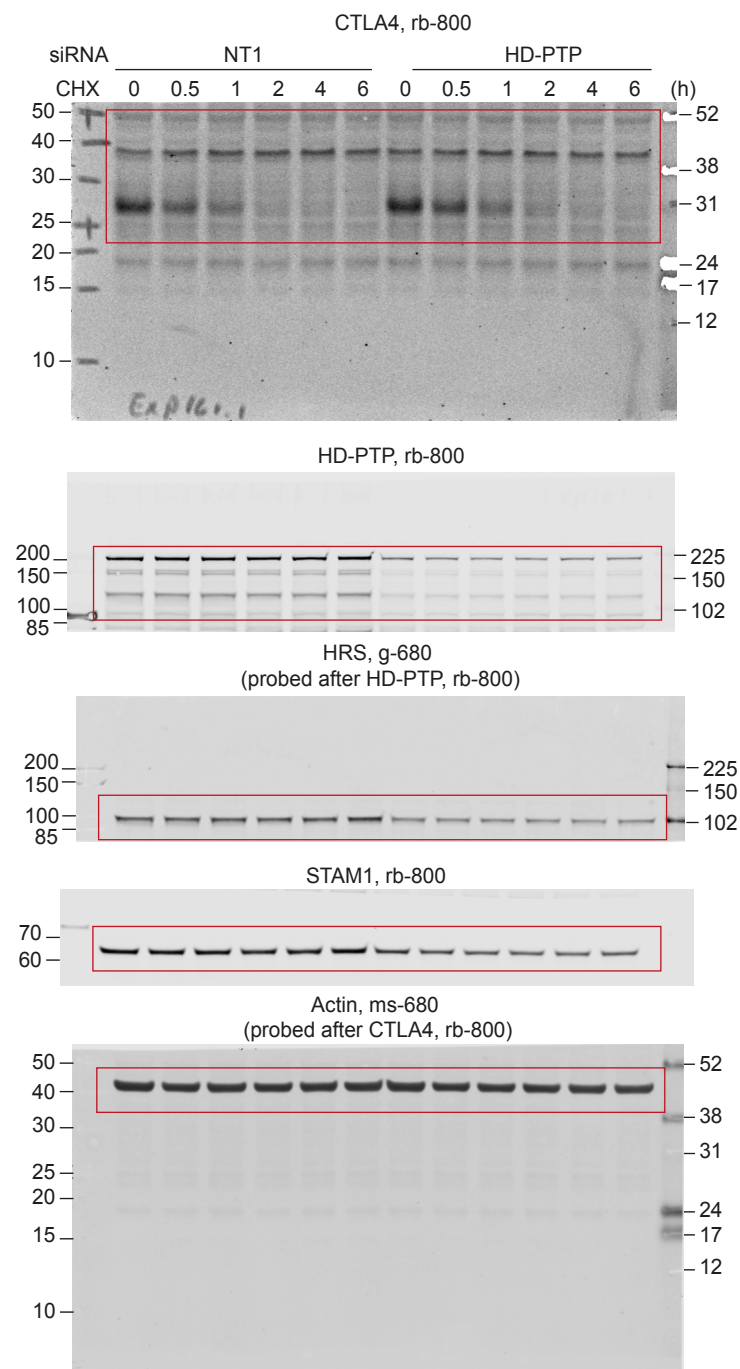

Supplement: SourceData F7 — is the source file for Fig. 7. [file JCB_202312141_SourceDataF7.pdf]

Fig8E (continue to the next page)

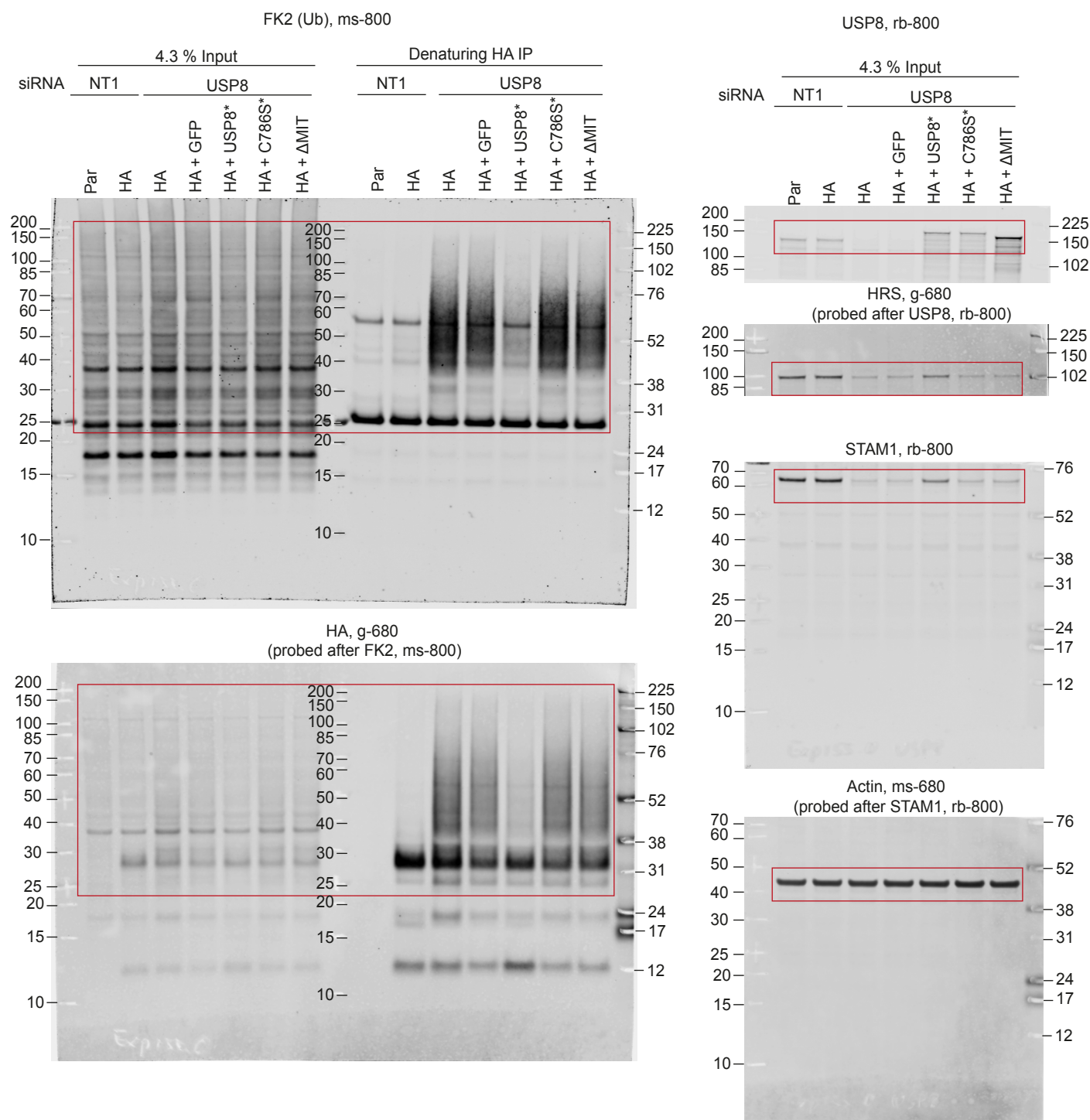

Fig8E (continued)

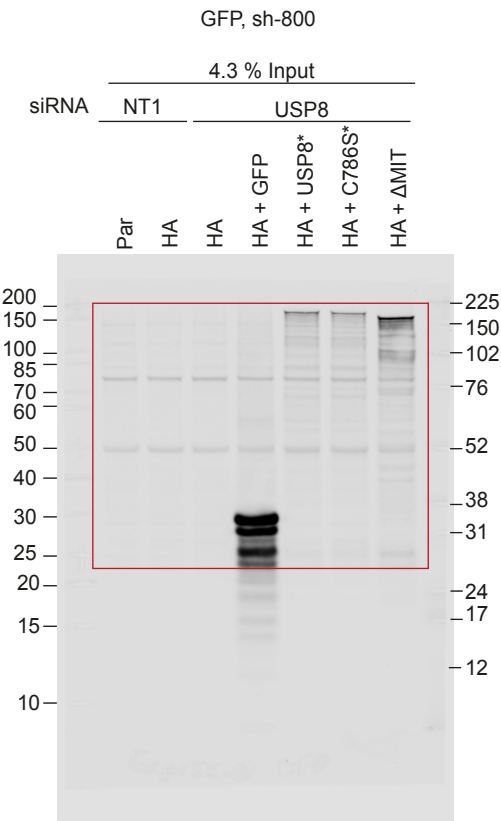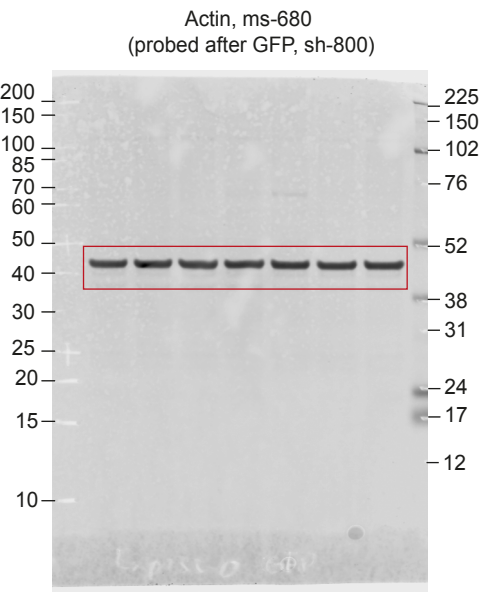

Supplement: SourceData F8 — is the source file for Fig. 8. [file JCB_202312141_SourceDataF8.pdf]

Fig9B

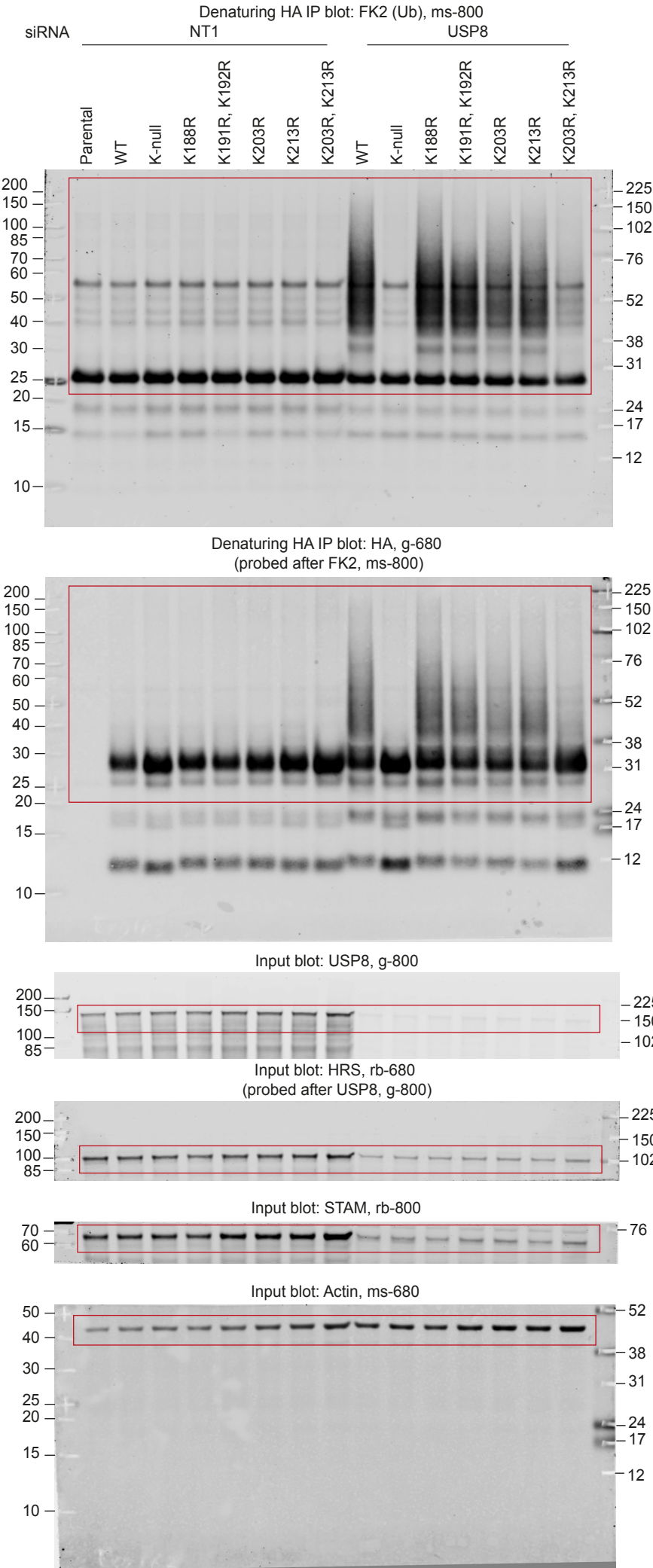

Fig9B (continued)

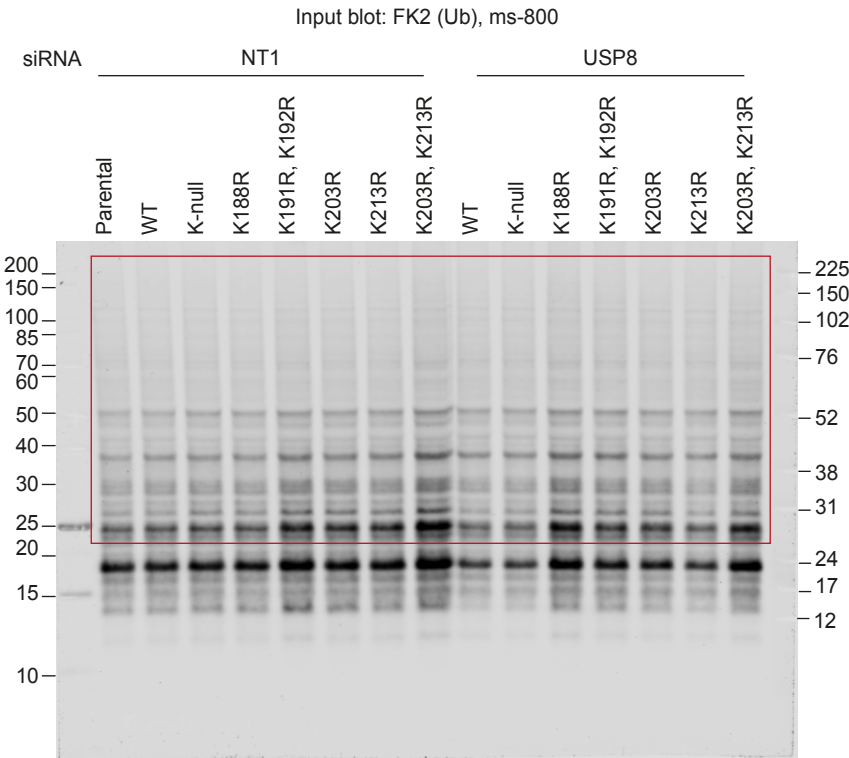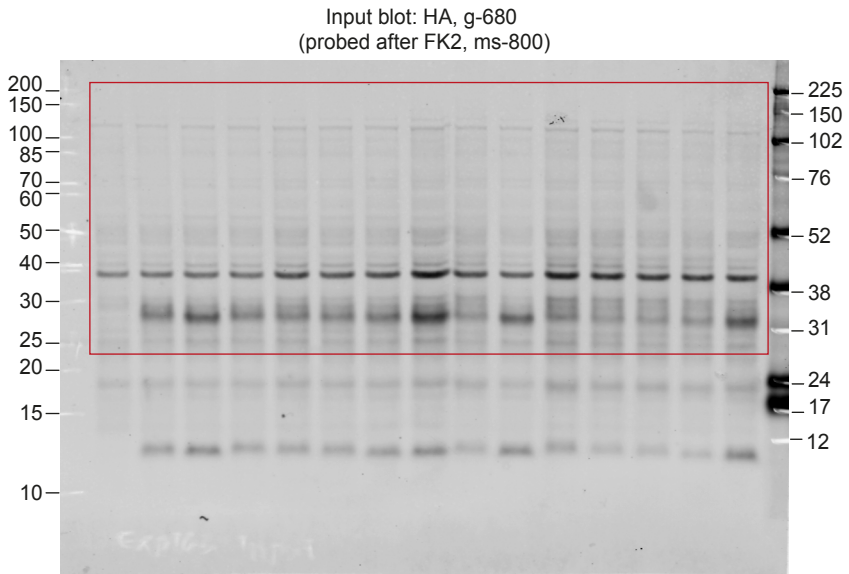

Fig9D

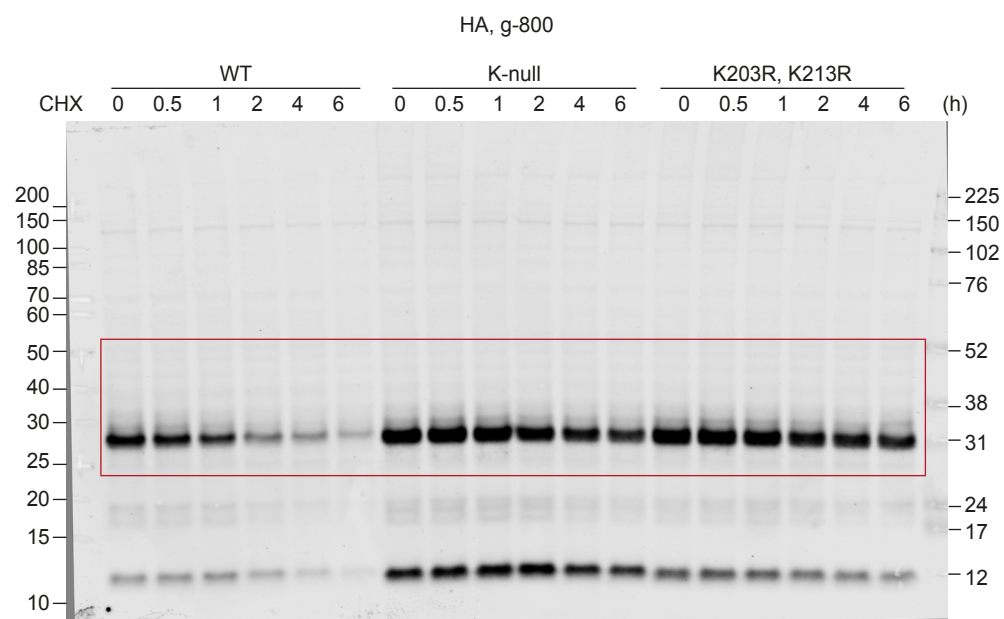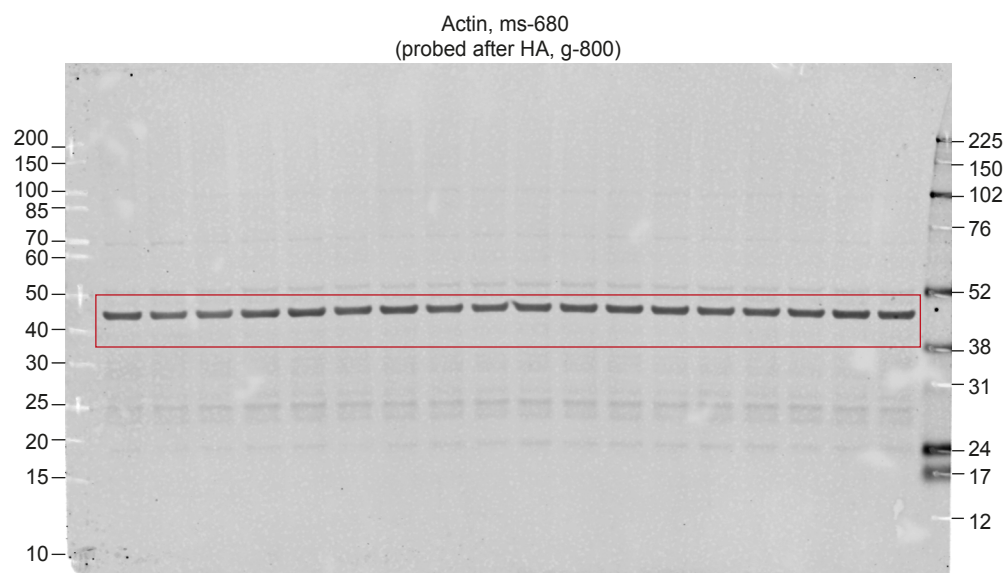

Fig9D (continued)

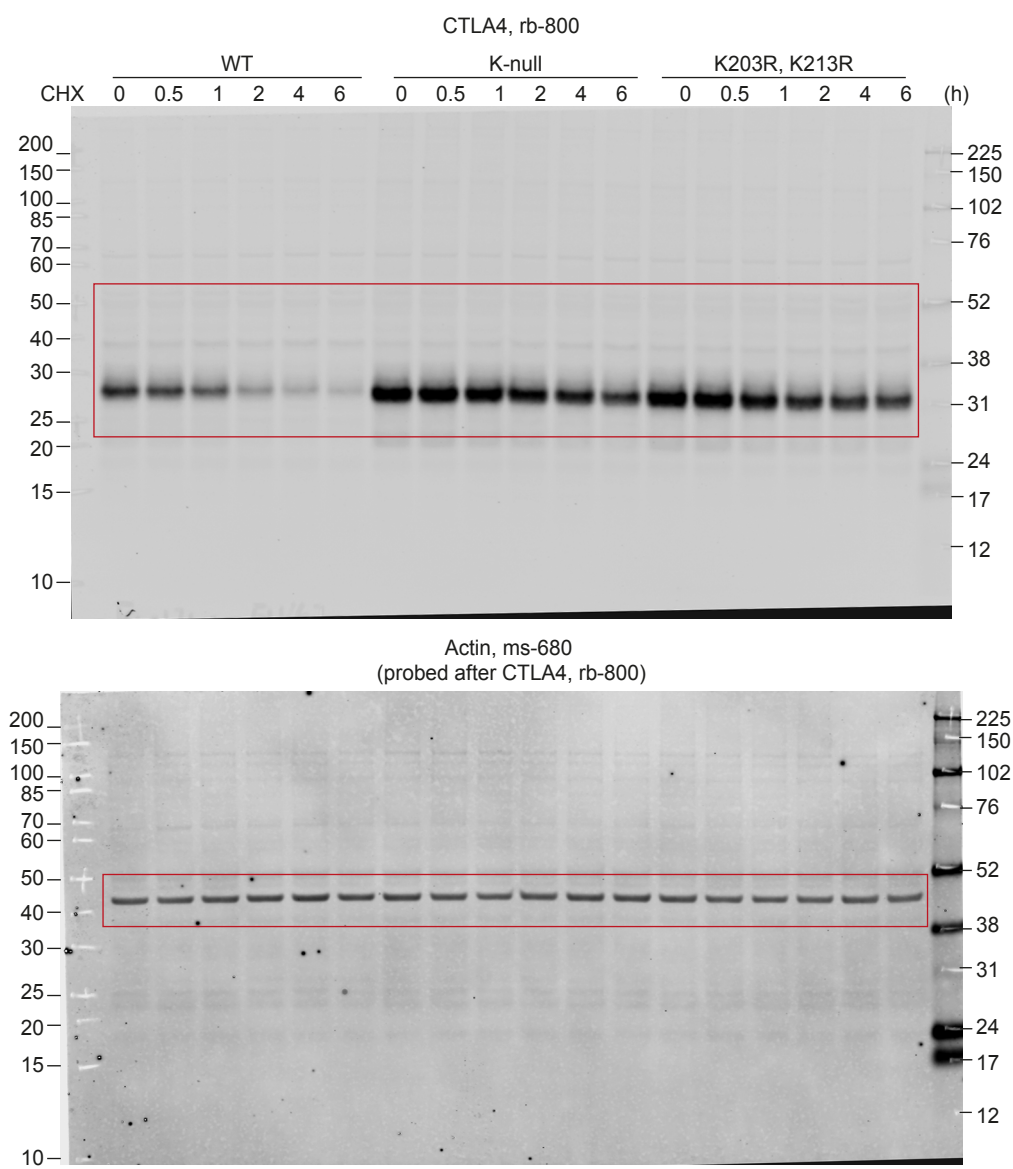

Supplement: SourceData F9 — is the source file for Fig. 9. [file JCB_202312141_SourceDataF9.pdf]

Fig10A

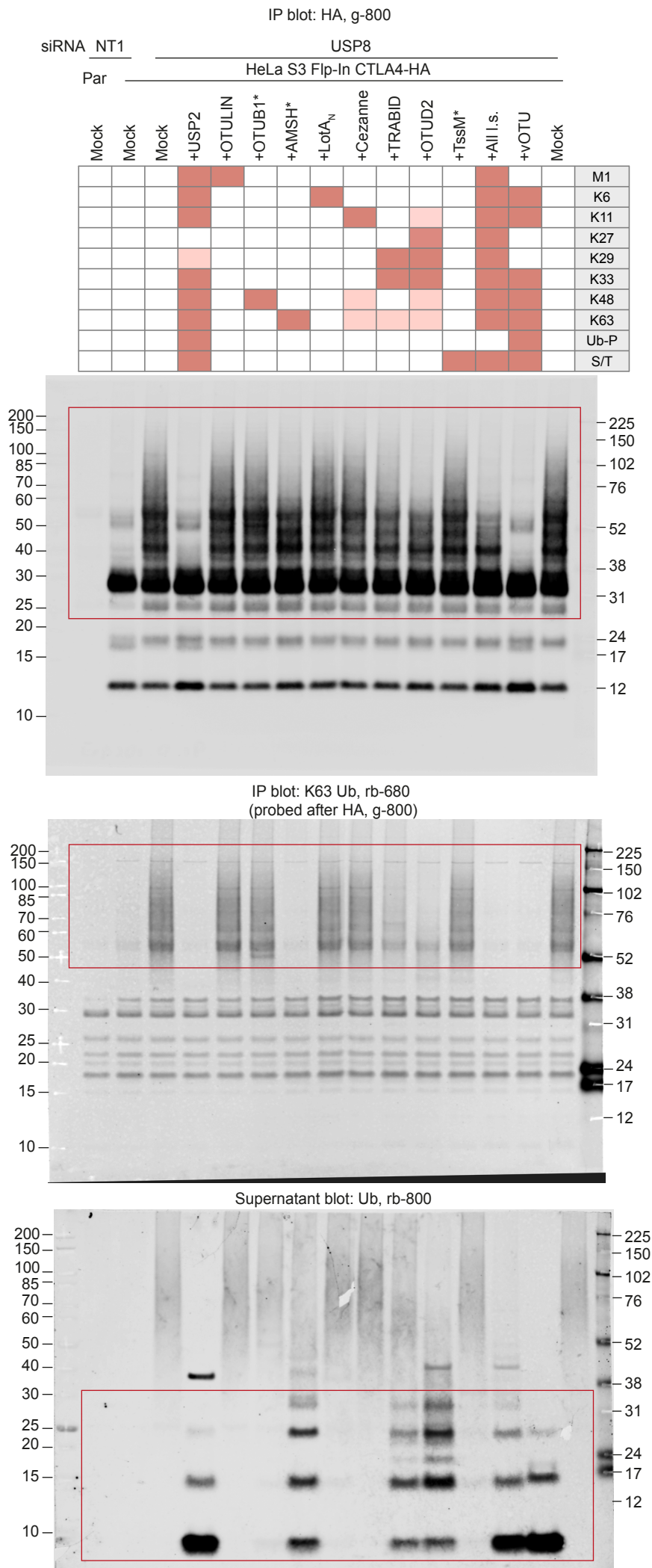

Fig10C

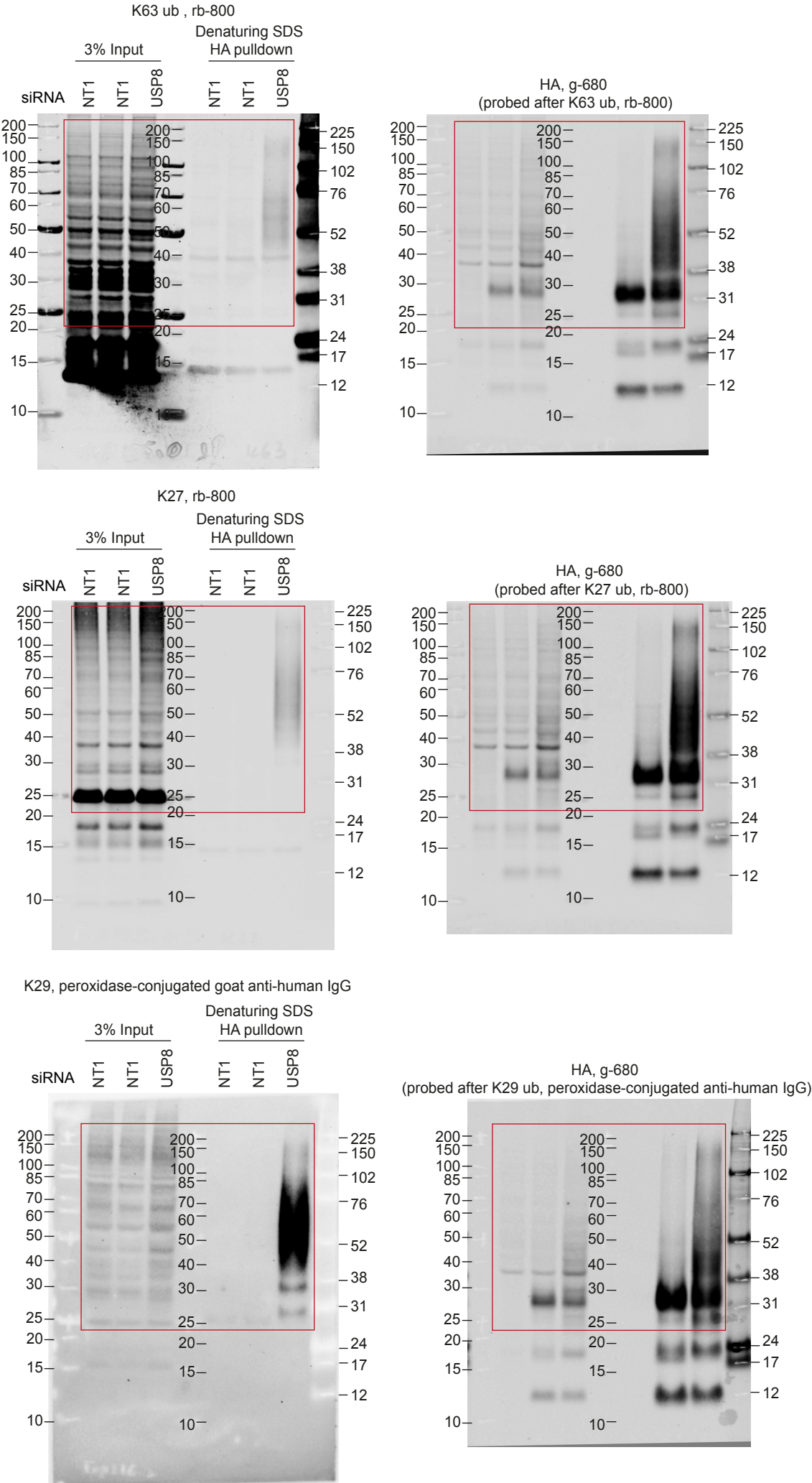

Supplement: SourceData F10 — is the source file for Fig. 10. [file JCB_202312141_SourceDataF10.pdf]

Supplementary Figure 1B

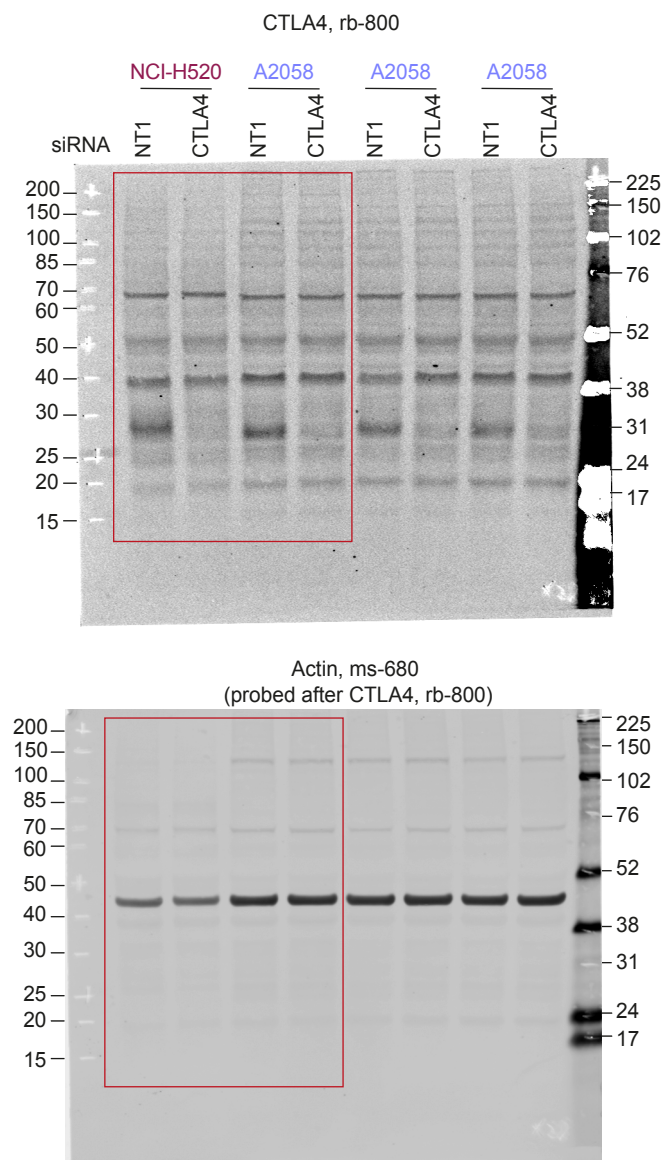

Supplementary Figure 1C

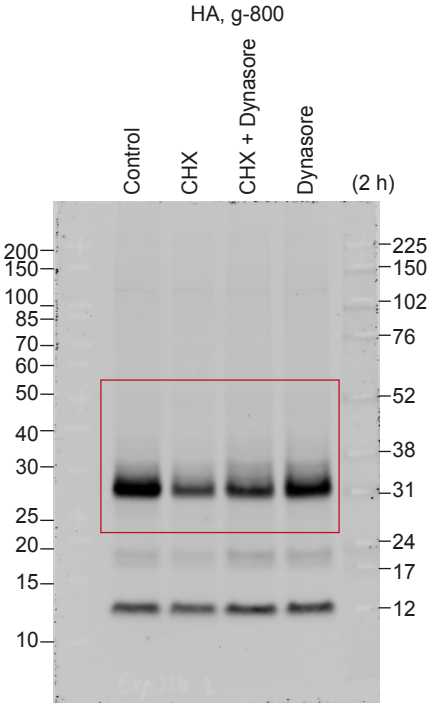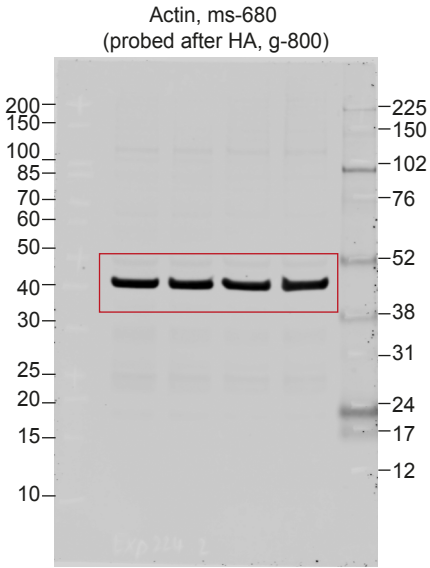

Supplement: SourceData FS1 — is the source file for Fig. S1. [file JCB_202312141_SourceDataFS1.pdf]

Supplementary S3B: Lysates

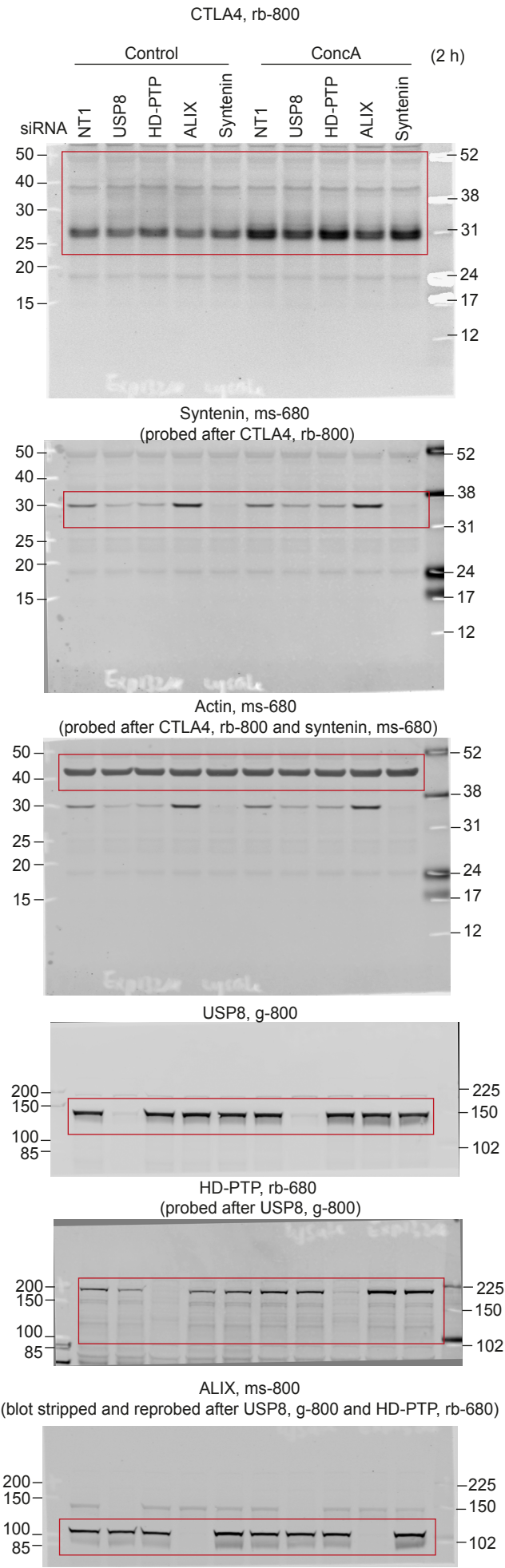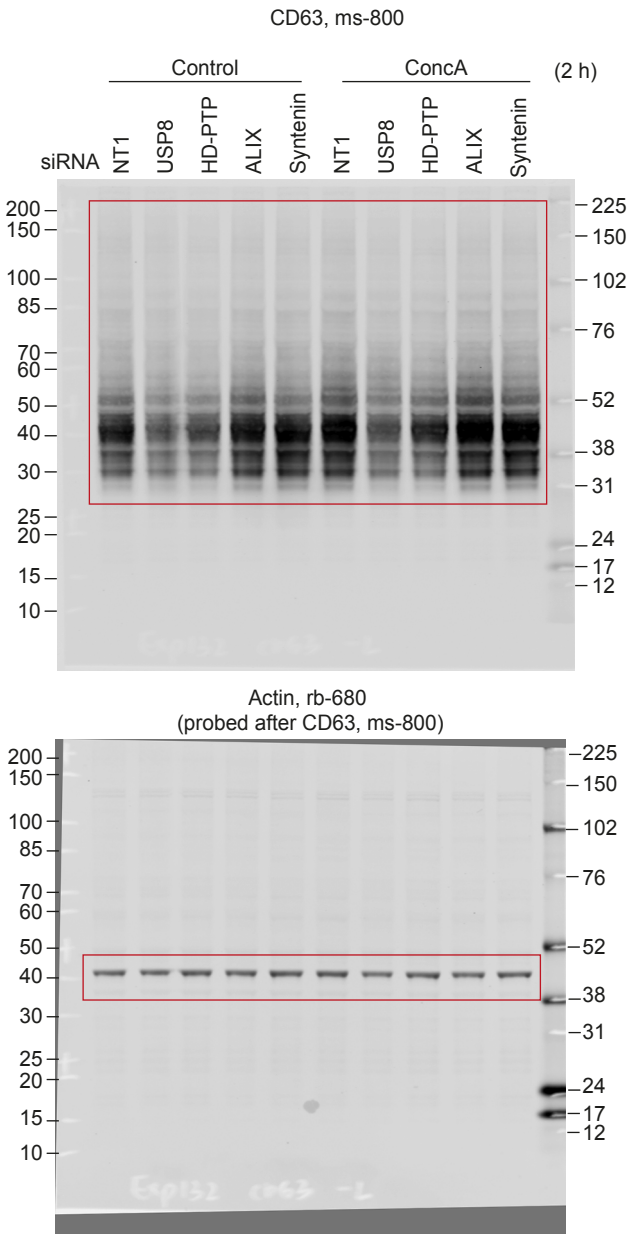

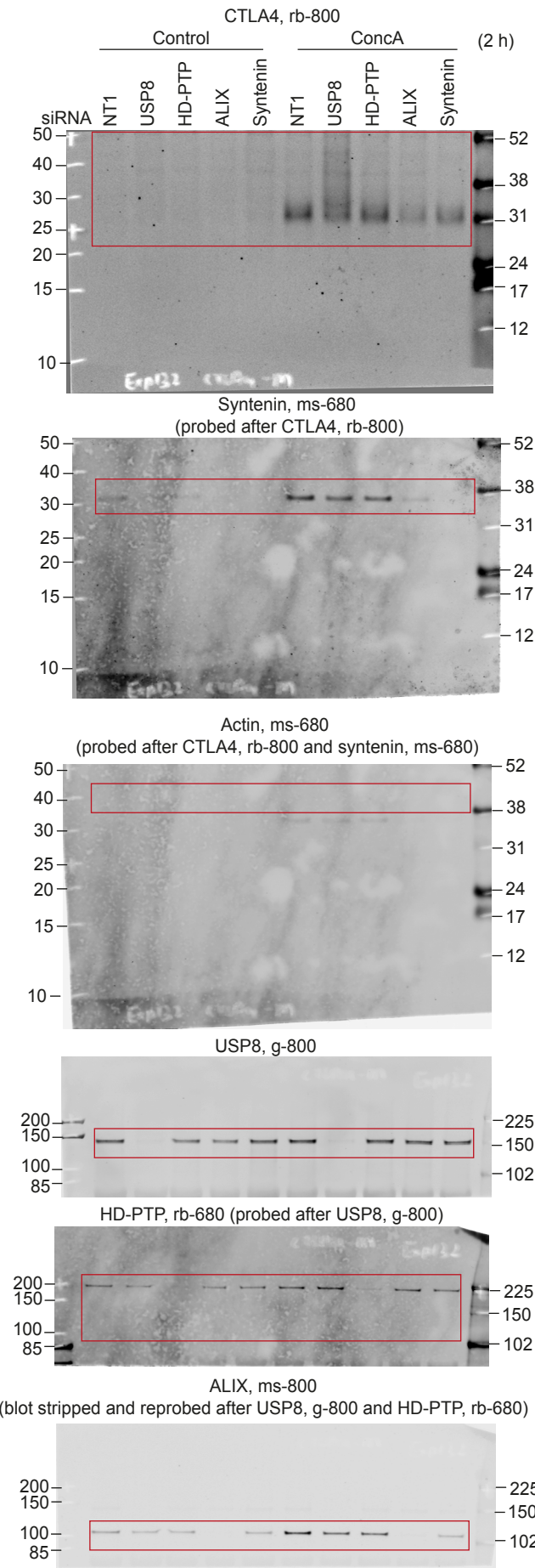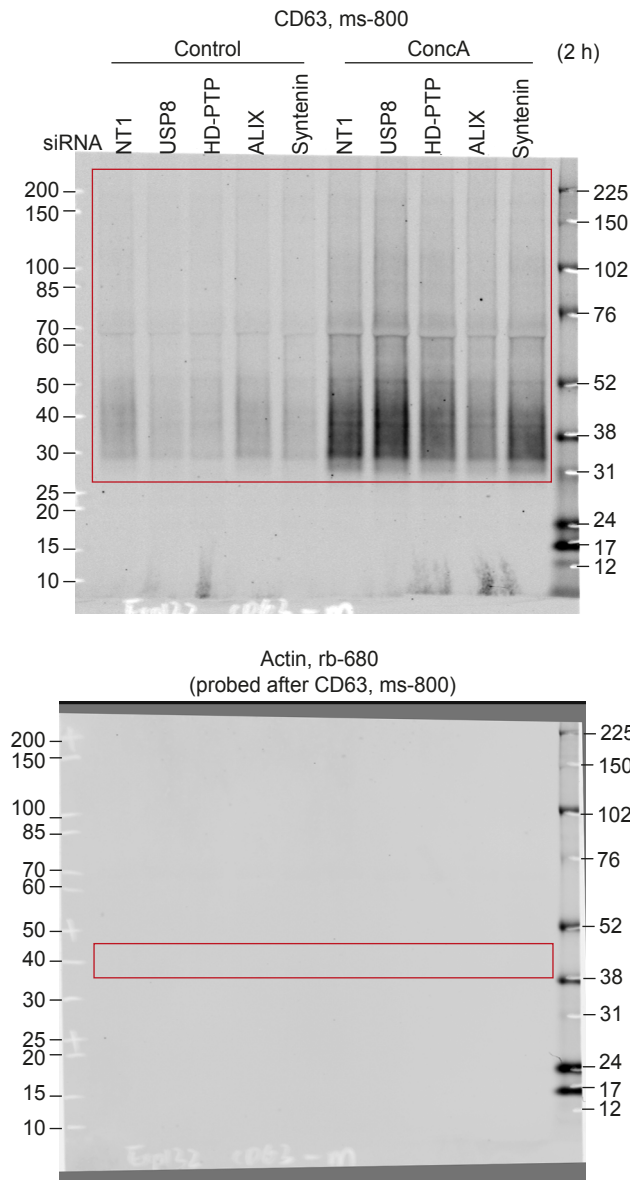

Supplement: SourceData FS3 — is the source file for Fig. S3. [file JCB_202312141_SourceDataFS3.pdf]

Supplementary Fig4A

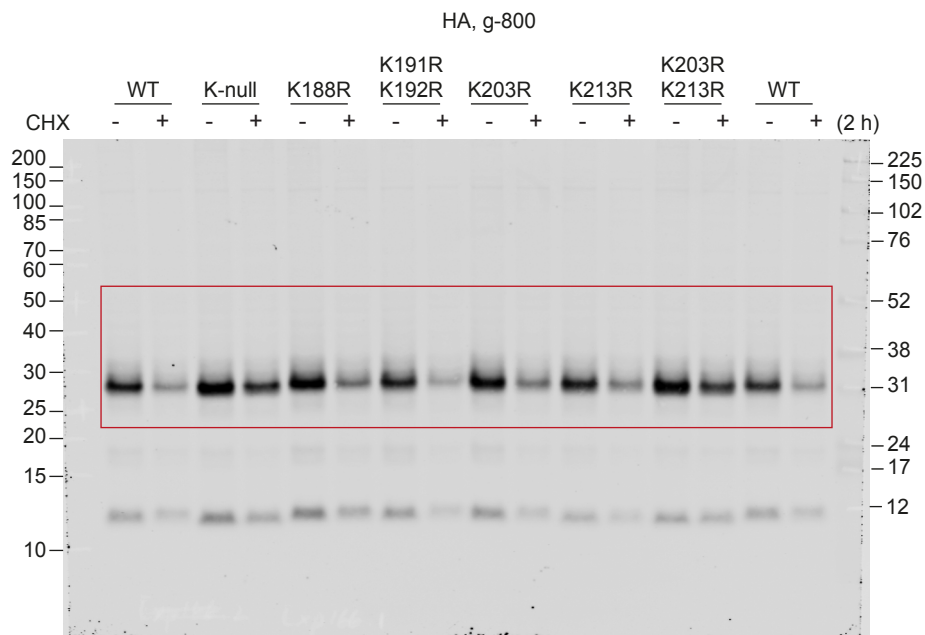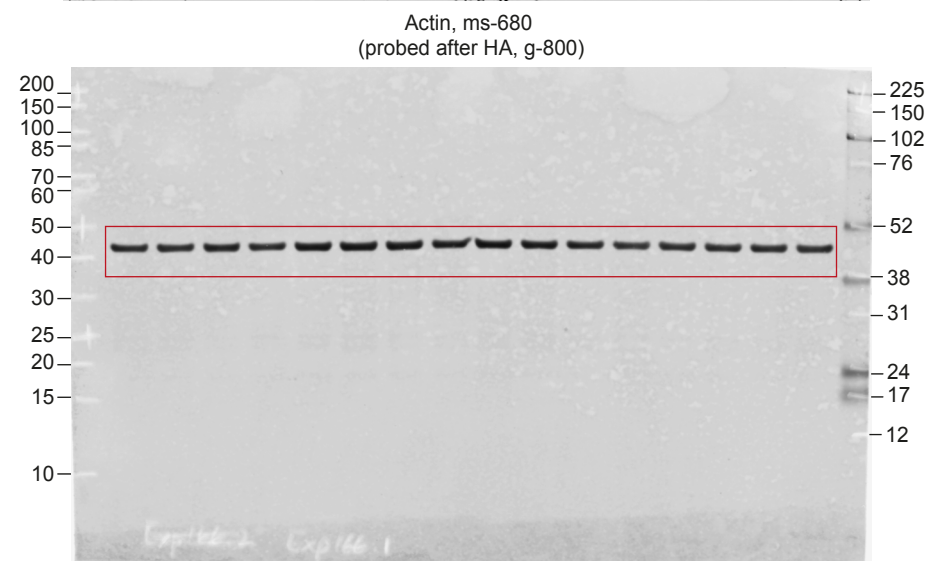

Supplementary Fig4C

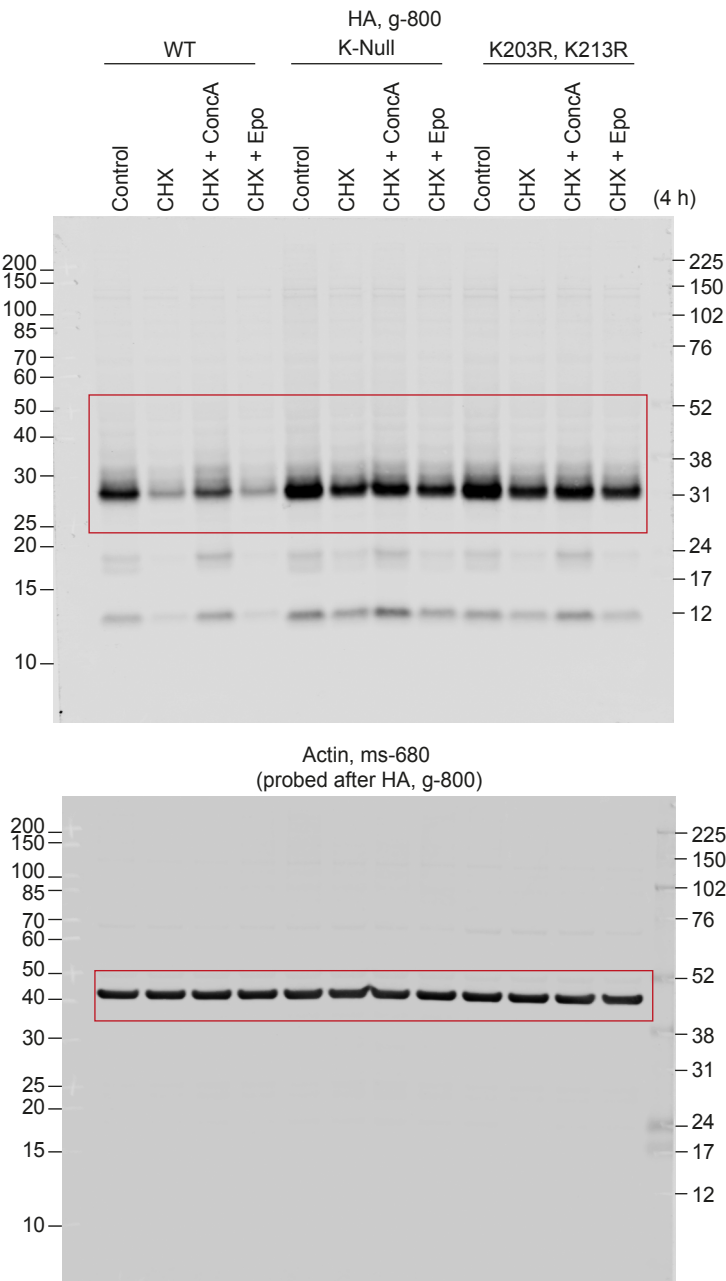

Supplement: SourceData FS4 — is the source file for Fig. S4. [file JCB_202312141_SourceDataFS4.pdf]
