# Supplementary material for: Rapid turnover of CTLA4 is associated with a complex architecture of reversible ubiquitylation
Source: J Cell Biol. 2024 Oct 15;224(1):e202312141. doi: 10.1083/jcb.202312141 (PMC11486831; doi:10.1083/jcb.202312141)

Supplemetary Fig2A: A2058

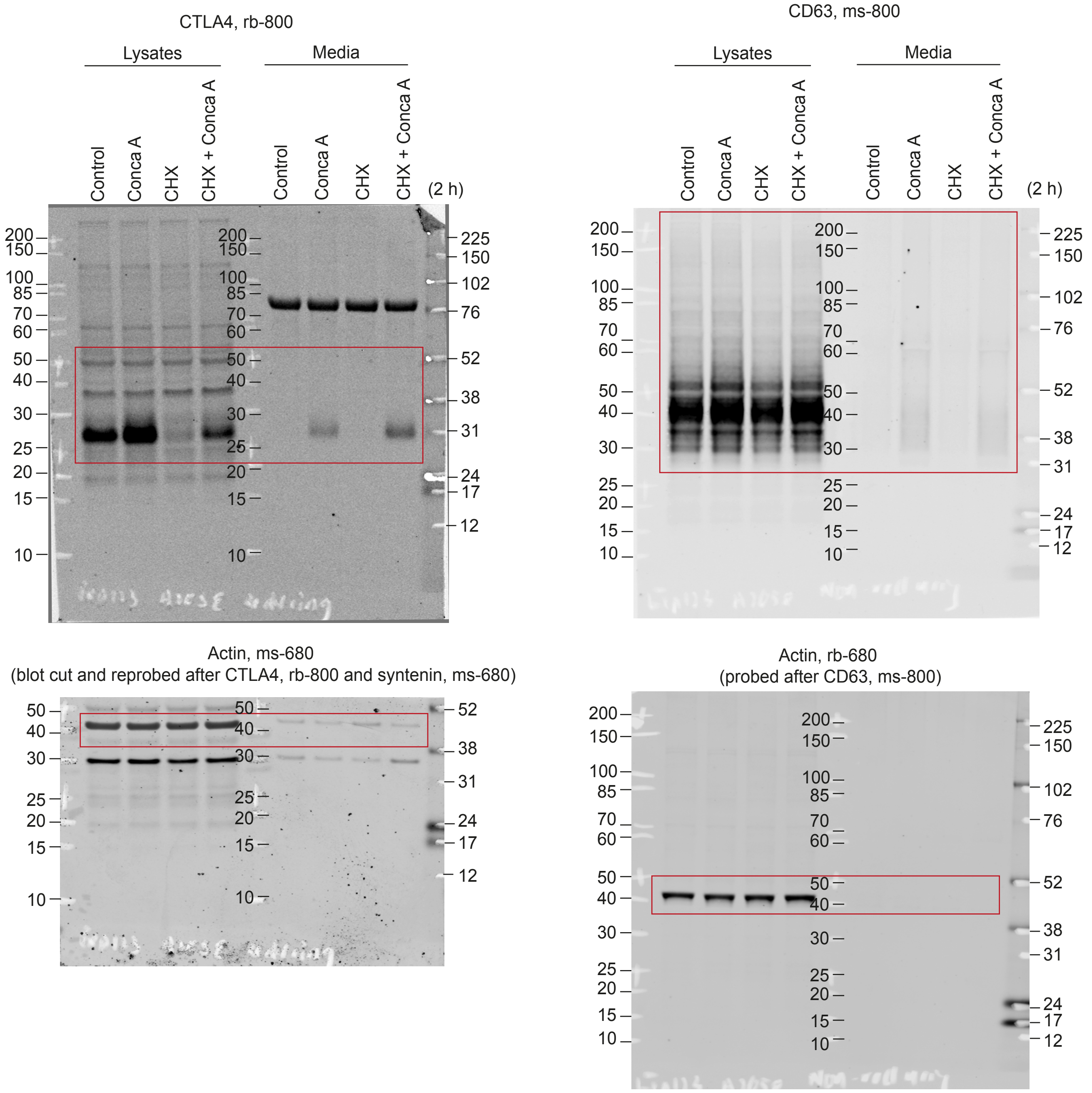

Supplemetary Fig2B: HeLa S3 FlpIn CTLA4-HA

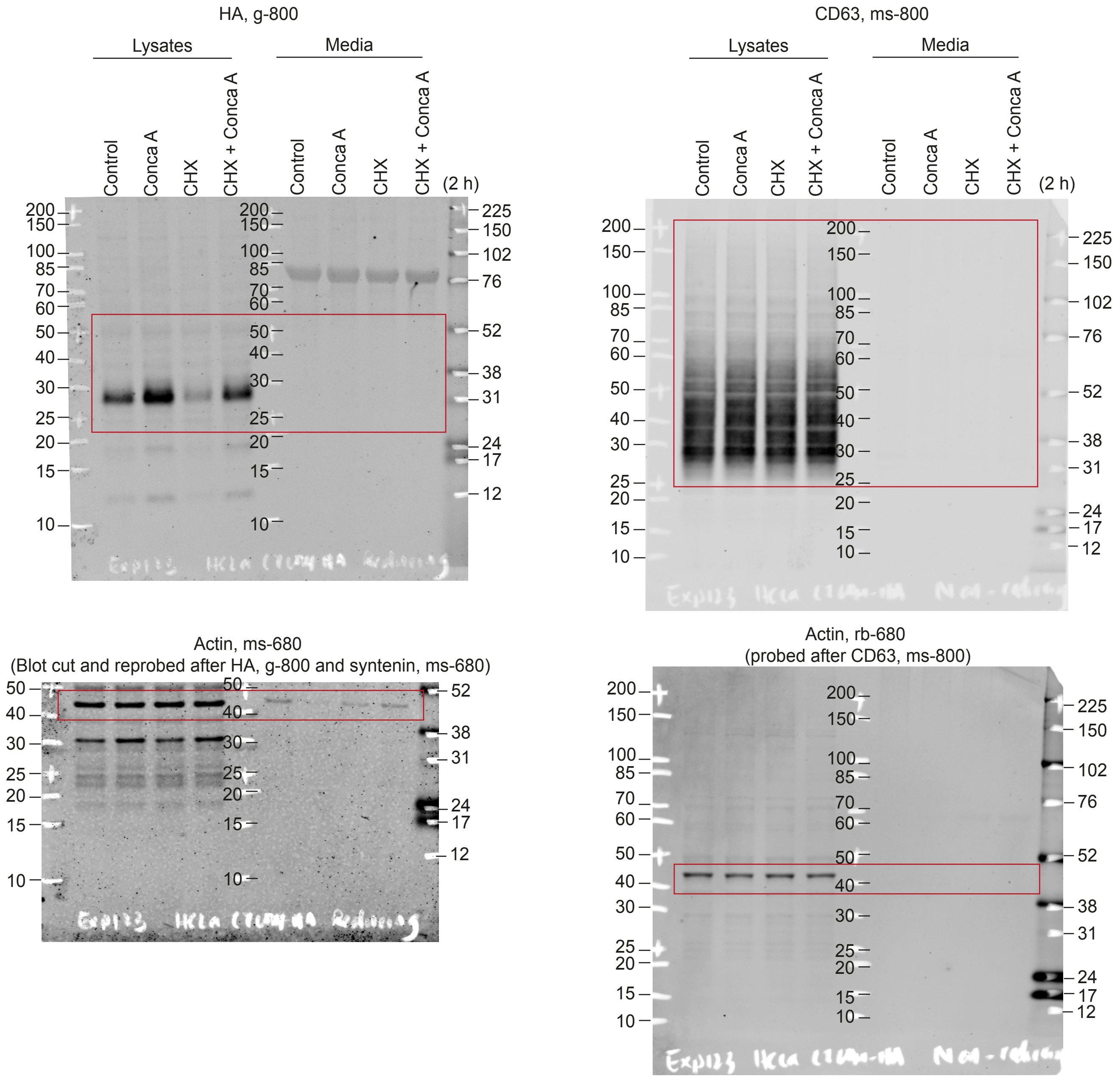

Supplemetary Fig2C: HeLa S3 FlpIn CTLA4-HA

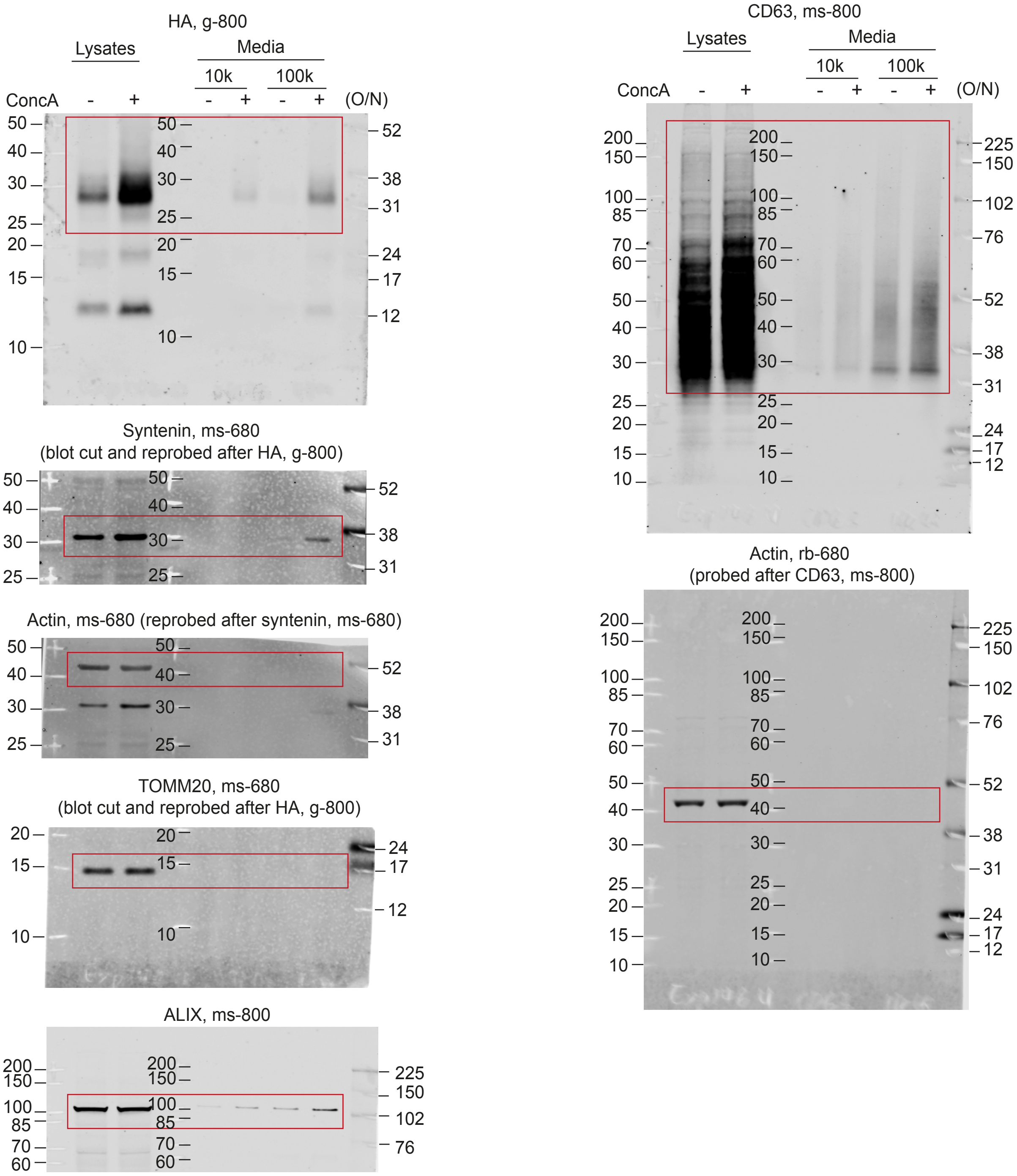

Supplemetary Fig2E: HeLa S3 Flp-In CTLA4-HA

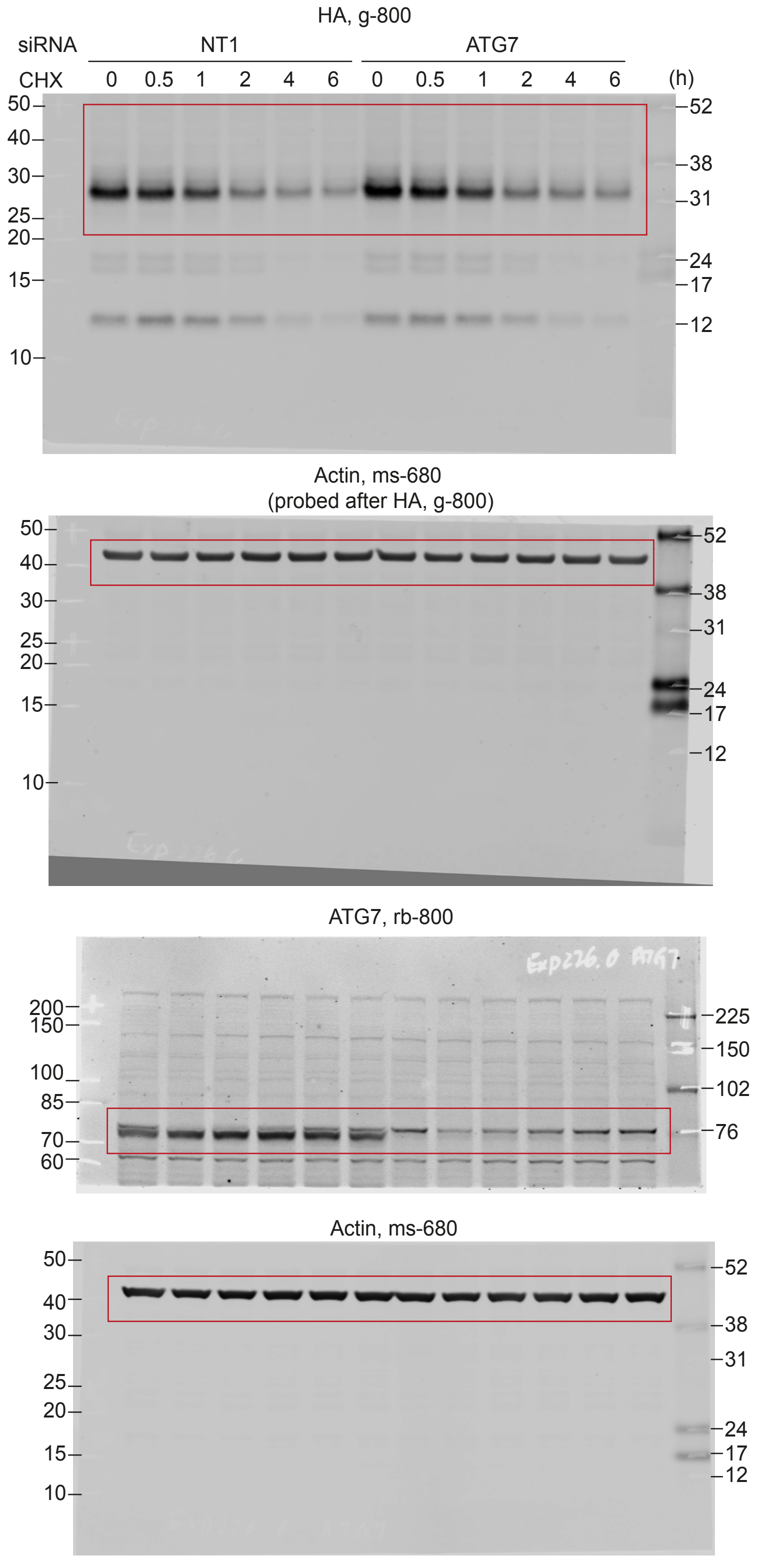

Supplemetary Fig2E: A2058 cells

CTLA4, rb-800

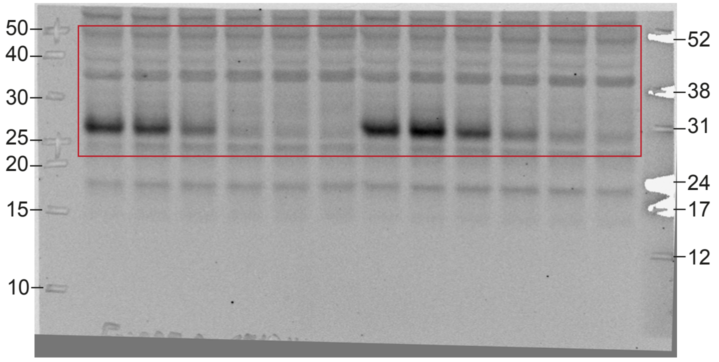

Actin, ms-680
(probed after CTLA4, rb-800)

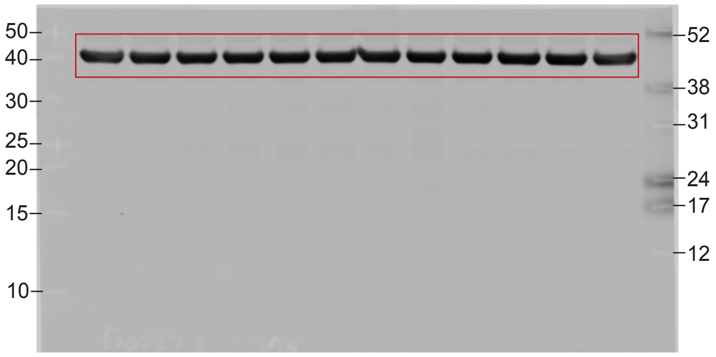

ATG7, rb-800

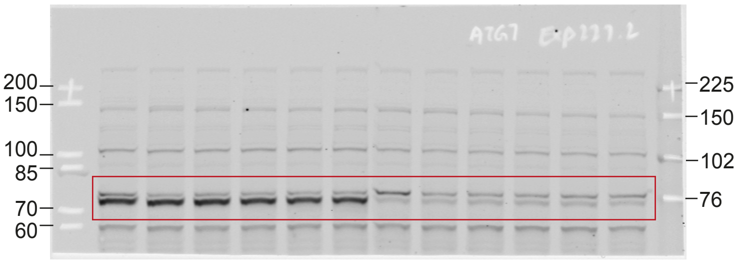

Actin, ms-680

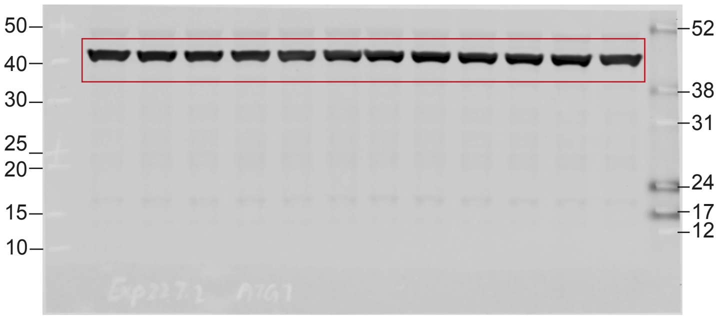

Supplement: SourceData FS2 — is the source file for Fig. S2. [file JCB_202312141_SourceDataFS2.pdf]
