# Supplementary material for: Rapid turnover of CTLA4 is associated with a complex architecture of reversible ubiquitylation
Source: J Cell Biol. 2024 Oct 15;224(1):e202312141. doi: 10.1083/jcb.202312141 (PMC11486831; doi:10.1083/jcb.202312141)

Supplementary Fig 5C

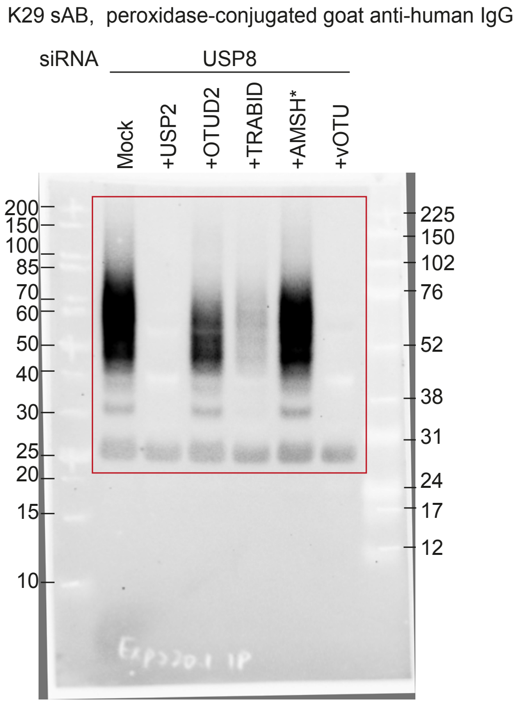

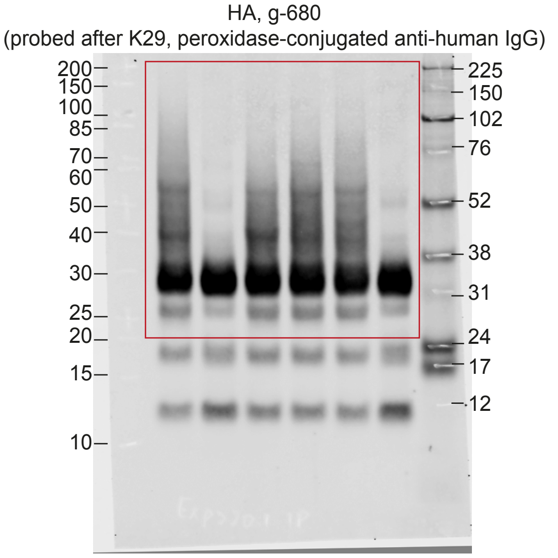

Supplementary Fig 5D

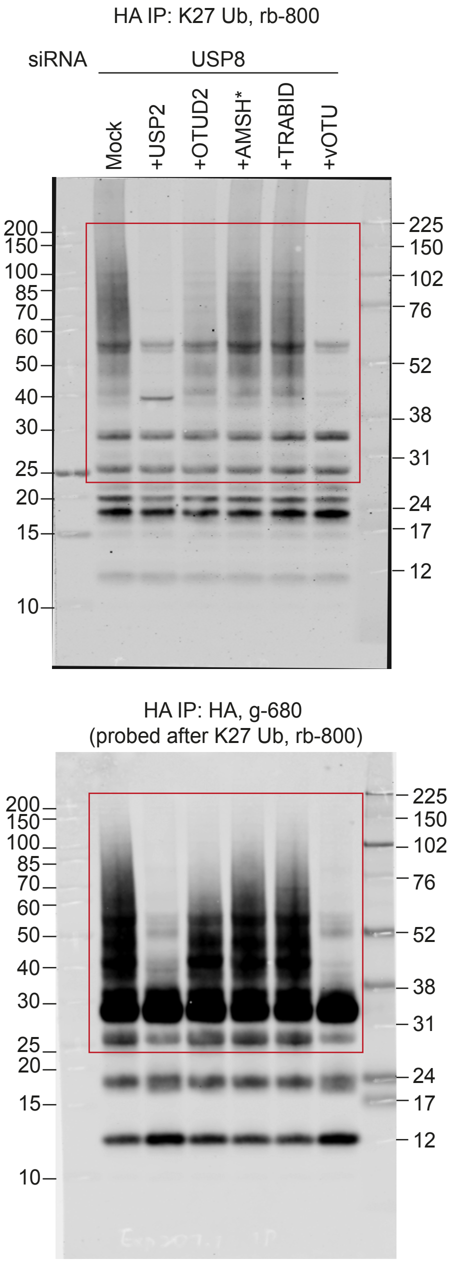

Supplementary Fig 5E

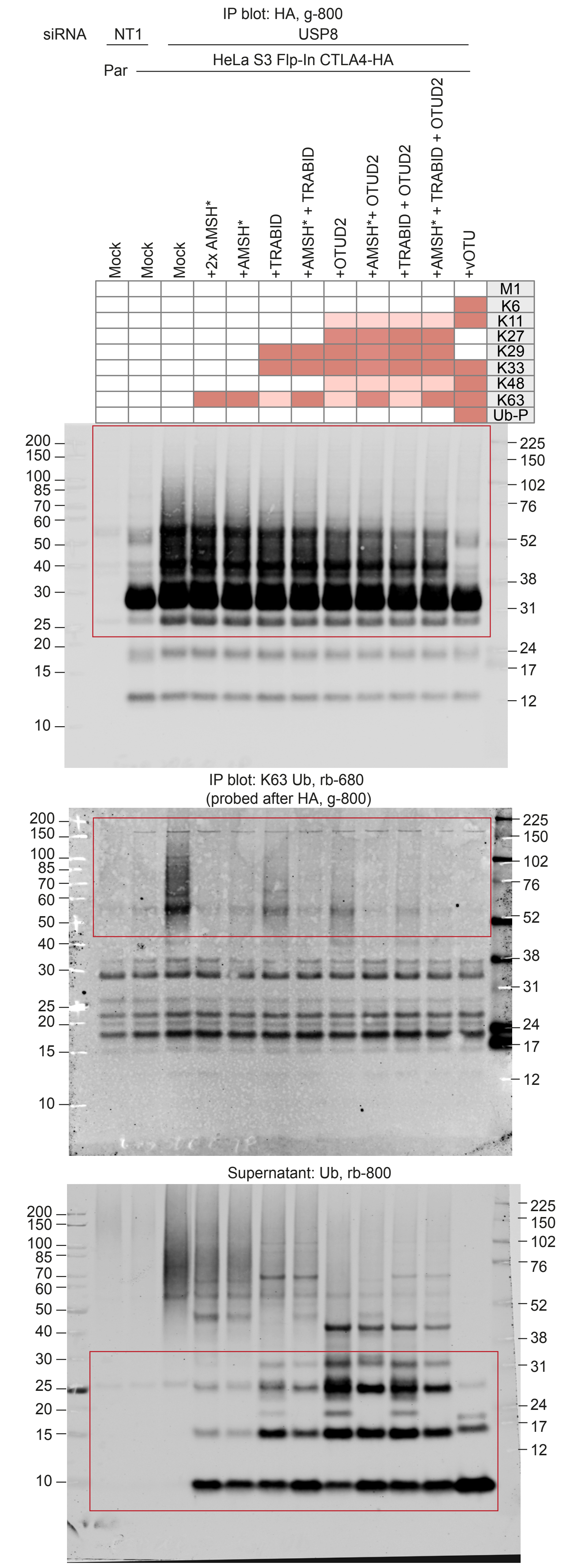

Supplement: SourceData FS5 — is the source file for Fig. S5. [file JCB_202312141_SourceDataFS5.pdf]
